# Understanding the relational culture of healthcare workplaces: a framework to guide improvement interventions, derived from a realist evaluation of neonatal care in Kenya

**DOI:** 10.1186/s12913-025-13686-6

**Published:** 2025-11-20

**Authors:** Claire Blacklock, Conrad Wanyama, Juliet Jepkosgei, Lisa Hinton, Jacob McKnight, Joyline Jepkosgei, Dorothy Oluoch, Sassy Molyneux, Bernie Hogan, Mike English, Geoff Wong

**Affiliations:** 1 Health Systems Collaborative, Nuffield Department of Medicine, University of Oxford, Peter Medawar Building for Pathogen Research 3, South Parks Road, Oxford, OX1 3SY UK; 2 Kemri-Wellcome Research Programme, Nairobi, Kenya; 3 Nuffield Department of Primary Health Care Sciences, University of Oxford, Oxford, UK; 4 Oxford Internet Institute, University of Oxford, Oxford, UK

**Keywords:** Hospital, Neonatal care, Realist evaluation, Relational, Workplace, Implementation, Culture

## Abstract

**Background:**

The implementation, design, and process of healthcare interventions and quality improvement initiatives often neglect the social and relational components of work, recognised as essential to their effectiveness, in part because these features are under-theorised. This paper offers a new framework and theoretical lens (the GELLE Framework: **G**rouping, **E**mpowering, **L**eading, **L**earning, **E**quipping) to help conceptualise complex relational workplace culture derived from realist evaluation.

**Methods:**

In-depth studies were undertaken in two low-resource urban hospital sites in Kenya, focusing on staff providing care to neonates. Data were collected using non-participant observations, in-depth interviews, and social network analysis. A realist analysis identified demi-regularities in the data, built context-mechanism-outcome configurations, and abstracted explanatory theory. Sense-checking of programme theory was undertaken with practitioner stakeholders.

**Results:**

The relational culture of healthcare workplaces can be understood in terms of five integrated theoretical domains of the developed GELLE Framework: (1) who individuals consider to be their peers and trusted colleagues (Grouping), (2) the aspects of formality and structure which influence the ability of specific individuals to act (Empowering), (3) how leadership influences relational culture (Leading), (4) how learners can be supported to become competent professionals (Learning), and (5) how physical order influences relational processes (Equipping).

**Discussion:**

Through structured explanation of the relational complexity and non-linearity of healthcare workplace culture, the GELLE Framework offers a practical theoretical lens for practitioners, researchers and programmatic teams to better understand the detailed contributions of relational culture to norms and change processes and therefore the implications for better design and implementation of improvement interventions.

**Conclusions:**

Efforts to improve the quality of healthcare are always implemented within the reality of the social and relational workplace. The GELLE Framework we developed offers a lens for better understanding of the relational culture of health facilities, identifying aspects of context amenable to intervention that should inform the design and implementation of improvement initiatives.

**Supplementary Information:**

The online version contains supplementary material available at 10.1186/s12913-025-13686-6.

## Background

Social and relational factors epitomise what makes healthcare complex, however, the healthcare facility as a relational workplace remains under-theorised [1, 2]. Though workplace culture has been branded both ‘culprit and remedy’ in healthcare reports [2], the details that constitute the relational aspects of workplace culture that influence care are not fully explored. Since relational and social factors are essential to ‘work’ [3], they must be better understood [1]. 

### Behaviour change and quality improvement

Crucially, the relational influences on norms and health workforce behavioural change must be more fully described, both at individual and collective level [4]. Indeed, with numerous quality improvement interventions seeking to adapt and change health workforce behaviours, the role of relational and social aspects is critical to realising intended benefits [5]. Likewise, where quality improvement efforts adopt participatory approaches, the influence of relational and social structures on narratives, power and action must be carefully considered.

Decision-making, individual and collective action and behavioural norms are fundamentally socially-mediated [6–11]. A fuller understanding of how, why, for whom and in what circumstances the relational workplace influences care delivery is thus required to imagine how ‘conditions for productive emergence’ [12] pg.104) might be fostered, and how the reproduction and transformation of social structures and systems occurs [8]. In the absence of explanatory conceptualisation a fundamental theoretical lens is missing in quality improvement efforts.

### Social mediation of norms in neonatal care in Kenya

Neonatal units in Kenya, as in many low- and middle-income settings, provide multidisciplinary care to manage highly complex clinical needs, in an environment of long-standing under-resource, overcrowding, expectations for training, and low nurse to baby ratios [13–15]. In such settings where resources are scarce and the workforce severely overstretched, ‘practical norms’ (i.e. the patterned behaviours of staff) can deviate significantly from both official and sociocultural norms, driven by necessity and regulated through relational means [16]. 

To address some of the technological and competence deficits that undermine delivery of high quality care, interventions such as the NEST360 programme [17] are being implemented. To complement and optimise such efforts, the complexity of social and relational processes within the diverse workforce must also be considered [4, 18]. 

### Understanding complexity using theory

Realist evaluation offers a useful approach to address the challenges of studying the informal, tacit, and often hidden nature of the social and relational world [19–21] to develop theory with explanatory power from relevant data [22]. Semi-regular patterns in the data (i.e. demi-regularities) are sought, to develop arguments of causation in the form of context-mechanism-outcome configurations (CMOCs), from which explanatory programme theory is abstracted [23, 24]. Causative explanation permits features such as non-linearity and feedback loops to be identified, and interdependence characteristic of complex adaptive systems to be represented. As such, theory can reflect social and relational complexity, whilst at the same time making details and processes distinct [25]. 

Relevant sociological theory has informed the work presented in this paper, particularly social network theory [7, 26]. Indeed, in advance of this study a realist synthesis of 75 documents that used social network analysis, was undertaken to develop an initial programme theory (IPT) and to guide protocol development and initial drafts of study tools (Box 1) [27]. 

**Box 1:**
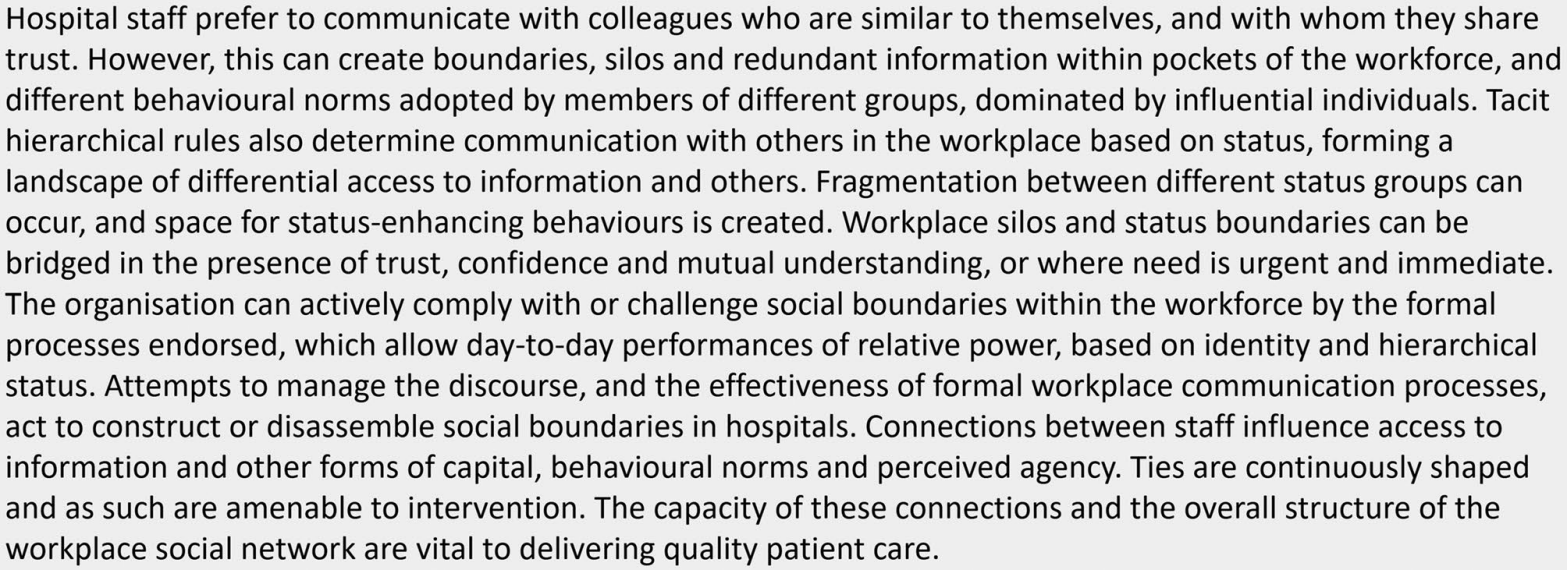
Initial Programme Theory (reproduced from Blacklock et al. [27])

## Aim

The aim of the realist evaluation presented in this paper was to explore how, why, for whom and in what circumstances, features of health systems ‘software’ [3] (e.g. values, norms, relationships) between health workers of all types caring for neonates in Kenyan hospitals, influence care processes being targeted by improvement efforts.

## Methods

This realist evaluation is part of the larger Pathways Study. The full study protocol is published elsewhere [24]. 

In brief, the Pathways Study realist evaluation was a study of neonatal care delivery in two urban hospitals in Kenya, one facility being much larger than the other, and both functioning as teaching and learning sites (see Table 1). Appropriate permissions and consent were obtained and the study was approved by ethical review boards in Kenya and the UK.

**Table 1:** Characteristics of study sites

Hospital 1 (H1)	Hospital 2 (H2)
Urban location	Urban location
Large teaching and referral hospital	Large county (district) hospital
Implementing site for NEST 360	Implementing site for NEST 360
Large neonatal unit (multiple sub-units/ rooms, including Newborn Intensive Care Unit)	Smaller neonatal unit (single unit/ room)
Clinical learning environment for non-specialist trainees (nursing) and specialist and sub-specialist paediatric and neonatal trainees (nursing and medical)	Clinical learning environment for non-specialist trainees (junior medical officers/ interns, nursing, non-physician clinicians, allied health professionals)

**Fig. 1:**
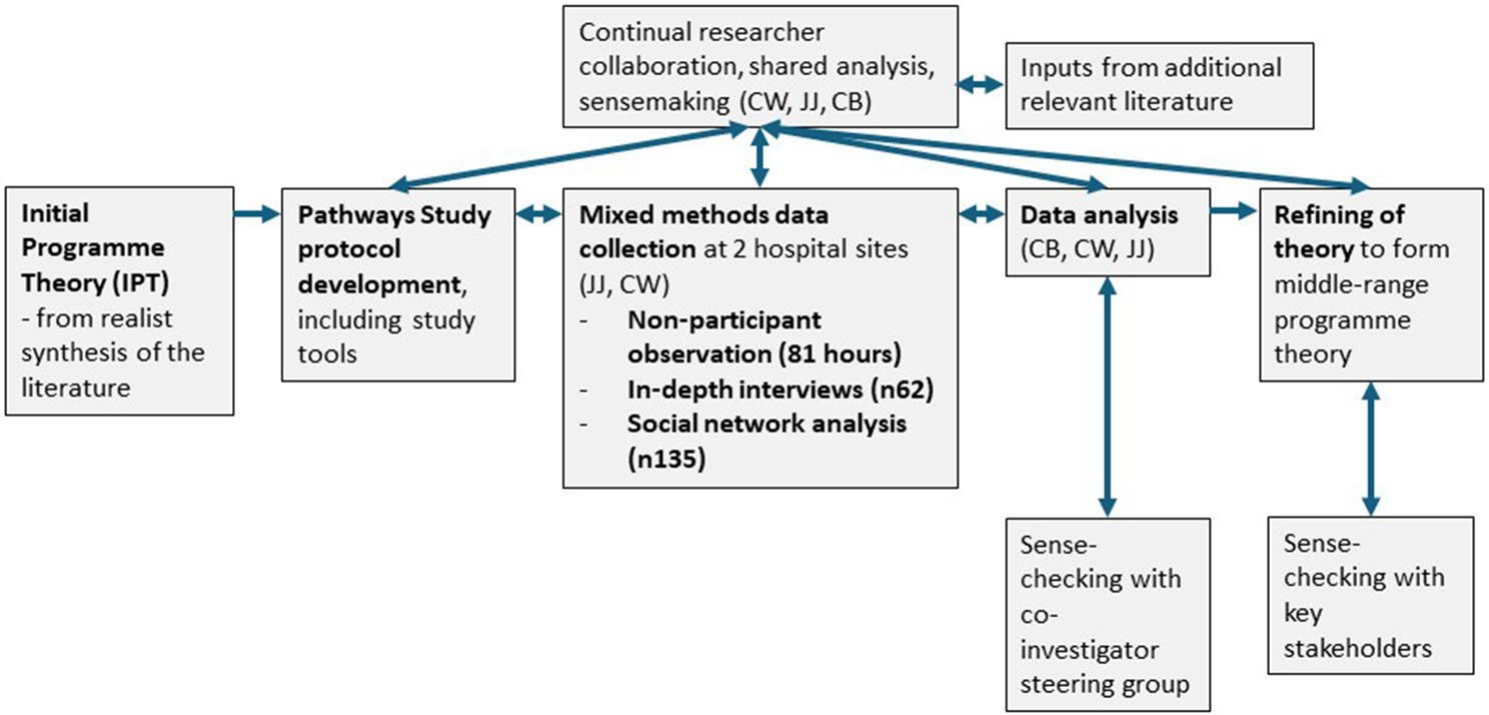
Process of programme theory development. (adapted from Wanyama et al. [24])

The initial programme theory (IPT) [27] was refined by collecting and analysing data to develop middle-range programme theory for the specific setting of neonatal care in Kenya. Since the IPT was developed from literature mostly from high-income settings and which employed social network analysis as a methodology, data collection was intentionally exploratory [24, 27]. As such, data collection sought to confirm, refute and refine the IPT and permit necessary expansion of the programme theory. Throughout the study duration, the core research team (CB, CW, JJ) met weekly to discuss the practical research process, emergent findings, analysis and interpretation, relevant literature, positionality, and ethics (Fig. 1).

Data used in programme theory development were collected using the following methods: (1) Non-participant observation, (2) in-depth interviews, and (3) social network analysis (SNA). Observation and SNA data informed sampling for in-depth interviews, to maximise variation amongst participants.

### Non-participant observation

With permission from the hospitals, two researchers (JJ, CW) spent time on the neonatal unit (H1 and H2) and maternity unit (H2), observing the environment, activities and interactions of staff, and unit-level meetings. A prompt sheet was used to guide observations, informed by the IPT, relevant literature and inputs of experienced colleagues. Researchers aimed to capture variation in the sampling of observations, for example different shifts (i.e. morning, afternoon, night shifts) and by attending meetings such as continuing medical education and nursing handovers.

Researchers recorded observation field notes, which were typed into MS Word. The observation notes were shared within the core research team (CW, JJ, CB) and areas for clarification or for further observation were annotated by CB, and responses (as well as reflections, specific assumptions, and subsequent related observation notes) were added by JJ and CW. Following this, the notes and all annotations were imported into NVivo software version 12 for analysis.

### In-depth interviews

A purposive sample of staff working in the neonatal unit (H1 and H2) and maternity (H2) were recruited to participate in in-depth interviews, aiming to maximise variation based on characteristics such as age, profession, gender, and observed social position within the unit. Interviews were around 60 min in duration and were conducted by researchers (JJ, CW) in a quiet location within the hospital. Participants were interviewed at a convenient time, to avoid interference with clinical care. A semi-structured interview guide (see Online Supplementary File) was used to explore experiences of the relational workplace. Interviews were audio-recorded and transcribed verbatim. Field notes were kept and typed by researchers (JJ, CW). Transcripts were checked for accuracy against audio files and de-identified by CW, and the final version made available to the core research team (CW, JJ, CB) and uploaded to NVivo for analysis.

### Social network analysis

All staff and students on the rota in the neonatal unit (H1 and H2) and maternity (H2) during a specified time period (1 week in H1, and 1 month in H2) were invited to complete a social network analysis questionnaire, aiming for complete networks. The questionnaire was administered face-to-face by a researcher (JJ, CW) and comprised demographic questions followed by scenarios in which the participant was asked to name who they would seek for advice or support (with no limit to number of nominees), across four domains of social support (Online Supplementary File) [28]. The researcher-administered questionnaires were conducted in a quiet location within the hospital, at a convenient time for the participants. For each scenario, the participant was asked to physically place the names (on sticky notes) of those they would seek for advice or support onto a sheet of paper [29]. The researcher (CW, JJ) recorded the study ID code of the participant and nominees onto an Excel template. The number of scenarios was reduced in H1 from 9 to 4, in recognition of time pressures on staff. Completed Excel templates were made available to the core research team for analysis.

### Analysis

**Fig. 2:**
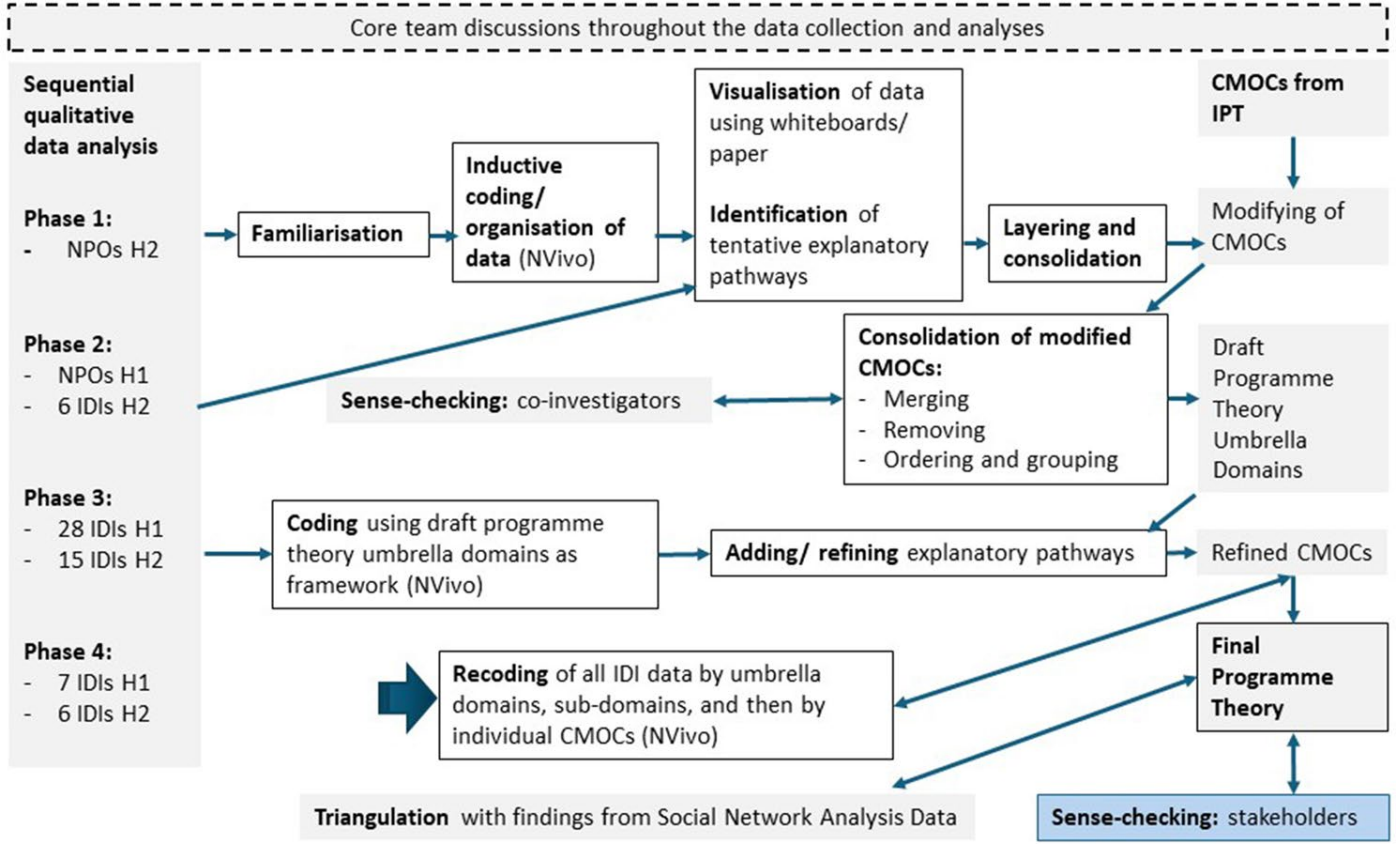
Analysis of mixed methods data in realist evaluation. (NPOs, Non-Participant Observation; IDIs, In-Depth Interviews)

Though informed by the IPT, initial data analysis to identify explanatory patterns (i.e. demi-regularities) was inductive, recognising differences in setting and methods, compared to the literature included in the previous realist synthesis. Qualitative data were analysed in a sequential manner (see Fig. 2 below, left), with initial familiarisation and inductive coding of observation notes, followed by seeking of explanatory patterns within the data (e.g. through visualisation using whiteboards), and refinement of CMOCs, before abstraction to programme theory [23, 30]. Explorative familiarisation and inductive coding of initial interview transcripts was undertaken, however individual interview data were eventually added sequentially, to enable the development of theory that included explanations of the influence of participants’ social position on outcomes. The quantitative social network data were first cleaned in Excel and then imported into Gephi version 0.10.1 for analysis. The data were explored using sociograms to visualise communication networks for different types of social support, and metrics were calculated and compared, specifically in-degree centrality and clustering coefficient [7, 26, 31]. SNA findings were used to triangulate CMOCs developed from qualitative data, and interim emergent SNA data informed both interview sampling and interpretation of qualitative data.

The IPT was confirmed, refuted and refined at the CMOC phase (see Fig. 2 below, top right). Context-mechanism-outcome configurations (CMOCs) were modified from the IPT and additional CMOCs developed from the qualitative data. Draft CMOCs were sense-checked with experienced co-investigators who were familiar with the study setting, and programme theory was abstracted. This was further refined as more data were added to the analysis, and finally all interview data were re-coded by CMOC (Fig. 2 below, bottom centre).

### Stakeholder sense-checking feedback

The final programme theory was sense-checked with stakeholders in Kenya, including from the two study sites. Stakeholders attended a workshop, during which findings from the Pathways Study [24] were presented, with the opportunity to give feedback on the programme theory in the form of written feedback and group discussion, and make recommendations for action.

## Results

### Participation

Participation rates are shown in Table 2, and consisted of 81 h of observation, 62 in-depth interviews, 135 SNA questionnaires and 34 stakeholders. Both hospital sites were highly challenging workplaces, with severe understaffing and high patient numbers. Researchers were sensitive to these challenges throughout the study and data collection activities were structured accordingly to minimise disruption, however this may have influenced participation rates (e.g. social network analysis).

**Table 2:** Participation in study

	Non-participant observations (NPOs)	In-depth interviews (IDIs) (*n*)	Social network analysis (SNA) (*n*)	Stakeholder event (*n*)
H1	40 h observation (7 shifts, 1 meeting, familiarisation)	35	76	34
H2	41 h observation (6 shifts, 2 meetings, familiarisation)	27	59	

### Explanatory arguments underpinning the programme theory

Realist evaluation of data retained 24 CMOCs from the IPT and identified 246 further detailed explanatory CMOCs from the data (see Supplementary Online File for full table, including illustrative quotes from interview data). These detailed CMOCs (n270) were summarised as 39 ‘umbrella’ CMOCs (presented in the section below). The evolution of CMOCs from the IPT to the final programme theory is presented in the Supplementary Online File.

### Middle-range programme theory – GELLE framework

**Fig. 3:**
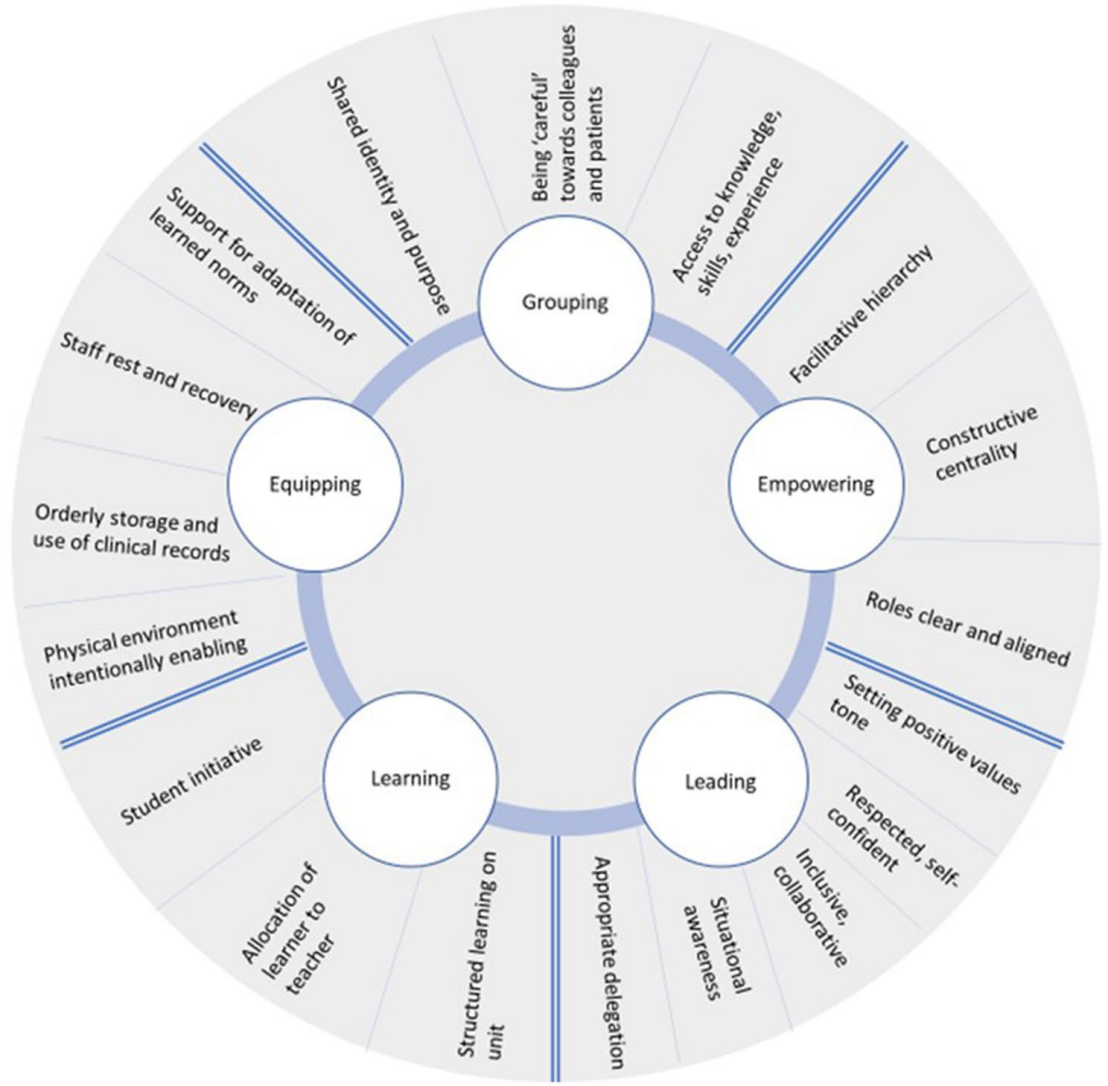
The GELLE Wheel

The relational workplace can be understood in terms of the GELLE Framework, developed from the CMOCs (summarised in Fig. 3: The GELLE Wheel):

G) Who individuals consider to be their peers and trusted colleagues (Grouping).
E) The aspects of formality and structure which influence the ability of specific individuals to act (Empowering).
L) How leadership influences relational culture (Leading).
L) How learners can be supported to become competent professionals (Learning).
E) How physical order influences relational processes (Equipping).

A summary of the findings of the realist evaluation is presented in the narrative below, organised by domains and sub-domains of the GELLE Framework. In addition, a table of Umbrella CMOCs (i.e. abstracted summary CMOCs) is presented for each sub-domain. Where relevant, supporting literature is cited throughout the results section, to indicate analogy with specific aspects of the programme theory.

*GELLE is an explanatory conceptual framework for understanding structuration *[8]* of the relational workplace between staff providing neonatal care in Kenya*,* derived from realist evaluation. GELLE domains and sub-domains are abstracted from context-mechanism-outcome configurations*,* which are presented in full in the Online Supplementary File.*

### G - Grouping

*Grouping* describes whom individuals consider to be their peers and trusted colleagues, based on their observations of: (1) shared identity and purpose, (2) being ‘careful’ towards colleagues and patients, and (3) access to knowledge, skills, experience. Within this domain are seven Umbrella CMOCs (Table 3), abstracted from more detailed CMOCs, derived from the study data (detailed CMOCs underpinning Grouping domain are reported in full in Supplementary Online File).

**Table 3:** Umbrella CMOCs for grouping domain

Sub-domain	Umbrella CMOCs
Shared identity and purpose	**U1**: When a group of individuals within the unit share identity/ purpose (C) they will trust and respect one another (O) because they perceive that they share a common representation and values (M). **U2**: When parallel cliques operate in a unit (C) negative impacts on staff and patients can occur (O) due to poor communication and teamworking (M).
Being ‘careful’ towards colleagues and patients	**U3**: When a colleague displays they are ‘careful’ towards their co-workers and also patients, in the way they interact and communicate (C), individuals will seek support and advice from this colleague (O), because they feel trusting towards them and comfortable approaching them (M). **U4**: When a colleague displays ‘lack of care’ towards their co-workers and also patients, in the way they interact and communicate (C), individuals will avoid this colleague (O), because they do not trust them, consider them a poor team player and ‘rigid to change’, and/or fear they will respond with incivility. (M). **U5**: When students are recipients or witnesses of incivility by unit staff (C) students will disengage, show peer-reliance and experience poor learning (O) because they will fear staff and will feel discouraged (M).
Access to knowledge, skills, experience	**U6**: When a colleague is identified as having knowledge, status, technical skills or experience (C) they will be sought for legitimate/ defensible information and help with decision making by individuals (O) because their advice is valued as trusted and authoritative (M). **U7**: When a colleague is identified as having poor skills (C) individuals will not seek their advice (O) because they will be deemed as unable to help (M).

**Key: U** (‘Umbrella’ CMOC), **C** (Context), **O** (Outcome), **M** (Mechanism)

#### Shared identity and purpose

Healthcare workers connect and communicate more readily with colleagues whom they consider to be like themselves in some way (e.g. in age, profession, scope of work, level of education), due to trust based on assumptions about broader commonalities [32–35]. Alikeness (e.g. in work ethic, beliefs) can also be identified between seemingly different staff (e.g. from different professions) when they work together, and when communication, confidence and mutual respect is strengthened within a team, and shared purpose and sense of belonging fostered [36, 37]. 

However, grouping of healthcare workers based on aspects of perceived shared identity can also lead to cliques and communication boundaries [34]. Those outside a group are easily identified (and judged) by its members and their input may be disregarded [38–40]. Peer-to-peer overreliance, redundancy of information, and insular sensemaking can occur [41–44], and perceived boundaries between different groups may obscure opportunities for unity. When parallel silos operate in the workplace, staff will be unable to communicate effectively, make shared decisions, or form unified plans.

#### Being ‘careful’[45] towards colleagues and patients

Conduct is perceived as evidence of character. An individual whose conduct makes others feel comfortable and trusting towards them (e.g. by listening, showing patience, offering explanation, humility) are sought for advice and support. Likewise, correction offered to others with encouragement and respect will more likely lead to acceptance and change. Staff who are physically present and considered ‘ready to help’ due to their supportive and collaborative attitude, will be sought in urgent situations. Reliable, hardworking and committed individuals will be popular, esteemed by colleagues and will attract delegated work. Likewise, those showing respect, joyfulness, and who refrain from gossip and negative talk, will emanate positive energy [46] and encourage imitation of admirable conduct in others.

However, some healthcare workers are avoided for support and advice-seeking, based on their displays of negative conduct [47], such as shouting, blaming, bullying, or being condescending or dismissive of others. Such conduct instils fear, intimidation, humiliation, and leads to colleagues feeling aggrieved, defensive, discouraged or disengaged [48, 49]. As such, alternative means of support are sought (e.g. peer-to-peer), and learning opportunities missed [50]. Correction in front of patients and a perceived lack of collaborative attitude also discourages engagement. Moreover, staff showing incivility, or who gossip, complain, are generally negative or unreliable in their work, or respond badly to correction [51], will be avoided. Offensive talk by individuals will lead to colleagues feeling aggrieved and developing coping strategies. Furthermore, normalisation may occur in the absence of clear expectations and corrective action [51–53]. 

#### Access to knowledge, skills, experience

Staff deemed to have relevant knowledge, skills or experience (e.g. seniors, specialists, those who display quality in their day-to-day practice) will be sought for advice and support, particularly by junior staff within their own professional group. Conversely staff who display poor skills will be avoided.

### E- Empowering

*Empowering* describes the aspects of formality and structure within the unit which influence the ability to act, within the following sub-domains: (1) facilitative hierarchy, (2) constructive centrality, and (3) roles clear and aligned. Within this domain are seven Umbrella CMOCs (Table 4), which are abstracted from more detailed CMOCs, derived from the study data (detailed CMOCs underpinning Empowering domain are reported in full in Supplementary Online File).

**Table 4:** Umbrella CMOCs for empowering domain

Sub-domain	Umbrella CMOCs
Facilitative hierarchy	**U8**: When a ‘facilitative hierarchy’ is fostered by leaders (C) there will be oversight and access to advice and information, with improved sense of belonging and group purpose (O) because there will be clear and intentional positive use of power (M) **U9**: When a ‘restrictive hierarchy’ is present (C) there will be poor oversight and access to advice and information, reduced sense of belonging and freedom, and a tendency to follow orders (O) because of tension, fear and othering (M).
Constructive centrality	**U10**: When an individual occupies a central position in the unit, or within a specific social group or clique (C) they will have greater influence on group norms (O) because they have more social power over group discourse and practices (M) **U11**: When an individual occupies a peripheral position in a social group (even if they have valuable contributions to bring) (C) they will not influence group norms (O) because they have little power to alter group discourse (M).
Roles clear and aligned	**U12**: When individual roles are clear (C) communication will be more effective (O) because go-to people with the necessary delegated authority are more easily identifiable (M) **U13**: When individual roles are unclear (C) there will be role evolution, duplication, conflict, inappropriate delegation, etc. (O) because of confusion within the team about responsibilities and boundaries (M). **U14**: When roles do not align with the skills, capacity or perceived professional identity of staff (C) there will be frustration, overwhelm, and informal role filling (O) because of perceived mismatch by staff (M).

**Key: U** (‘Umbrella’ CMOC), **C** (Context), **O** (Outcome), **M** (Mechanism)

#### Facilitative hierarchy

Where hierarchical information flows are clear and operating in a context of psychological safety [54], relevant information can be sought, received and shared [55, 56], including through social media applications such as unit-level WhatsApp Groups. Information brokers, gatekeepers and go-to people are easily identifiable [57] and staff and students are included in hierarchical communication flows, enhancing sense of belonging.

Conversely, unclear and poorly functioning information flows and processes lead to conflict, avoidance and circumvention, and lack of consensus and collaboration [38, 39]. Fragmentation at different levels of a hierarchy can lead to highly siloed identities and trust, exacerbated by feeling undermined and frustrated [38, 40, 47, 58, 59]. Challenge may not be permitted within a restrictive hierarchy, with actions and decisions based on authority [49, 60]. 

#### Constructive centrality

Within the hospital unit overall, staff with diverse or supportive roles (e.g. chief resident, nurse team leader) are often central to the overall communication network. Within sub-groups, individuals in central social positions (i.e. who are more connected) have greater power and influence over group norms [61–63]. 

Conversely, staff on the social periphery hold little influence on norms [62], and may deviate in their behaviours or disengage if they oppose central individuals. When staff work independently from others, even due to conscientious work ethic, they isolate themselves. Whilst this may improve a peripheral individual’s feeling of control, it limits their influence and curtails scope for wider change, which may be greatly needed [62]. Others can also isolate peripheral staff, for example by excluding from information sharing or participation (e.g. allied healthcare professionals, juniors or students, individuals who are shy), leading to reduced sense of belonging and disengagement [40]. Peripheral individuals (who may be highly proficient) can hence be overlooked. Moreover, new staff or students who are isolated will experience poor professional socialisation and inadequate learning [9]. 

#### Roles clear and aligned

Clarity of roles enables more effective communication within a unit, as colleagues can more easily seek the most appropriate person [64]. However, difficulties can occur when a specific role-holder is absent, or when their performance is poor, or when competing pressures and tasks lead to overwhelm within a role-set [65]. 

When roles are unclear, evolution, duplication, conflict, inappropriate delegation (e.g. to students) and non-completion of tasks can occur, as individual responsibilities are poorly defined. Individuals who prefer certain tasks or activities (or who perceive a role vacuum), may evolve their own role scope [65] to encompass these and neglect less-desirable tasks, which must be picked up by other colleagues. This can lead to skewed roles, piecemeal care and deskilling, for example in clinical procedures.

Individuals who feel underutilised due to a mismatched or limited role, will experience unfulfillment, poor satisfaction and risk deskilling [66], and some may refuse to undertake tasks not perceived to be part of their role [36]. 

### L - Leading

*Leading* describes how leadership within the clinical unit influences the relational culture, within the following sub-domains: (1) setting a positive values tone, (2) respected, self-confident, (3) inclusive, collaborative, (4) situational awareness, and (5) appropriate delegation. Within this domain are nine Umbrella CMOCs (Table 5), which are abstracted from more detailed CMOCs, derived from the study data (detailed CMOCs underpinning Leading domain are reported in full in Supplementary Online File).

**Table 5:** Umbrella CMOCs for leading domain

Sub-domain	Umbrella CMOCs
Setting a positive values tone	**U15**: When leaders set a ‘positive values tone’ on the unit (C) it will be a more organised, positive and respectful place to work (O) because staff will normalise these values (i.e. will be encouraged to ‘follow-suit’) (M). **U16**: When leaders set a ‘negative values tone’ on the unit (C), normalisation of undesirable behaviours by staff will occur (O) because a reliable and consistent example of positive behaviours and attitudes is absent (M).
Respected, self-confidence	**U17**: When leaders are respected by staff/students due to the demonstration of esteemed attributes, and have self-confidence (C), they will be more effective in their role, and supported by the team (O) because they have created a ‘safe’ working environment (M). **U18**: When leaders are not respected by staff/students or lack self-confidence (C) they will be considered ineffective in their leadership role, and will face resistance from staff/students (O) because staff perceive them to be disengaged from others and/or overwhelmed (M).
Inclusive, collaborative	**U19**: When a leader is inclusive and collaborative in their approach (C), satisfaction, continuity and sense of teamwork will be enhanced on the unit (O) because team members will engage and participate (M). **U20**: When a leader fails to demonstrate an inclusive and collaborative approach (C) staff will disengage and not respect their leader (O) because they will feel excluded by poor or incompetent leadership practices (M).
Situational awareness	**U21**: A leader with good situational awareness (C) will support staff in the provision of quality care (O) because they better understand and respond to the needs of the team (M).
Appropriate delegation	**U22**: When a leader is clear about their own role and delegates appropriately to others (C) the leader will be less overwhelmed, more effective, and satisfaction of staff will improve (O) because they will be confident about which roles and responsibilities to delegate and whom to empower (M). **U23**: When a leader fails to delegate appropriately to others (C) their leadership will be considered ineffective and staff will feel frustrated and dissatisfied (O) because skills of the team are not being utilised, and the leader (and some staff) are overwhelmed (M)

**Key: U** (‘Umbrella’ CMOC), **C** (Context), **O** (Outcome), **M** (Mechanism)

#### Setting a positive values tone

When leaders display an honourable work ethic and act with kindness and humility, they model positive values on the unit [37, 46, 56, 64, 67, 68]. When a leader’s expectations are clear and consistent, these are more likely to be upheld by staff (e.g. structured and respectful handover). Staff will feel supported and empowered by leaders who facilitate effective supervision, timely correction and support for quality care, and who display an open attitude to learning [64, 69, 70]. 

Conversely, negative values are modelled and communicated (even if unintentionally) when a leader is perceived to be blaming, humiliating, policing, and acting with incivility and overwhelm. Undesirable behaviours can become normalised [51], with fear and discouragement of staff and students, and fragmentation [71]. Unclear expectations not upheld by the leader themselves, contribute to crumbling standards and futility and frustration amongst staff [72]. Lack of appropriate supervision and support lead to poor staff satisfaction and inconsistent quality of care.

#### Respected, self-confident

A respected leader will enjoy support of staff and students within the unit. When a leader is perceived to be working for the good of others, they will be considered approachable and an atmosphere of safety will be fostered [64]. Self-confidence in both their role and authority will help a leader to be more effective [73, 74]. 

When leaders feel undermined, they risk becoming overwhelmed and disengaged. Perceived inaction and role vacuums in leadership lead to frustration amongst staff, and can further worsen self-confidence of the leader themselves [75]. Moreover, when staff do not respect their leader, they will resist their inputs, leading to ineffectiveness due to low social power.

#### Inclusive, collaborative

An encouraging, inclusive [54] and collaborative leader will give staff and students confidence to engage and participate, due to sense of belonging and teamwork [32, 34, 37, 64, 68]. Inclusive and collaborative leadership mitigates over-reliance on individuals. Leaders can include staff and students by a facilitative and convenient structure and timing to meetings, and by integrating regular open floor opportunities to enable whole team learning (including management of online (e.g. WhatsApp) discursive spaces) [9]. 

Leaders who show favouritism or who are feared, will exclude others from engaging in specific aspects of work (e.g. ward round), and cliques may form, reducing the wider team’s capacity for collaborative working, learning and shared decision making.

#### Situational awareness

Proactive leaders will seek situational awareness and develop good understanding of their team (e.g. skills mix, consumables, patient care needs). As such they will identify needs, gaps and lapses, and provide as effective and timely support as possible (e.g. securing necessary supplies, tailored correction or supervision) [21, 39]. Consequently, the working shift will be smoother and staff will appreciate and feel valued by the leader [37]. A leader can also facilitate continuity and consistency across the whole team by proactive information sharing for collective understanding [36, 38, 56]. Effective oversight and provision of support will encourage staff to share information with their leader, and confidence in leadership will enable staff to focus on their own role, as they feel secure that other essential roles in the unit are being managed appropriately [65]. 

#### Appropriate delegation

A leader who clearly defines their own role [74] whilst effectively and wisely delegating [56], will be less overwhelmed. Effective delegation is possible when leaders are aware of the skills mix within their team. Staff can be empowered to undertake non-leadership roles and delegated leadership functions, enhancing personal satisfaction. Appropriate delegation and allocation improve wider team capacity for supervision, continuous learning, and delivery of quality patient care and influence staff satisfaction.

Conversely, when a leader is unable to appropriately delegate to others within the team, they will become overwhelmed and be unable to perform effectively [75]. Staff will feel frustrated as a result, and satisfaction will reduce because skills are underutilised and they are not trusted in leadership roles.

### L- Learning

*Learning* describes how those within the unit support learners to become competent professionals, within the following sub-domains: (1) structure to teaching and learning on the unit, (2) allocation of learner to teacher, and (3) student initiative. Within this domain are seven Umbrella CMOCs (Table 6), which are abstracted from more detailed CMOCs, derived from the study data (detailed CMOCs underpinning Learning domain are reported in full in Supplementary Online File).

**Table 6:** Umbrella CMOCs for learning domain

Sub-domain	Umbrella CMOCs
Structure to teaching and learning on unit	**U24**: When clinical teaching and learning on the unit is prioritised and structured (C), inclusion, encouragement, appreciation will be strengthened in the unit, and consistency in patient care will be supported (O) because common understanding within the team will be promoted, and authority ascribed to the training (M). **U25**: When administrative aspects of student and new staff experience are well managed, e.g. inductions, number of candidates, assessment processes, clear mentor, opportunities for attending CMEs, etc. (C) learning experience and a trusting learning environment will be enhanced (O) because expectations, support and authority are made clear from the outset (M). **U26**: When administrative aspects of student/ new staff experience are poorly structured or poorly implemented (C) the learning environment will be chaotic, with discord, frustration and inappropriate reliance on individual student initiative (O) because of lack of oversight and clarity of expectations and understanding (M).
Allocation of learner to teacher	**U27**: When students/ new staff are allocated to a specific member of staff for supervision (C), learning, trust and sense of belonging are enhanced (O) because of consistency and reliability (M). **U28**: When students/ new staff have inadequate clinical supervision (C) inadequate learning and adverse patient care can occur (O) because students may practice beyond their competencies (e.g. when they are unaware of errors made – ‘unknown unknowns’) (M) **U29**: When there is an assigned Clinical Instructor and/or Clinical Mentor (C) students will experience enhanced learning (O) because there is clear structure to the placement and a point of support (M).
Student initiative	**U30**: When learning is self-directed and relies on a student’s personal initiative (C) there will be a wide spectrum in student learning outcomes during clinical placements (O) due to differences in personal learning styles and motivations (M).

**Key: U** (‘Umbrella’ CMOC), **C** (Context), **O** (Outcome), **M** (Mechanism)

#### Structure to learning on unit

Rituals and norms on the unit communicate expectations around learning, for example the structure of educational meetings and clinical staff meetings (e.g. handover) demonstrate the value placed on knowledge sharing in the unit and set the tone for learning [76]. Educational meetings legitimise, communicate and disseminate information, promoting a common understanding of best practice within the team [76]. When ward rounds are structured in a collaborative manner [54], expectations of learning will encourage attendance, fostering shared awareness and decision making. Printed summary information on walls (i.e. ‘speaking walls’) can help to consolidate learning and provide timely reference for healthcare workers and trusted educational source for students rotating on the units.

Lack of structure and responsiveness of educational meetings and poor dissemination of learning, will lead to frustration, fragmentation and poor consensus within the team. Moreover, when insufficiently matched to learning needs, or insufficiently mandated, those for whom learning would be most useful may not participate, reducing potential impacts.

Structuring the orientation of students and new staff (e.g. induction) improves learning expectations and fosters teaching relationships on the unit. Medical staff, with entrenched norms of mentoring immediate juniors, follow professional expectations of teaching and learning, which are not so embedded in other professions. In the absence of a clear go-to person for learners, or when there is poor collaboration between student training facility and clinical unit, stress, misunderstanding and chaotic learning can occur, with some learners failing to achieve competencies.

#### Allocation of learner to teacher

When junior staff or students are allocated a supervisor, a supportive dyadic relationship is facilitated [77]. This will enhance satisfaction, learning, and sense of belonging, as supervision is integrated into routine patient care. Supportive supervision can mitigate fear associated with an intimidating and unfamiliar clinical environment, improving self-confidence and facilitating learning. Competencies in practical skills can be developed within a psychologically safe supervisor-supervisee relationship, where the learner is supported to be ‘hands-on’ for appropriate tasks [76]. 

When students receive inadequate clinical supervision, expected scope of practice is unclear, learning is likely to be poor, and they may practice beyond their competencies. Severe staffing shortage and the overwhelming clinical burden on staff can compound neglect of student supervision and teaching.

When available, an assigned Clinical Instructor enhances student learning on the unit, as students have clear expectations on their conduct, monitoring, and learning journey. The Clinical Instructor can also support student administration, pastoral care, and provide additional practical training in procedures.

#### Student initiative

When learning on the unit is primarily self-directed and reliant on personal initiative, there will be a spectrum of learning achieved by individuals, ranging from quickly-competent individuals, to those who attain minimal competencies. When students are proactive, busy nurses will be more likely to teach them.

### E - Equipping

*Equipping* describes how physical order within the unit influences relational processes within the workforce, within the following sub-domains: (1) physical environment intentionally enabling, (2) orderly storage and use of clinical records, (3) staff rest and recovery, and (4) support for adaptation of learned norms. Within this domain are nine Umbrella CMOCs (Table 7), which are abstracted from more detailed CMOCs, derived from the study data (detailed CMOCs underpinning Equipping domain are reported in full in Supplementary Online File).

**Table 7:** Umbrella CMOCs for equipping domain

Sub-domain	Umbrella CMOCs
Physical environment intentionally enabling	**U31**: When the physical environment is organised in an intentionally facilitative manner (C) desirable actions and practices will be implemented (O) because ‘the right way becomes the easy way’ (M). **U32**: When the physical environment is disordered and chaotic (C) there will be incivility and workarounds (O) because time will be wasted and staff will become frustrated (M). **U33**: When there is a high patient: staff ratio or overcrowding (C) there will be shortcuts, neglect of specific activities, and negative impacts on patient care and staff/student wellbeing (O) because staff will be overwhelmed, and will lack space/time to effectively carry out their roles (M).
Orderly storage and use of clinical records	**U34**: When patient records are kept in an orderly manner and/or are integrated into communication exchanges between staff (C) the quality and use of written records will improve and be maintained (O) because value and purpose of documentation will be enhanced (M). **U35**: When patient records are not stored in an orderly manner and/or their quality is poor (C) there will be workarounds, poor patient care, dissatisfaction, blaming, and further worsening of the quality of documentation (O) because of frustration and wasted time from delayed file retrieval, missing components of (or entire) files, and distrust (M).
Staff rest and recovery	**U36**: When staff are provided with a rest space with adequate facilities (e.g. fridge, lockers) (C) there will be improved staff satisfaction, and more relaxed communication with colleagues (O) because they will feel valued by the hospital, and will be able to step away from the busy clinical environment (M) **U37**: When staff are tired, hungry, and/or not provided with adequate rest facilities (C) they will not feel valued and will display incivility (O) because they will feel exhausted and will be required to adopt unsatisfactory workarounds (e.g. changing in corridor) (M). **U38**: When staff witness the suffering of babies and mothers, and death (C) they may seek social support, sharing, and debriefing with colleagues (O) because they are experiencing personal emotional pain (M).
Support for adaptation of learned norms	**U39**: When a member of staff transfers to a new unit and is not made aware of the unit-specific norms (C) they are likely to practice different norms to those practiced by unit staff, which may clash (O) because norms of individuals evolve to fit different workplace environment (i.e. availability of resources, size of unit, and numbers of patients etc.) (M).

**Key: U** (‘Umbrella’ CMOC), **C** (Context), **O** (Outcome), **M** (Mechanism)

#### Physical environment intentionally enabling

Intentional positioning of items and equipment on the unit can make ‘the right way the easy way’,(76^pg.452^) thus facilitating desirable actions, and communicating expectations. Staff can be supported to adhere to expected standards through ordering of the physical environment, leading to smoother working and enhanced satisfaction. Order will be maintained when staff feel collective responsibility, sense of belonging, and recognise the contribution of an orderly environment to patient care. Accessible physical reference information (e.g. protocols, guidelines, handover books, communication books), will assist staff and students in their work. Posting of relevant protocols, guidelines, and job aids on the walls in strategic places (and via smartphone apps) will provide trusted, clear, and timely reference and reminders, improving ease of work, continuity of care, and learning.

Tasks on the unit become difficult when staff must search for everything they need. When equipment is not readily available, scarce, in high demand or insufficient or non-functional, staff will feel frustrated, may display incivility, and will employ workarounds. Additionally, incivility may be directed towards those who are considered careless with precious items, e.g. gloves.

Insufficient staffing leads to overwhelm, shortcuts, workarounds, missed care, and managers will neglect managerial tasks to prioritise patient care roles. Likewise, when physical space is crowded, it is difficult for staff to carry out roles effectively. Some physical items in the unit can enhance communication, such as a unit telephone and a computer. Physical items such as tables whilst enabling the completion of documentation, can also contribute to interprofessional boundaries when different professions sit at different locations.

#### Orderly storage and use of clinical records

When patient records are kept in an orderly manner, the ‘right way becomes the easy way’,(76^pg.452^) respect for documentation is enhanced, and order and quality maintained. Integration of documentation into routine patient care activities (e.g. ward rounds, handovers), improves accuracy, function and enhances the perceived value of documentation. However, when the quality of written information is poor, staff will employ workarounds and documentation quality will degrade further, with staff and students relying on alternative means to receive and share information.

#### Staff rest and recovery

Protected areas for rest, not only make staff feel valued, but also provide opportunities for informal interactions away from the intense and emotionally demanding clinical environment (e.g. emotional support, debriefing), improving satisfaction [64, 76]. Conversely, when protected rest spaces are absent, staff can feel devalued (e.g. changing in corridor), and will lack opportunities for certain types of social support. Furthermore, when overwhelmed or tired and hungry, exhaustion can lead staff to display incivility.

#### Support for adaptation of learned norms

The clinical setting in which students and staff train and work, determines their norms of practice and workarounds learned and adopted, based on unit-specific factors (e.g. availability of equipment). Therefore, when staff transfer to a new hospital, these norms and workarounds must evolve to match those within the new unit. Indeed, differences in norms and workarounds may lead to conflict with new colleagues and students. Hence, exposure and ability to learn clinical competencies and norms is influenced by the resourcing of the clinical learning environment. Ultimately students will learn the workarounds which are modelled by staff on their clinical placements.

## Discussion

This paper reports the findings of a realist evaluation exploring the relational environment of neonatal care in Kenya, using mixed method data from two urban hospitals. The result is a conceptual lens through which to view the relational healthcare workplace.

In summary, social ties, or channels of communication and support between individuals are shaped by the following integrated domains (GELLE Framework): (1) who individuals consider to be their peers and trusted colleagues (Grouping), (2) the aspects of formality and structure which influence the ability of specific individuals to act (Empowering), (3) how leadership influences relational culture (Leading), (4) how learners can be supported to become competent professionals (Learning), and (5) how physical order influences relational processes (Equipping).

The GELLE Framework illuminates the human factors within healthcare complexity. Non-linearity is demonstrated [25, 78], for example by the far-reaching negative outcomes relating to incivility. Incivility, which may itself be a product of tiredness, hunger and overwhelm, permeates the GELLE theory. For example, colleagues may avoid the individual, students may preferentially consult their peers, and general negative emotional energy [68] may be felt in the unit. When displayed by a leader, others may not approach them for information, may not share their needs or concerns, and respect for the leader (and their expectations on staff) will dwindle.

However, seemingly small relational acts can also have widespread positive influence within the healthcare workplace [78, 79]. For example, showing kindness to patients and behaving in an approachable manner can influence enjoyment of work on the unit, information sharing, imitation of positive behaviours, and enhance student learning. Inclusive leaders [54] can likewise empower and encourage others through delegation, influencing job satisfaction, clinical care and workload. Hence, GELLE illuminates specific opportunities, whilst seemingly insignificant when viewed in isolation, that can have widespread influence on the whole [78]. 

By representing the relational workplace in conceptual form, the GELLE Framework facilitates more granular discourse around workplace culture, and the contribution and integration of specific influences, such as leadership [1]. As such, the GELLE Framework provides opportunity for reflection, and use as a catalyst for innovation within clinical teams around improving the relational and social aspects of work (see Box 2). The CMOCs within the GELLE Framework can additionally help to identify relational contextual targets for intervention, and hence offer contributions to theory-guided intervention design, as well as implementation and evaluation approaches. Thus, the GELLE Framework offers a practical theoretical lens for practitioners, researchers and programmatic teams to more fully consider the complex and non-linear contributions of relational culture to the change process. Using GELLE to emphasise which aspects of relational culture are targeted within the design and implementation of healthcare improvement interventions might be a helpful addition to the theory of change, particularly for complex interventions intending to change some aspect of healthcare workforce behaviour.

**Box 2:**
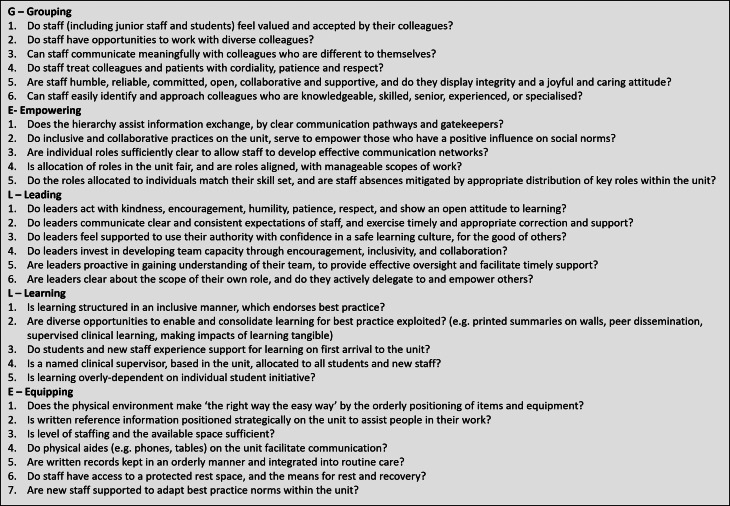
Application of GELLE Framework as reflective workplace tool

The programme theory should be further tested (i.e. confirmed, refuted, refined) in other settings, to assess transferability. Despite differences in setting and methodology, there is alignment between GELLE and the relational features identified in existing frameworks in the literature, for example the For Us (For Unit Safety) Framework, which describes the 7 features of safety in UK maternity units [76], and the ABC Framework of the core work needs of nurses and midwives in the UK [64], suggesting a degree of generalisability of core relational workplace factors and transferability of learning [4]. The GELLE Framework adds value to these by explaining granular causation and demonstrating the integration and non-linearity of relational acts.

### Strengths and limitations

Reporting of this realist evaluation follows RAMESES II guidelines (supplementary online file) [23]. The quality of the explanation provided by realist programme theory can be assessed, specifically using three criteria: (1) the theory’s *consilience* (ability to account for as much of the relevant data as possible), (2) its *simplicity*, and (3) *analogy* with existing theories. Better explanatory theories have greater consilience, simplicity and analogy. In these aspects, GELLE can be judged to be of higher quality [80]. Consilience and analogy are high, as demonstrated in the results and appendices. The GELLE Framework also achieves simplicity, in that highly complex social phenomena are summarised as five integrated theoretical domains. Understanding the relational whole is enhanced through the simplicity of five theoretical domains, and deeper dives into the details of accompanying narratives and CMOCs reveal greater conceptual clarity.

Throughout the Pathways Study, researchers practised reflexivity, considering their own positionality throughout data collection, analysis and interpretation phases. Furthermore, researchers paid careful attention to how study findings were communicated and hence represented in research outputs, particularly those relating to social networks within case study sites [81–83]. 

Limitations of the study included the time pressures on staff associated with the busy clinical environment, which were felt to reduce participation rates in the study. The data were also collected from neonatal care settings (including maternity in one site) in just two urban hospitals in Kenya, and data from other settings may differ and this may limit transferability of the programme theory. However, feedback from policy engagement activities suggested that findings are relevant to other clinical settings.

## Conclusion

This paper offers a conceptualisation of the relational culture of healthcare workplaces, by means of programme theory developed from realist evaluation of data collected in two hospitals in Kenya. The programme theory (GELLE Framework: **G**rouping, **E**mpowering, **L**eading, **L**earning, **E**quipping) explains how the relational workplace functions, and thus how reproduction and transformation of socially-mediated norms can occur. As such, the findings offer a practical theoretical lens and deeper understanding of the why, when, how, for whom and to what extent relational culture impacts on patient care, thus informing improvement efforts at healthcare facilities. The GELLE Framework provides a tool for identifying and reflecting on aspects of context amenable to intervention, that should inform the design and implementation of improvement initiatives.

## Supplementary Information

Below is the link to the electronic supplementary material.

Supplementary Material 1

## Data Availability

Illustrative supporting data are provided in the online supplementary file. Reasonable requests for additional de-identified data sets should be addressed to the corresponding author.

## Abbreviations

C: Context
CMOC: Context, Mechanism, Outcome Configuration
GELLE: Grouping, Empowering, Leading, Learning, Equipping
H1: Hospital 1
H2: Hospital 2
IDI: In-Depth Interview
IPT: Initial Programme Theory
M: Mechanism
NPO: Non-Participant Observation
O: Outcome
SNA: Social Network Analysis
U: Umbrella
