# Supplementary material for: Understanding the relational culture of healthcare workplaces: a framework to guide improvement interventions, derived from a realist evaluation of neonatal care in Kenya
Source: BMC Health Serv Res. 2025 Nov 20;25:1499. doi: 10.1186/s12913-025-13686-6 (PMC12632122; doi:10.1186/s12913-025-13686-6)
Supplement: Supplementary file 1 — Supplementary Material 1 [file 12913_2025_13686_MOESM1_ESM.pdf]

A: Inclusion and exclusion criteria

**Methods of data collection**	**Inclusion criteria**	**Exclusion criteria**
• Non-participant observation<br>• In-depth interviews<br>• Social network questionnaire	• Must work in the delivery room, post-natal ward, neonatal unit, or paediatric ward, or support the function of these units as part of their work within the wider hospital.<br>• Relevant staff of all cadres and grades will be included, for example nurse, doctor, midwife, nutritionist, clinical officer, patient care attendant, housekeeping, physiotherapist, orthopaedic technician, mortician, kitchen staff, laboratory staff, pharmacy staff, hospital administration.<br>• Nursing and midwifery students and final year medical and clinical officer students will also be included, to reflect their role in service provision.<br>• Must be aged 18 years or over<br>• Must be able to provide informed consent<br>• Must be available to participate in the study, without their participation adversely affecting to the functioning of the neonatal/maternity unit (e.g. before/after shift or during break)	• Does not work in, or provide any support to neonatal care delivery in the hospital<br>• Aged under 18 years<br>• Unable to give informed consent<br>• Unable to participate in study, without participation adversely affecting the delivery of neonatal care

B: In-Depth Interview Guide

PART A – for all participants

1. **How do you decide/choose who to ask for help or information at work?** *Additional prompts:* Can you give me an example? Why did you choose this person? What was it about them/you/the situation, which made you ask them? Does choice of colleague depend on the type of help or information you need? Which colleagues do you need to speak to on a day-to-day basis to undertake your role effectively? Why?
2. **Does discussion with a colleague at work change what you do/ your behaviour/ your decision making?** *Additional prompts:* Can you give me an example?
3. **Whose opinion(s) do you value most at work?** *Additional prompts:* Why? Anything about them/you/the situation?
4. **Are there any colleagues that you find difficult to speak to (ask for help from) at work?** *Additional prompts:* Why do you think you find it difficult to speak to (ask for help from) them? Anything about them/you/the situation? Are there any situations when you would speak to (ask for help from) them? Do you feel comfortable approaching senior (or junior) colleagues for help? Do you feel comfortable approaching colleagues from different professions for help?
5. **How do you select with whom to share your helpful information or knowledge (i.e. information that you have)?** *Additional prompts:* What kinds of information or knowledge do you share?
6. **Are there any colleagues with whom you find it difficult to share your helpful information or knowledge?**
7. **Are there situations where you have to ask for information or help but you cannot get it?** *Additional prompts:* Can you give me an example: situation/type of help/from who? What did you do about it: yourself/situation/the other person(s)? How does this make you feel?
8. **Do you feel staff members share information and knowledge freely at your hospital/unit?** *Additional prompts:* Can you give me an example? What type of information, knowledge or help would people be commonly seek from others, or share with others on a day-to-day basis? Why do you think this is so?
9. **What would help you better communicate with your colleagues at work?** *Additional prompts:* Would this improve patient care? How? Why?
10. **Can you always access the information you need in the workplace?** *Additional prompts:* Do you ever feel like you are missing out on information at work? Do you try to access the information in any other ways? For example: clinical guidelines, protocols, hospital policies, key communication messages from management to staff, information specific to the professional cadre, books, phone, WhatsApp groups, any other information?
11. **Do you use any other information sources/resources/courses/CPD to do your job?** *Additional prompts:* Can you give an example? How and when do you access this information?
12. **How could access to essential and reliable information be improved in the unit/hospital?** *Additional prompts:* Would this improve patient care? How? Why?
13. **Do you feel that you can always say and do what you think is best, in the workplace?** *Additional prompts:* Why/why not? Please give me an example. Do you feel like you do things differently or have a different outlook/attitude to some of your colleagues? How does this make you feel? Please give me an example. How does this make your colleagues feel? Does this impact on your patient care?
14. **What are your experiences of supervision/support/mentoring at work?**
15. **What (or who) do you feel has influenced you the most in the way you work?** *Additional prompts:* Can you describe this type of influence detail? How has this impacted your individual perception about your work? How has this impacted on how you provide care to your patients? How does this make you and colleagues feel?
16. **Do you feel there is anyone at work who influences the behaviour of your whole peer group?** *Additional prompts:* Who? Why do you think this is? Do you think you contribute to this person influencing your peer group? How?
17. **Are there individuals you prefer to be on duty with?** *Additional prompts:* Who? What is it about them that makes you want to only work with? What is it about you that makes you wish to work with them?
18. **Are there individuals you would prefer not to be on duty with?** *Additional prompts:* Who? What is it about them that makes you wish not to work with them? What is it about you that makes you wish not to work with them?
19. **What do you do if you receive contradictory advice at work?** *Additional prompts:* Where does the contradictory advice come from? Talk me through your thought processes in how you decide what you will do.
20. **What are the hospital's 'values'?** *Additional prompts:* How are these expressed to you? Do these 'values' change what you do at work?
21. **Can you describe what is known and said about this hospital by the general public?** *Additional prompts:* Do you think this perception is also shared by some (or most) staff members? What do you think has contributed to this? How has this influenced the way you and your colleagues provide care to your patients?

For non-nurse staff and students, interview ends here.

PART B: additional questions for nurse managers, all nurses, plus student nurses

20. **What is your experience with new graduate nurses in providing care to neonates?** *Additional prompts:* What makes you say so?
21. **Is there supervision/support/mentorship at workplace?** *Additional prompts:* To whom is it targeted? Who implements it? When does this happen? What has been your experiences of supervision/support/mentoring at work? Can you give me an example? (nurses and student nurses)
22. **Does the workplace plan and implement off-the job formal training (including both short and long training opportunities) aimed at improving patient care at work?** *Additional prompts:* Can you give an example? **If you have attended any of these trainings, c**an you comment on your experience? Do you feel you have utilised what you learned? What makes you say so? Are there any specific workplace factors that contributed to this? What are they? (nurses)
23. **What stands out as a factor(s) that determines how nurses are likely (or not) going to utilise that they have learned at their workplace?** *Additional prompts:* Tell me about institutional leadership factors, mentorship factors, other institutional initiatives (which, for who, how, when)? (nurses and student nurses)
24. **Tell me about individual nurse-specific factors that you have observed that determine how likely nurses are to use what they have learned in their workplace** (looking for things like effective communication, teamwork self-awareness, decision making etc----human factors)? (nurses and student nurses)
25. **Did you feel you are confident in communicating with rude, angry or disrespectful patients/colleagues?** *Additional prompts:* Why do you say so? Can you give me an example?
26. **Are there emotionally challenging situations that you find handling at workplace?** *Additional prompts:* Give me an example? Why do you say so? How does this impact on the care you give to your patients?
27. **Do you think there are factors about your other professional colleagues (doctors, nutritionists, laboratory personnel etc) that contribute to how nurses utilize knowledge and skills and competencies acquired during their training?** *Additional prompts:* What are these factors? Why do you say so? (nurses and student nurses)
28. **Do you think the nursing school training theoretical work (including in clinical skills labs) is aligned to what is expected at the workplace?** *Additional prompts:* Can you comment on this as a student nurse, a newly qualified nurse and as an experienced manager/leader?

C: Social Networks Questionnaire

Demographics

- Study ID number
- Job title/cadre/student status (include qualification and level of seniority)
- Unit (maternity, postnatal, neonatal, paediatric)
- Year of birth (or age in years)
- Gender
- Highest qualification (and year)
- Specialist certificates
- Training courses/workshops attended (in the last 5 years)
- Involvement in research
- Academic/teaching/faculty position
- Nomination as a clinical mentor/champion/coach/preceptor
- Length of time working on ward
- Employment elsewhere

Scenarios

Type of support	Scenarios
Instrumental	You need help to perform a routine procedure on a stable neonate. Who do you request help from?
	A neonate requires emergency care. Who do you inform?
Informational	You are not sure of the dose of medication to give to a neonate. Who do you ask?
	A mother has refused a procedure for her baby. Who do you discuss this with?
Emotional	A baby has died on the unit and you are uncomfortable breaking the bad news to the mother/caregiver. Who do you speak to?
	You have had a very busy shift and two neonates died today. You are feeling tired and upset. Who do you talk to about how you are feeling?
	A colleague has been rude to you. Who do you speak to?
Appraisal	You notice that you are unfamiliar with certain core skills necessary in the NBU/maternity/ward. Who do you go to review this support?
	The hospital management has asked staff/students to identify a clinical mentor to support them improve their performance at work. Who would you choose?

List #1 = Roster method – a list of staff working in or supporting the unit

D: Table 1: Detailed Context-Mechanism-Outcome Configurations (CMOCs), with illustrative quotes from interviews

GROUPING	
Shared identity and purpose	**G1: When staff share a similar scope of work (C) they will understand common information needs (M) and will be able to share useful information/ support (O).**
	(Nurse) nurses, (laughs).....I think because we have a lot in common... because most of the time they're giving me reports, am inquiring about duties... am inquiring about commodities, so, most of the time I would find myself more,.... MO, MOIs….. yeah, we talk, but not like frequently. I may talk to him maybe asking, what about this treatment sheet... do we administer this drug now? .... maybe, do I put a cannula, so…. not as often as nurses.
	(Nurse) Well, I think being colleagues and the type of work that we do, it's the same. So, you'll find us most of the times together performing our procedures.
	**G2: When staff or students are considered peers (e.g. same cadre, alike in age, employed at same time, training institution, etc.) (C) they feel comfortable/ more relaxed with one another due to shared understanding and perceived commonality of thinking (M) and will help one another, share new information, correct one another, be concerned for one another's welfare, swap shifts, social enjoyment (O).**
	(Nurse) I will go maybe to an older person like me because we have same thinking.
	(Nurse) But you find there is that feeling, you have a common feeling towards the patient. We're here for the patient.
	(Student Nurse) Yeah, we could… there are those we could discuss with, the same, same guys because it's like we…we are familiar, not even familiar that they are at some point in life, you can relate with them they can relate with you. The kind of life I've gone through; they have gone through.....Yeah, some common life experience that could allow… that could allow others to relate with you and even encourage you and even discuss about my career.
	(Senior nurse) … you can pray together. So in case of such a thing, emotional support, spiritual support, they are those ones that I go, I go to.....
	**G3: When staff are friends (C) they will understand, trust, feel safe enough to be vulnerable, and have pre-existing foundation of reciprocity with one another (M) so will communicate truthfully and frequently, confide in, and help one another, and enjoy being on shift together (O)**
	(Nurse) If you are, if you are my friend, I will definitely open up some things for you, because you are closer to me.
	(Student Nurse) When in the NBU, I made friend, as in, we became good friends with her. So apart from the NBU and hospital stuff, we would talk other things apart from the NBU. So, yeah, I found it like very, as in I confided in her very much in most of the things. Yes
	(Nurse) She's my friend and whenever I'm in .....she will come and find me where I am. Yeah. And she has some stories [laughs]...... Now when you are free, when you have a free time, maybe you can meet in the tea room and start talking.... And you can talk to her and tell her everything.
	(Nurse) typically, they are my friends (laughs).... I value friendship.... career wise, for progression. Coz, I trust them, they will give me good feedback coz they know my qualities, they know how I work.. so, they understand me better, that's why, I opt to seek their advice.
	**G4: When there is fragmentation within a cadre, e.g. by age (C) friction may occur between subgroups (O) due to 'othering' (M).**
	Observations
	**G5: When colleagues are trusted (C) others will go to them for advice (O) as they feel "at home", that they can speak in confidence, and the colleague is interested in their good (M).**
	(Nurse) Yeah, I think it also involves trust and you see people are different and you cannot just tell everyone how you feel, there are specific people that you choose among the circle you have that you can express your feelings. Yeah
	(Nurse) And you feel like you are at home with them. Cause they give you the best advice, they feel that will help you.
	(Nurse) I think when we met, we bonded and when we, we can keep a secret. If I tell them something, they can they can keep a secret, and when they tell me something, I do keep their secret.
	(Nurse) Another thing, I go to somebody who is loyal. Somebody can give you time, listen to you and give her your views.
	**G6: When there is cordiality and respect across cadres (C) free communication is encouraged (M) and teamwork improved (O).**
	(Student Nurse) Now, we have a students' instructor and we also have the team leader. So for the supply stuff, you have just to get the key because she's the one who holds the key to the store. So you have to go talk to her to go pick the clients. At the same time, she's also very receptive, like, yeah, now how she handles students is a bit different from, from other people. So, I would prefer talking to X other than other people...... Politely. You know, you are a student requesting for store key. You know like, it's not something simple. Yeah, so you see, when you go to her, she will treat you like, just like a student; very polite. If the supply is there in the store, she will send you, you go pick it and then you return the key
	(Clinical Officer Intern) As in you talk to them but you can be like more close you find yourself being free....Yeah. Yes you can approach them in terms of work and so on but now the friendly part.
	(Nurse) So you go to the doctor, Let's say, doctor, this patient has this 123, I think with this and think, OK, you can also give your own suggestion as a nurse.

	(Nurse) I just say let's just have a good working relationship, let's just respect one another regardless of your level of education. Because here people we have... there are those who have done diploma, they're those who have a degree, there are those who have a degree and they also have specialization...there are those who have just degree only, without specialization. So regardless of wherever you are in your level of education. And then another thing, don't undermine someone and then if you are corrected, just take it positively.
	**G7: If advice received aligns with existing beliefs, is evidence based, patient-centred, and the person feels that they were listened to (C) advice will carry more weight (O) because it is deemed reliable (M).**
	(Student Nurse) Okay, we, if I receive something like that, I think the best thing I do is just goes by the evidence-based practice.... You know, if you give me some, let's say, literature, you'll just have to tell me where you got the literature.
	(Nurse) yeah. Once we've referred to our nini (whatever), our opinions, whenever, whoever's opinions aligns to the procedure manual, the standard policy.... is the one we shall adopt.
	(Nutrition Intern) ...Especially if it's like evidence based, which is like what we are, it's been grilled into our brains. so, if, if, like say, am giving, am calculating a baby's feed by 150, for me, a maximum is 150, right? And then a colleague comes and goes like, no, no, no, actually a maximum is 180, this is why it's 180, this is where I read it. I wouldn't continue with 150.... as much as I would want to be rigid and go like, no, my answer is correct, if it is, if their way is more, it makes more sense and it's more patient centred and it's, there is evidence that that's what's supposed to go, am just gonna change from my 150 to 180 and, you know,
	**G8: When there is inconsistency of messaging/discourse between different cadres (C) there is lack of clarity of approach (M) preventing effective teamworking on the unit (O).**
	(Nurse) yeah. It happens, because some of the time, you'll find, most times,.. what we almost disagree in, it's about CPAP... a criteria for putting a baby on CPAP....and aaah, you'll find sometimes, let me say, as nurses..we feel like, this baby is not suitable for [CPAP].... but the consultant will insist, this baby to be put on CPAP.....
	(Nutrition Intern) ...... So, at the end of the day, it becomes very, coz the patient now doesn't know who to follow, ...they end up following who they feel, is more accommodating, or who talk s to them in a very friendly manner, yeah. It does affect, a very huge chunk.
	**G9: When there is effective teamwork/ collaboration/ inclusion (C) staff and students will feel valued by colleagues and have a sense of belonging (M) increasing enjoyment at work (O).**
	(Nurse) then the…there is the teamwork in the unit, the… we are very many we are over 80 but we know each other where you meet with them you know, sometimes you… when you are on leave you even miss to come back.
	(Senior Nurse) … So the spirit of teamwork, it's very good. And I think that one is motivating staffs. The way we are also working with the doctors. The doctors we have, they are good doctors, you approach them and give suggestion, you just talk and agree…..
	(Nurse) You know when you come to work you know that you have a big team which is prepared and ready for anything It boosts your morale... Unlike when you're coming to work you see your people maybe they are not ready, there are those late comers, people have their own social issues. So it doesn't add up.
	(Student Nurse) I like someone. When I am working, I like really working as a team, I like working as a team. We, are there for each other. If you are able to help.
	**G10: When staff respect colleagues of other cadres and mothers (C) inclusive inputs will be valued (M) and contribute to decision making (O).**
	(Resident) There so many that they [nurses] know actually they're better than some of us and then you tell them, "I have done this ABCD." Or for example umekwama [you are stuck] somewhere, let's say you get stuck somewhere, and then like she's, she's done this several times, she will tell you 'I had done this way, severally." Yes, and she will then say, "Yes, you're doing it correctly."
	(Student Nurse) Because I at least my opinion is accepted. When we are discussing the care of a patient, whatever I say has some weight.
	(Resident) The nurses, yeah, we also interact a lot with them. They have a lot of knowledge about how things are done in the NBU, they are very familiar with the protocols, so they can very easily guide us on what the protocol, they will assist us, before doing a procedure, they know what is needed to prepare for that procedure. So, they're very good at that. Yeah, that's it. They also give us support. I mean, some of the intensive care nurses are also good at intubation. So if you're stuck at night, they will, sometimes they will even intubate for you.
	**G11: When staff feel that they share a passion for work/ patient care with their colleagues (C) mutual support and teamwork (M) will lead to improved staff wellbeing and patient care (O).**
	(Nurse) Their character, their principal... Hardworking, someone who's focused. As nurses, we advocate for the patients, so, someone who advocates for the patient.
	(Nurse) Communication, when you have a common goal then communication will be much easier and like having feedback also from…if there is an issue that you are discussing we need feedback…
	**G12: When staff feel safe with their colleagues at work (C) there is a sense of freedom (M) and wellbeing improves (O).**
	(Nurse) You'll feel more comfortable. When you are around them.. as in you feel so comfortable and you feel so comfortable, feel so happy. When they are there, let's say that. What can I say? Let us just say that they will help you whenever you need help.
	(Nurse) And they are people that, maybe if they have some cake they can share with you. Yes, they can share with you. .. Yes. Or a person who is always having advices, they can advise you in a better way. They see you doing mistakes, they correct you in a lovely manner. So you feel protected when you are with them. You feel safe. .. And also they support you. When you're overwhelmed, you just see somebody's coming, holding your hands, instead of just relaxing and you are stretching this side.
	(Nurse) I'm safe.... Yes, we have those people, when you are doing them, you're safe, cause if you are stuck somewhere they're there to help.....They are helpful. You can consult and they help. If there is a crisis, they're the ones to run to. They, they only, they take the action immediately. Yeah... They act quickly... Mm. Any other apart from, they're helpful. You can consult them freely. When

	in a crisis, they're very quick. ... I just like how they work. Some are perfectionist, you see when in when they're in the room, taking care of the babies, you just see everything is smooth. ....And they're responsible for everything that is happening.
	(Consultant) When I know that I can be the best and the person who I want to be, it definitely improves my wellbeing. Yes. Because it almost becomes like another home, that I know I am coming to where I can talk to people, I can, discuss what affects me, and, am not alone….
	**G13: Where there is friction/ conflict between members of different cadres (C) staff may feel stressed at the start of the shift (O) because they anticipate difficult communication (M).**
	(Nurse) If it’s someone difficult like let’s say like that scenario of the MO and you, you know when you, like now when you go on overriding his decisions and going to the consultant, you see it, it brings... friction between you and even if you meet to work, you’re kind of not comfortable with each other because of the friction that it was brought between you two.
	(Student Nurse) There is this group they are called registrars, I do not know whether they have worked prior or maybe they are MOHS, totally disrespectful people. Especially now those who are here, leave needles everywhere, and they leave blood without cleaning after they spilled it, I feel they are a bit unprofessional. ...... every nurse who comes there is complaining
	(Nurse) I will say they are more of individual because there is this perceived silent rivalry between the nurses and the medical officers because the nurses feel they have the experience and medical officers feel they have the knowledge.
	(Nurse) So there are some people who take to…to delay by time like no, let's do this when you know it's not supposed to be… …like that way. So you keep on arguing arguing for the sake of the patient and at the end of it you feel like …what are we doing here?
	**G14: When multidisciplinary colleagues are allocated to sub-units together (C) they will work as a mini-team/ dyad (M) conducting mini-rounds and delivering care to patients (O).**
	(Nurse) Yeah, even the ward rounds. For example, if they decide that these are the IV fluids for today or this is what the babies are supposed to feed on. We are always there. OK. I would say in the new neonatal we always attend ward rounds so that we get to know what has been said. And the plan of care we discuss together.
	(Nurse) Yes, I normally attend when I'm available. I normally attend my own rounds. Actually, I can give the nurse’s perspective on how the patient is progressing, what needs to be adjusted and one needs to be added on the treatment. Yeah, okay.
	(Resident) Actually, it’s okay. The good thing about NBU is that there is a pathway.... So I think everything from the nurses, to the doctors, to the seniors, it's organized, okay.... ……. So if you're there, you know what is happening here. If I'm here, I'm doing the correct thing on the side, the nurses, the doctors, the everyone else.
	**G15: When multi-disciplinary colleagues are allocated to the same patients and work together as a mini-team (C), a shared purpose and sense of belonging (M) enhances communication (O).**
	(Senior Nurse) Most of the time I take the fellows and the residents, all of them, they are the doctors. We are working together, and we are working as a team. So for the part of the doctor, we are always working, since we are working as a team, we work together in terms of aseptic technique on our patients. So we have to keep on reminding them because most of the time you get, when they are doing procedures, they are not able to, they remember, they know, but they can't really remember to use the aseptic part of it. So that is the time we meet and talk. And sometimes during the management of patients, especially for patients who are due for discharge, we talk and agree whether the patients can be discharged or not. Sometimes when our mothers have issues and they would like to know more about their babies, you find that, that is the time I talk to the mothers and I incorporate the doctors also to talk to the mothers. So I talk to the doctors frequently also.
	(Nurse) Like the doctors, the doctors, well, I speak to them like for example, let's say I'm in a room, the doctor who is in that room, I’ll constantly speak to the doctor because I’ll need, because you see we are… Yeah, we are collaborating on the care of the babies. So if for example you, if I have IV lines, if I have a very sick baby, you see we would constantly talk with the doctor then I’ll see the plans that he has for the babies on that particular day. Yeah.
	(Resident) So if you're there, you know what is happening here. If I'm here, I'm doing the correct thing on the side, the nurses, the doctors, the everyone else. So it's like if you're doing a certain room, you're responsible for that room, we take care of everything that is there. If you can't do, what do you call, if you can’t, if you don't find it quite okay, then you call for help. The seniors are always there, they always come and review the patient, we discuss the patient with them, yes.
	**G16: When different cadres are used to working together in a team (C) they will assist one another proactively and accept assistance, pooling knowledge, expertise, and differences (O) as they recognise that mutual assistance and support is for the common goal of patient benefit (M).**
	(Resident) We all in the unit, we all would call as a package Yes. So if you want certain things to be done, and then you ask the nurse that is with you on that, with you in that particular room...... There so many that they know actually they're better than some of us …..let’s say you get stuck somewhere, and then like she’s, she’s done this several times……
	(Resident) The nurses, yeah, we also interact a lot with them. They have a lot of knowledge about how things are done in the NBU, they are very familiar with the protocols, so they can very easily guide us on what the protocol, they will assist us, before doing a procedure, they know what is needed to prepare for that procedure. So, they're very good at that. Yeah, that's it. They also give us support. I mean, some of the intensive care nurses are also good at intubation. So if you're stuck at night, they will, sometimes they will even intubate for you.
	(Medical Officer) I would say it's everyone. Everyone... we influence each other in different things. Yeah... each other it’s not like a particular person we all have… today somebody has like something to add on to what we are doing tomorrow It's a different team. ... Like everyone helps and influences each other. Yeah..... I think it's good because say sometimes if I'm overwhelmed a nutritionist will tell me let me help you put this this this. ....Sometimes if the nutritionist are overwhelmed maybe they'll be like could you help us ABC. I think its team teamwork gives us good outcomes.

	**G17: When staff see their individual actions as contributing to collective action (C) they will be motivated in their own tasks and value the inputs of others (M) leading to improved satisfaction and care delivery (O).**
	(Nutrition Intern) So it came up with solutions and actually it did help the baby. Yeah.
	(Nurse) Okay as a, when you are a team leader one thing you when support you have to give to the staff if you don't then you are failing, ensure the consumables are within reach. There is really… it is really very bad when a…a nurse who is in NICU with ventilated very sick babies having to come for things in the store.
	(Nurse) Yeah, I gain knowledge because, especially, handover is a routine, but...it's not... You don't do it casually. You have to be in detail, because this person you are leaving the care to of the patient has to know what you've done and what... What you've done to the patient and what is remaining and what is expected. So you have to give a detailed report for the patient. So that's the handover part of it. So, you do it thoroughly, not shallow. It helps because once you get the information is complete, this patient will get the correct management.
	**G18: When different cadres of staff feel they are valued members of a team (C) patient care will benefit from more holistic inputs (O) as staff will be encouraged to express their different professional lenses/ selves (M).**
	(Nutritionist) I can say collaboration of the staffs some admire, because you see, in other areas, some nutritionists even they are not even acknowledged that they are there; but here, they can't do anything if a nutritionist is not there because they want your view concerning a baby. So, all of us, the collaboration is so good.
	(Resident) We all in the unit, we all would call as a package Yes. So if you want certain things to be done, and then you ask the nurse that is with you on that.... There so many that they know actually they're better than some of us and then you tell them, "I have done this ABCD." Or for example umekwama [you are stuck] somewhere, let's say you get stuck somewhere, and then like she's, she's done this several times, she will tell you 'I had done this way, severally." Yes, and she will then say, "Yes, you're doing it correctly."
	(Nurse) Of course the, the interest of the patient comes first, like for example the doctor is insisting on collecting the samples for bilirubin first before starting the patient on phototherapy. I would choose to start the patient on phototherapy first, as I, I wait for the bilirubin's, because at the end of the day, it's the patient who is going to benefit. Sometimes the, the physical symptoms, might lie to your eyes.
	**G19: When a doctor does not communicate appropriately when providing patient care (C) the nurse will feel undermined (O) because the doctor has displayed a lack of awareness and respect for her professionalism/ competence (M)**
	(Student Nurse) But you see here most of our doctors are a bit reluctant to be helped. You see someone standing with something that you can do in seconds. When you tell them that want to help them it is like you undermining them. So, you give them a face and let them try and try and when you see, they are not able to do it when you step in.
	(Nurse) ..are we having a barrier? Mmmh, I think mostly it will go to, communication concerning maybe care of a neonate, care of a baby, from maybe the medical team to the nursing team.... I think sometimes, they, they give orders, with no reasons. You know, if you tell me, do this, put this baby on CPAP, I think you should tell me with reasons,.. not just, 'put this baby on'….., and its final. You see?.. so, I think, I think, communication between maybe, medical team and nursing team. Mostly that's where barrier is. I think so.
	(Nurse) Maybe now you know something and you are sure you know, but here comes your senior, like the MOs and the whatever, the consultants, you try to tell them these things is supposed to be like but they think that they know much better and they don't believe that you know. So, they can say, "No, that thing is done like this and this." So, maybe you're not given chance to apply. So, that's the thing.
	**G20: When subgroups of staff work in parallel with little collaboration (C) staff are unlikely to take instruction or recognise inputs from an outsider, instead disregard or 'look busy' (M) leading to inaction, frustration, blame, and worsening of collaboration/ feeling of being let down by other subgroup (O).**
	(Resident) ….. the nurse that was assigned to that particular admission room would not help, so you look for the gel, the lubricating gel and I had to personally go to the ICU two in the morning to look for it because that was the only thing missing and like no one was helping, so that made it a bit difficult. Of course, it made me hesitant after that, to, you know, even when working for them, I would just, I would not ask for….. Yeah, they would not assist.... Yeah, it's not everyone, but there will be one or two. So when I knew I was working with them, I would not ask I, if I could get something, I would get it myself, just to reduce the stress....
	(Consultant) It is frustrating…because the cadre lines are very clearly drawn in our public facilities, so I have to take it with caution. It is very difficult for me to discipline a nurse regardless of, you know, of the occurrence. I have to go through their ranks.
	(Nurse) That one I think it's based many times with the issue… its just some not all, but when you reason together you you come to an agreement. It's about like if I'm communicating an information like as a nurse to an MO he would ask like you're not supposed to talk to me directly. Is there an MO intern on the ground? Are you seeing....
	**G21: When staff cannot communicate effectively to come to a unified plan for the patient (C) senior staff instruction will usually be followed (O) due to authority (M).**
	(Nurse) When the, (consultant) came... The consultant. So, she was like, "I'm being told that you having difficulty to put this baby on CPAP." So, I didn't want to argue, so I just told her, "I don't have any reservation, but if you think the CPAP will help with the baby, you can go ahead and put the baby on CPAP."
	(Nurse) Yeah, doesn't want to come. He's just ordering the MOI who doesn't know anything.

	**G22: When staff and students are engaged to participate in activities on the unit (C) they will feel valued, encouraged and a sense of belonging (M) and will have confidence to make contributions (O).**
	(Student Nurse) You know if there is some procedures being done and someone is involved, you can easily gain them..... I think having, having mentors.
	(Nurse) there Is this time, there is a COI giving information to a doctor, but she wasn't really sure of the baby....so, I really helped give them information about the baby.
	(Nurse) Also we normally have CMEs. The CMEs we have them where we are in different target groups. Every group has its own target, which is supposed to be done every financial year. So if you are in a particular target group, there are those things we normally sensitize people on what we are dealing with and we do audits. Like if maybe you are doing an audit on vital signs, you have sensitized people on the importance of taking vital signs and recording, you do a follow up. You do an audit, people doing vital signs the way it is required, the way it is being recorded, are people taking action after maybe a recorded vital sign which is beyond the normal levels. Yeah, so we do CMEs.
	**G23: When there is collective habit (C) resistance and established norms (M) will mitigate efforts to implement change (O).**
	(Nurse) Maybe if people are used to doing things in a certain way.
	(Nurse) Relevance. Somebody doesn't find it's relevant, there's a sort of traditions and cultures. People are used to doing things differently. And also they are resistant to change So you find somebody is used to doing something in a particular area and doesn't want to change the current practice
	(Student Nurse) Mostly the students change but correcting a staff is difficult. Because this is maybe a norm here and you cannot change a norm.
	**G24: When staff do not agree a unified plan (C) conflict and confusion will result (M) with staff (and mothers) weighing advice based on personal characteristics of individuals (e.g. seniority, friendliness) (O).**
	(Nurse) So, in such an example, you, you really torn in between and you will not even know what to do now. So, when that happens, then now it goes back to the level also of the experience of this consultant. So, if I have a consultant that you've been with for 10 years, but with a lot of humility, I have another one you've been with for maybe a year, I'll consider the senior consultant.
	(Resident) No, the consultants will not agree even if they're in one room, they will not be able to agree on the way forward, because it depends with their clinical judgment. So, you, it's contradicting because the person last week that managed patients in this room did this because this particular person was doing the round. The next week, they'll be told, do this..... You do whatever the consultant tells you, the one that you are with.
	**G25: When communication is not intentionally developed between cadres (C) staff of different cadres may default to giving task-based directives (O) as collaborative communication is absent (M).**
	(Nutrition Intern) ..... And then there are those who come into the ward, and everyone is like, haki ni nani ndio ako ward round (oh no, it's so doing the ward round),….. so everyone is just like, you make sure your things are correct, the only time you talk is when you're asked a question! Beyond that, be in the ward round, (claps), be seen, and not heard, period.
	(Nurse)... so, she was doing her normal rounds, when she came to that baby, she started shouting like, you guys are here, a baby is not breathing,...so, maybe one of you is feeding, another one is on medication, one is changing linens for babies...so, there are times when you can't see everything.... you know, so,.....everyone, the whole, everyone
	(Consultant) I think it is culture. I sometimes feel we don't engage them enough in the ward-round, to feel useful.... Many times, the nurse is the silent bystander… and within 15 mins they (nurses) disappear on to do other things where they feel more visible. …..

**Being careful towards colleagues and patients**	**G26: When staff are willing to listen, take time to understand, show patience (C) others will go to them for advice (O) as they feel comfortable to express themselves, feel understood and know they will not be dismissed/ judged (M).**
	(Student Nurse) Yes, there reason why I had to go to X, you know doctors are, they seem to be so busy especially when, when they're doing the procedures. So, even taking that time to explain something back to you becomes a problem. So, with X, he's, he's humble, he has got the time to explain... He gives us the time to, to ask questions and then he responds. Yes. So, there's that environment for learning with X as compared to other people.
	(Nurse) You look at somebody's you know, you, when you interact with people, you know, you know sometimes their character, even if it's not their whole person. But you know somebody who listens, somebody who encourages, somebody who maybe talks when you talk to them, you feel like they really understand you.
	(Nurse) ....and they are attentive, like, they'll stop what they're doing, and they'll listen to you, then…yeah.
	**G27: When staff are considered religious or trained in counselling (C) they feel they can be true without judgement, and will receive comfort and advice (M) so will go to them for emotional support (O).**
	(Nurse) Yeah, I would say a factor that can make me approach someone is their relationship with God. You see, not all. Not everyone is a believer. And for me to approach you, I must see something that attracts me to you, and a very important factor is the relationship you have with God. So, I think that is a factor that I will also consider before I share my emotions …..
	(Nurse) I think for that, I'll open up to a religious person, which is X... since, for them they won't take sides, they are not biased.. and for them they'll opt for, they'll comfort you... I can also go to a nurse or a doctor.. coz of, I think they've learnt the bereavement process.. yeah, so, cause they'll know how to take care of me. They'll know what to say, what not to say.
	**G28: When staff show humility (C) they will respond positively to correction and others will offer them advice (O) because they recognise the importance of continuous learning (M).**
	(Student Nurse) A nurse. Okay. I did offer an information, I guess it was, it, it was once sometimes ago, about the medication of the drugs.. She received well and she, she said she'll go and refer and later the next day, he told me, actually, you are right... It had an impact on her.
	(Nurse) Oh, yes. Not? Yeah, not all, the particular ones who are ready to listen and the ones who are, the ones who are, who you are friendly with.....Or ready to, ready to be taught something… To learn, yeah.
	(Nurse) There is. Like now there is a time the MO can hold her, the patient needs a CPAP, you can just go there and access with her or him and you can change everything and you can listen, she can listen and change. Or concerning medication, cannot one tell you, tell her, "I see this one is supposed to be one, two, three." She can change.
	**G29: When staff show humility (C) others will ask them for advice (O) because they show integrity (M).**
	(Nurse) ... and if, let's say they are not able to do it, they'll seek somebody else to help them, let's say.... like inserting an IV line, if it's troublesome, they'll ask someone else, they won't keep on .... putting the child through pain.
	(Nurse) and emotions? Now, like a hard day or what, or what?.... Most of the time, I'll find a colleague who is close to me.. that is one. Like someone, who will not, who will not favour any team.. if am explaining like so and so did one or two to me,.. he will be like, 'ooh, he did that? but, you see',.. and there is someone who will tell them the truth... it's not someone who will just side by you because you're a friend, he will just say 'you see, even you, you were at the wrong side' you see?.. like, like X, will tell you the blank like, 'you know, you did wrong, that's why you did so and so'.....
	(Nurse) ...eeer, first things first, she'll communicate, when she's sure of something, she'll tell you. If she's not sure, will confirm from a book...
	**G30: When an individual treats mothers with respect (C) others will go to them for advice (O) as they model esteemed values (M).**
	(Nurse) Yes. You know, there are those people who are just gifted in communication. They just talk and it flows out nicely, and makes things easier, even for a client.
	(Nurse) ... mmmh, and the way they relate, maybe if the mother is there, ...their relationship with the mother and the child.
	(Student Nurse) And even the way they could maybe handle the mothers could…could help you to learn how… because here it's only… not only about the neonates,....it's about even the mothers who come here, others have issues, so the way they handled them with… could even cause you even to desire to be with them
	**G31: When an individual has a positive attitude to work, e.g. joyful, not complaining, not gossiping or talking negatively about people (C) others will feel good (M) and want to work with them (O).**
	(Nurse) They're jovial, they're helpful, positive, they're just a vibe
	(Nurse) These are, okay, they're positive in every aspect. Not a time, you'll, you, you'll hear them talking negatively, or commenting negatively about people, about situation. About the nature of allocation, that person would work anywhere
	(Nurse) So they're willing to help, they are like joyful. They are lively. They, you feel like you are working as a team

	(Student Nurse) There are those people who are very positive about what they are doing... Very positive, whether they are being watched or not, they're doing the right thing. .. Doing the right thing, handling those babies well, when the mothers come others have issues they will handle those mothers well, that in fact touched me because there are those people unless you are there watching over them they could not do the right thing. There are those who work because they want to earn but there are those people who work because it's a call. ... So there are people I watched and said, surely I would like to work with such people because they have that heart.. for… okay whatever they're doing they're not doing it for the sake of earning, they're doing it because it is from inside their hearts....
	(Nutrition Intern) Positive colleagues. .. So it makes you work better because you are motivated as well.
	**G32: When a member of staff is committed, hardworking, resourceful, takes responsibility, punctual, reliable and helps other staff (C) others will esteem them as patient-centred, selfless and a good team worker (M) and will feel supported by them, and want to work with them because they will reduce overwhelm at work (O).**
	(Student Nurse) That man, can leave what he's doing, to come and assist you on what maybe you've asked a question about or maybe you don't know how to do something. Or maybe you're fixing an NG, and then you've gotten to a point you're stranded, he will leave what he's doing to come and sort you out.
	(Senior Nurse) So first when I'm considering, or people I'm comfortable with, they are people who are always available when they are on duty. You can get them in, if they are at admission, you get that person there. Then another thing is somebody who is able to sort out issues, not only all issues in the nursery you are referred to. Mama asking anything, go to the office, somebody who is able to take charge without even being delegated. Able to handle mothers able to handle issues on the ground.
	(Nutritionist) They're easy to approach.. And then they are friendly, and they tend to share the same values as me. So we can reason on the same level...Values are like maybe someone who's God fearing. God fearing, honest. Honest in the sense that, you know, like sometimes we have people who just want to shortcut, who don't do their work well. Integrity in short
	(Resident) Those that you can work with. The person you see you know what this person is reliable, reliability. One, if this person you give them a certain task, they don't leave that task unattended, they make sure that they do it up to the latter, yes. So mostly I like to work with people who are hardworking, people who are task oriented, people who are like, “You know what? We’re going in and but you have to finish than we don't leave anyone behind.” Even if they finish on their side, they will come and assist the other person, yes.
	(Nurse) Very much available, very much committed to the work. For X, she knows everything by the way, she can help you in anything, you tell her to do this, she can do. She also is hardworking. She motivates, she does the right thing. You know there are some people who have shortcuts in doing the job, so, she likes straight.
	**G33: When staff see a colleague reliably completing tasks, arriving early etc. (C) they know their colleague will complete the work (M) so may take advantage and negatively adapt their own behaviour, e.g. arriving late (O).**
	(Nurse) ...Yes, especially the machines we are using, if, if you instruct somebody to prepare a CPAP and they will tell you, “I don't know what parts go where.” So even if you explain it 10 times, they will always tell you they don't know, so you avoid that.
	(Nurse) .. sometimes heh, when some people are working heh, some people will take advantage because they know things will be done... yeah, even in reporting, they know. when they know someone, so and so may come early. Someone will come late because they know so and so will be there early. You know that kind of a thing.
	(Nurse) So I don't know if it's rudeness or maybe take advantage of you because you’re doing the work.. So sometimes you just prefer to be, or you come early, she comes late and she’s not doing her work..... Yeah. Then she’s the one going to leave early and you’re the one who have been there earlier than her or him
	**G34: When staff are ready to help (C) they will be sought for support (O) as others will know that they will receive what they need quickly (M).**
	(Medical Officer Intern) ...they are present, and they’ll come really fast and are willing to help.
	(Student Nurse) Like let's say, if there is a patient with an issue, like a baby, and then you would tell, her, a certain patient doesn't have a line, or something, please review, she was easy to. she would, like, fast.
	(Nutritionist) I think, even if, when, when you go to them, asking for assistance, for making some inquiries, they, they give time to…. they address it at quickly... yeah. You are…. there is some support you need from them; they are ready to offer.
	**G35: If a staff member can explain the rationale behind advice given (C) it will be deemed credible (M) and carry more weight (O).**
	(Student EMT) About them they were also during break time or tea breaks... during maybe we are documenting they will tell you why, they educate they tell you why we do this, this is how you do it and explain to you why we do this way
	(Medical Officer) Okay, there are those few doctors I can… I cannot remember their names, but few around three doctors who were really there to teach you, to guide you, to help you understand what they are doing.
	(Nurse) So you tend to go back, sit back and start looking is her reasons weighing mine or mine are weighing his? So you look back on that scenario then when you start listening to the person, you leave yours and see that the other person’s it’s making a lot of sense. So you back off then you let it go, you pave the way for it.
	**G36: When a colleague shouts (C) staff/ students will feel intimidated/ ashamed/ humiliated/ fear (M) and will not approach them again, will lose self-confidence and will opt for workarounds (O).**

	(Resident) Maybe you want to ask a person for help, a person maybe starts scolding you, the person maybe. You know there is no that approachable face kind of maybe just like, let me just veer away let me just, you get this one there other so many people let me do it..... Nope, is just a personality, the character of the person, so you can't change the character. If the person's character is like that, you just leave. But has nothing to do with the age or, yes.
	(Student Nurse) When you are being shouted at.... Yes, you'll have low self-esteem and you'll find that nobody even maybe believe that you can do so maybe you are doing something and someone, that's not done that's not done that way, so you will just develop the fear and you will just like doing things when somebody is not watching you.
	(Nurse) They are not patient, and then they shout. I don't like people shouting. When you go to somebody, maybe you are inquiring something instead of solving that thing in a calm manner, somebody shouts. ... And then when maybe you go to somebody to inquire something and then somebody puts you down It's like you are nothing you don't know. Why don't you know this? It's like you would have known this before why? Some people don't know that learning is continuous.
	(Student Nurse) Harsh.... uh Like how somebody communicates to you….depending on…okay there is… I can come and ask you maybe what… maybe like something simple or I was doing something …instead of coming and telling me you do it like this you are doing it the wrong way, when you start shouting at me...I won't come to you next time. Yeah.
	**G37: If someone is blaming, dismissive, domineering, condescending, bullying (C) others will feel uncomfortable approaching them (M) and will not go to them for advice or want to be on shift with them (O).**
	(Student Nurse) I don't like people with condescending demeanours.... it spoils my day. like, you know, it just spoils my day. Like after that run in with her, I just, my psyche just went from here to, pshhh..... actually, I didn't even touch another patient that day... the whole shift, I was just seated.
	(Nurse) ...they don't have good communication skills, you know, you can ask me, 'please help me' or you can just call someone out of, 'we(you) student!' do this and this', you get?
	(Student Nurse) Of course, it will, because they are rude to you and I will be like, I have asked you a question, probably I am a student, but I am also a qualified nurse so... ……. But there are times in a way you ask someone something and they are like ...you do not know how to deal with that. As much as I would like to undermine you. Or to judge you, maybe... Sometimes. Maybe judging like they do not understand. It is something like basically the supposed to understand, which is assuming that you are supposed to know, and you do not know.
	(Clinical Officer Intern) You know, because those are the guys whom we are within the ground. So, any challenge you get you can discuss with them easily other than calling an MO, maybe a consultant who maybe is not available and maybe when you call her, he may a perceive you in another way....Okay, you understand the kind of the knowledge they have, when you ask simple things is like they will see you don't know, maybe they had you one extra, an extra week for you to study.
	**G38: When a sub-group is blamed collectively/ there is an air of blame (C) the subgroup will protect one another, feel aggrieved and defensive (M) leading to othering/ avoidance/ incivility, diversion of blame to another subgroup, cover-ups, and no change (O).**
	(Student Nurse) she doesn't want to listen. Like, me personally, I had a run in with her...(laughs) like she's blaming for something that somebody did not do over the weekend. I was not there over the weekend. Am trying to explain that I was not here over the weekend.. why are you not giving me time to explain? Then she goes like, 'you know, this is why I don't like students, they are just many here and they are doing nothing'... she was just adamant, 'no, no, no. it is you students, sijui (I don't know), it's your fault, you're messing up everything, nini nini(what and what)!' ..... I was like no. I can't deal with you. (laughs).
	(Nurse) with these our consultants, if there is a thing which is not done, it's only nurses who don't do, (laughs). ....it's only nurses who don't do, who don't do their work. And then you're helping their students to learn, you're even teaching those MOs how to prescribe drugs (clicks)... Anyway…..
	(Student Nurse) … whatever she spoke about students I could not even imagine... so what she said is, these ..students are like mashetani [demons/devils] … In fact, when I tried to talk later on, she could not even listen to me but there are those who listened and one of them called me and said don't… don't worry because you know, she generalised us ... You see; one makes a mistake you generalise all of those people it… it's a challenge.
	**G39: If a member of staff corrects colleagues in front of mothers (C) others will not to work with them (O) as it damages the confidence of mothers and makes colleagues feel bad (M).**
	(Nurse)..mmmmh, let me say, I also don't like someone who will correct you in front of other patients... sometimes it happens. Like, you find someone, you're with someone at job, and eeh, he or she is like, 'don't do that to that baby', and is in front of all mothers, you know you feel bad... so, correction, the manner in which people correct others.
	(Nurse) I don't want to communicate in such a way that the patient feels like whatever the other colleague was doing is wrong....
	**G40: If a colleague's behaviour makes staff/ student feel like they are bothering them, e.g. not listening, harsh one-word answer, body language, etc. (C) they will avoid that person (O) as they fear asking (M).**
	(Nurse) There are some who will like you'll, when you ask for help and you observe them, you feel as if they, you are disturbing them. So you just decide, let me not ask this one, let me ask so and so.
	(Student Nurse) it's just those, people who were degrading and also they are like you are bothering them, when you ask questions
	(Nurse) Yeah. Yeah. It's like the ones, okay. Or you just get a one worded answer, and even the tone of voice just tells you, they don't want to be asked questions, so, you get yourself busy
	**G41: When a colleague is not discreet/ shares conversations/ gossips (C) others will not trust them (M) and will not share with them (O).**

	(Nurse) It's very tricky because you know at some point, I cannot say I can go to so and so sometimes you have identified one person you share your feelings, your problems too, but that person again will go and start spreading telling other people.
	(Nurse) Others are, other are very talkative, you can't even share anything with them; they will shout.
	(Nurse) There are these who are, okay, there are these who are, they'll disclose your problems to other people….
	**G42: If a colleague uses sexualised/ inappropriate talk, particularly if behaviour not challenged (C) this will lead to avoidance and development of coping mechanisms by individuals (O) due to harm/ injury (M).**
	(Nurse) I think for him, you need to be, you don't need to take his jokes, he jokes a lot....so, you don't need to take his jokes seriously... yeah, for girls, but for guys...for us we're just okay,
	(Health worker) .... (sighs), arrogance, yeah, male chauvinism, probably because I didn't like him,...
	(Health worker) .... I got to grow, it's not really growing thick skin, that's just basically tolerating him. That's when it became easier for me to come to work and just go like, hah.... wherever, am just here for my patients.
	**G43: When staff are dismissive of, or display incivility towards mothers (C) colleagues will not feel comfortable approaching them for advice (O) as they do not agree with their bad attitude (M).**
	(Nutrition Intern) ... if I see, eer, a colleague, a mum brings up a question, a concern, and they are very quick to dismiss.. to dismiss the mum, anything, even if it's something simple as, " mtoto wangu mbona…?" (why has my child…?) you know, either amelala (slept) this side and not the other side, and they are very quick to dismiss the mum, I prefer not to.. not to engage with them.
	(Student Nurse) The way they handle those mothers; a mother comes maybe ...you know these mothers most of them are emotional …because of the…the whatever their…their babies are going through and they cannot even listen to them or she wants to ask something to understand what's going on… …with the baby, they could not even take time to explain to the mother, they just tell the mother just go out I've told you go out... wait there..... They dismiss the mothers. But they…they are of rare cases, you can say rare cases not always.
	(Senior Nurse) …they are those ones with just this attitude, when they see mothers, it's like mothers asking them anything is like a crime [laugh], they, whatever they answer those mothers, even the mothers themselves just report and say, Aih, when this one is on duty, we are in trouble. So they are those ones, I, I don't want to put to them that their attitude is bad. But their relationship, the relationship with the workmates, relationship with our clients is not really good. So maybe they have an attitude or they have issues that they've not opened up. I don't know. But such a people I'll really feel uncomfortable.
	(Nutritionist) Maybe they are shouting at a mother
	**G44: When staff complain a lot and have a negative attitude (C) colleagues will feel emotionally drained (M) and will not want to work with them (O).**
	(Nurse) ... People who complain a lot. People who want to be treated maybe, to be given first, how do I describe it?... They want to be put first, they want to, if they want assistance, it's them first. Even if yours is ...
	(Nurse) Those who just continue complaining of others. ... Those who don't embrace teamwork.
	(Nurse) Yes, there are people who complain who make life so difficult, even something they can get for themselves they do not want to get...And if you send them they keep on asking you what is the work of the team leader I need this things there are people who can get on their neck the whole day including the small thing they make that something to a big thing. They keep on complaining a lot....They do not want to be sent, they do not want to contribute...they criticize want to be done for everything, they criticize you, they criticize the place, they are not happy with anything.. That is negative attitude actually and it is not new it happens everywhere.
	**G45: When a member of staff/ student is felt to put others down/ be condescending to those who asks questions (C) others will not ask them (O) as they do not wish to be made to feel stupid (M).**
	(Nurse) .. mmh, maybe you ask a question, and somebody says eeer, 'you should read about it'..
	(Student Nurse) Yeah, there's some, there's some, there's some who are, they're seeing some questions as being stupid or what? I don't know if it was something, of ego or something or just a bad attitude of the day or something. Yeah. There are some people who you could not go and approach directly
	(Student Nurse) ako na kiburi sana, sana (he has so, so much pride)... he's knowledgeable, yes, but he has this negativity on him, (clicks), like he looks down upon you...you as students, yes.. yes, I don't think there's any student who likes him... he's so full of himself. So full of himself.
	**G46: When staff display a proud attitude (C) colleagues will be wary of them (O) because they will not admit if they are wrong and respond badly to correction (M).**
	(Student Nurse) Another personality I don't want to be on duty with is the one who doesn't want to receive from other people. That is what I mean, we can call it, they're not dynamic. They're not flexible. Yes, maybe you try a procedure, it fails, or you bring up an idea, but she doesn't want to buy it easily.
	(Student Nurse) Maybe someone who's not sure about the answer they are giving me... Maybe they lack the knowledge about that particular question you are asking them, but because they don't want to show you, they don't know, they just tell you. So when you consult someone else, you find the answer that they gave you was wrong.
	(Nurse) aaah, she is too much of herself. That I can say. As in, you can't, you can't share even ideas, as in, she's know it all.... yeah. She even can go to an extent of telling you that, 'I've been in this service for like, aah, since X? So, you're barely…. employed recently, you lack experience to tell me anything. Yeah.

	(Nurse) Maybe now you know something and you are sure you know, but here comes your senior, like the MOs and the whatever, the consultants, you try to tell them these things is supposed to be like but they think that they know much better and they don't believe that you know. So, they can say, "No, that thing is done like this and this." So, maybe you're not given chance to apply. So, that's the thing.
	**G47: If a colleague greets advice with anger/ defensiveness (C) others will cease from offering advice (O) as futile (M).**
	(Nurse) They don't want correction. Sometimes even though you, you, you, you are going to them in a, in a, in a good way, they're just explosive out of, out of control.
	(Student Nurse) Colleagues? Yeah. Some of them, maybe we give them advice that is kind. Okay. It's kind of affecting their personality and their attitude, most of them replied negatively. Like, okay, why are you walking into my line? Or What's the reason to this? I know what I'm doing here. Sometimes there's negative and both positive, but most of it is positive.
	(Nurse) Yeah, the person is because there's someone you can tell and get the person angry but there's someone else who'll just take it positive, the person won't take it negatively.
	**G48: When approachable staff are physically present, e.g. on same shift (C) will be approached (O) for quick/ immediate answer (M).**
	(Nurse) Those ones I talk to them frequently, because most of the time I go to the bedside, and that is where I meet them and we deliberate on patient management, on documentation, on how better or they are feeling on the bedside
	(Nurse) Sometimes, if a baby changes condition, you consult your next neighbour. Also, you miss something like gloves, maybe when you are a bit committed, very busy, you can send your neighbour who is next to you.
	(Nurse) Of course, the nurses, my colleagues. Because most of the time the other cadres are not there throughout the shifts. Availability.
	**G49: When staff are working with a less intense patient group/ sub-unit (C) colleagues will assume they are less busy (M) and will preferentially approach them for support if required (O).**
	(Resident) By doing so, you can call anyone again that is not that busy, and then tell them, "I need this ABCD to be done while I'm doing fixing this ABCD for this patient, at this certain patient." Yes. So that's why we call upon whoever is available. If it's emergency we call for, shout for help and intervene, the other person does ABCD when you're doing this ABCD.
	(Nurse) Sometimes it's the workload they have. You cannot go to NICU one to ask for someone to assist you, and there's someone in the lighter room who can do it.
	(Nurse) Another one, I'll choose the one who is not so busy. If I see you are having light work. I request the person to assist me if possible.
	**G50: When staff are perceived to be busy (C) colleagues will seek support elsewhere (O) as they do not want to disturb (M).**
	(Nurse) aah, basically, we never used to interact that much... coz they had a lot of workload.
	(Nurse) Also, you see you also have to look at the attitude and the environment, how busy the place is coz you might be skilled you have the information but you're held up, maybe you're attending to an emergency so I'll have to…to look to another person. So you also look at the environment how it is if it's busy or not. And also…
	**G51: When staff are seen to do their work efficiently/ well (C) their efforts will make the work of others run smoothly (M) and they will be popular (O).**
	(Resident) Those that you can work with. The person you see you know what this person is reliable, reliability. One, if this person you give them a certain task, they don't leave that task unattended, they make sure that they do it up to the latter, yes. So mostly I like to work with people who are hardworking, people who are task oriented, people who are like, "You know what? We're going in and but you have to finish than we don't leave anyone behind." Even if they finish on their side, they will come and assist the other person, yes....Yes, a people work most of the time a team-oriented people. I don't like working with lazy people, yes....
	(Nurse) I just like how they work. Some are perfectionist, you see when in when they're in the room, taking care of the babies, you just see everything is smooth. ...Yeah. And they're responsible for everything that is happening.
	(Senior Nurse) You can find an MO you are this… I find a certain doctor on duty at least you are relaxed. A certain nurse, at least when you leave as a manager you leave as a leader you leave the ward you know at least it will be done.... They are willing, they are always flexible and positive about everything.... Yes and also knowledgeable. Knowledge also matters a lot... Yes. Because anything happens they are ready to handle they can handle it.
	(Nurse) ..yes very much like today I am comfortable I know my shift is fine....You know there is that…what can I say? There is that determination of doing… you have come to work for the sake… not like you have just come… to your time… you know you have just come to work and you have team work, teamwork, good communication skills. You see?
	**G52: When advice is based on shared decision making (C) it has more weight (O) as there is collective buy-in (M).**
	(Nurse) ...are we having a barrier? Mmmh, I think mostly it will go to, communication concerning maybe care of a neonate, care of a baby, from maybe the medical team to the nursing team...: I think sometimes, they, they give orders, with no reasons. You know, if you tell me, do this, put this baby on CPAP, I think you should tell me with reasons... not just, 'put this baby on'….., and its final.
	(Nurse) And now we, we had a discussion, the three of us, the nurse, the doctor, the two of us, nurses and the doctor. And now the doctor was, was able to say, let me call the consultant to confirm. So it was an easy way of making decisions because we, we took it, like I, I approached it in a nice manner, not telling the doctor, no, change this. I told, I was telling him, now I feel it is on a higher, the higher side as compared to the weight of the baby. And I feel like decision, making decisions or having that, having that, like not being on the same page in taking care of the patient, it is being taken positively and people are able to discuss it, and come to a conclusion

	(Nurse) Now since many people are able to voice their what? Their feelings or the…their view I see like it's usually… it…it affects positively because once a number of people have talked about an issue and then it is discussed upon you will get the best out… the best answer or the…the best way to do things. So even the one who had not talked will benefit from there.
	**G53: When individuals are afraid of feeling humiliated (C) they will not consult colleagues outside their own peer group for advice (O) as they do not want to expose weakness (M).**
	(Nurse) What I can say about difficulty in communication with others is that there is that difference in seniority. Others feel that they are too senior to consult the juniors. So like, they feel like if you know something, they feel like if you ask you they are demeaning themselves. ......
	(Student Nurse) But you see here most of our doctors are a bit reluctant to be helped. You see someone standing with something that you can do in seconds. When you tell them that want to help them it is like you undermining them. So, you give them a face and let them try and try and when you see, they are not able to do it when you step in. .... you can see they do not know what they are doing.
	(Student Nurse) But she could not buy my advice until a fellow doctor came in, told her the same thing and she responded. And do you know what, within one minute, the tube was inside, was inserted inside..... Actually, she acknowledged that I was very right, yeah.
	**G54: If a colleague is felt not share own knowledge with others (C) colleagues will identify them as not having a collaborative attitude (M) and will not approach them for help (O).**
	(Student Nurse) Personal factors, there are those who are willing to help. You know, there are people who can have the knowledge, but they are not willing to help you. So, yes, you will find someone who is ready and willing to help you.
	(Student Nurse) Yeah, yeah. The major issue is that these people, know something, but they do not want to share it with you... Yeah, they seem like we are studying to come and take their jobs. So, they give very minimal information. You can even ask for a cannula and they refuse to give you
	(Student Nurse) Yes, you'll approach her and tell her, I have been placed with you, you know there is that time, you don't know where to start, let's say for example I have been put in this placement, I'm not sure what I'm supposed to do, isn't she the one who is supposed to tell me, take this take the vitals, get this do this or that, right. She doesn't do that. You come in, and she tells you, if there is nothing you're doing, leave. So, you know, as a student, I take it that she doesn't want help maybe. So, I go to another place where I know I will learn or a place I know I will do 1, 2, 3
	**G55: When a member of staff does not take their fair share of the work, e.g. handing over everything to incoming staff, doing bare minimum, being absent from unit when on shift (C) colleagues will consider them unreliable and a cause of their own overwhelm (M) and so will feel angry, not want to be on shift with them, and they will develop a bad reputation (O).**
	(Senior Nurse) They, again, they always don't like taking responsibilities, they don't go a mile on their management, their work is just to feed babies, take OBS (vital observations) and document. Anything, a mother asking is that, 'I don't know, ask the doctor, ask the team leader in the office'. So they don't take responsibilities.
	(Resident) Characters that they don't care, those that don't actually care and bother. They just bring, "Okay, you know what, I came, I've done whatever I came to do for it, I don't care about whether there's someone else following it up, I don't care about whether there is pending things, let the other person who's coming next solve the issue," those kinds of people, uh-uh [No].
	(Nurse) ... heh, me seeing someone sitting down, (laughs), that thing irritates me.... you just come to work and then you just sit, a whole 6 hours, now what are you doing?
	(Medical Officer Intern) .. they don't like working. you can have up to six admissions and someone doesn't want to admit a child, you can be called for 5 reviews and they…. but they are not willing to… to work, to move around….
	(Nurse) ... mmmh, I think now, that pulling of the leg down is like too slow to accomplish things that needs to be done faster.... mmmh. like you're supposed to do a CPAP, someone wants to buy time until they want to handover to the next shift....you do a sugar, you don't want to put fluids, you know that kind of a thing?
	(Nurse) Me I don't like late coming..... Late coming, punctuality and laziness. ..Yes. And then somebody who needs like to be told what you're supposed to do. ... To be reminded let's do this, let's do this… me I don't like that one..... Yeah. I like somebody who knows what to do. ... You know because if Ill be doing this one, you be doing the other thing.
	**G56: When students/ staff observe a colleague displaying esteemed qualities (e.g. friendly, patient-centred, good work ethic, collaborative, qualified) (C), they will be encouraged and want to be like them (M) so will try to emulate the qualities (O).**
	(Nurse) About her, it's the way she does her work and the way she's patient. Because with our work here in the unit, you can be impatient at times. But she's patient. The way she takes you through how to do something, I'm saying that because she's the one I feel who mentored me. The first time I came in the unit, I was very green, and she was the one who was told to take me through. And with that, even as she can't even remember [laugh], and with that, I see how she works, and I, I emulate.
	(Student Nurse) For the doctors, despite your position, like for instance, inside here, the doctors are the most, I can say they are the most superior, if I'm not wrong, but they were willing to give information. They were willing to come to our level and we reason together. So I think that's something I would also want to, I admire that
	(Medical Officer Intern) ...she is very keen on patient care,,,, patient care affects her directly on a very personal level. When we lose a child, she, you can actually see she has personally lost something, you know...yeah. It's not just doctor-patient relationship, she…. yeah, she is really invested in what is going on in the unit, she really wants to make things better, she really wants to make things work, she really likes seeing babies come up, yeah, she is very passionate about whatever she does......mmh, mmh, coz I've learnt, from her I've learnt that aah, uuhm, I don't know the words to use, but I've seen what a good mentor is…
	**G57: When students/staff observe the behaviours of a colleague, particularly senior (C) these will appear socially accepted (M) and will be imitated (O).**

	(Nurse) I'll say the seniors, and those you, you communicate to mostly. Those who feel free with mostly. So if seniors are doing things a certain way, you will feel the need to follow in their way, if they're reluctant you'll be reluctant. Cause if maybe you may feel they are, they know something that you don't, they may have experienced something in the past, you know they've been here for long. They may have certain experiences with certain ways of doing things. So you do not want to start doing things extra [laugh]. And they have been here, they have seen maybe they, things have come and gone and they are still here. So you may feel now the seniors, they do really affect what we do.
	(Nurse) .. yes. I keep on telling these students, okay but am like what about the qualified staff? We sit we take tea when the round is going on.... what would you say about now, us, who are qualified? Leave alone students.. us, we've shown an example. You sit there, you take tea, the round is going on, you don't even know what was said, you come in the afternoon shift, someone is supposed to hand over to you, they don't even know what was said in the round. ...some babies have been put on phototherapy, others are discharges, and nobody knows.
	(Nurse) yeah, there is something someone will do, and you're like, this is not the right way,... and when you ask, they'll be like, 'no, in here, we don't have enough facilities to do such and such',... 'or we don't have such and such to perform the intended right way'...... So it trickles down to the students, we end up doing that coz….yeah
	**G58: If students feel judged/ othered/ dismissed by staff based on their student status (C) they will disengage (O) as they feel discouraged, do not feel a sense of belonging and fear making contributions (M).**
	(Student Nurse) Then, the, there was this, this nurse that, she was on our toes, like student from (name of school), student from (name of school), students, [laugh]. So I feel like, ah, why don't we leave the rest they do, the other students, you know there were many students here. So, I didn't like it....... we're not, eh, we're not making a lot of mistakes. Even if we were making mistakes, not that much..... I don't, or maybe, like today we come in late, we find the meeting has started, she'd be on our neck, student you are late.
	(Student Nurse) You see; one makes a mistake you generalise all of those people it… it's a challenge.
	(Student Nurse) Okay well I'm a student and there's a way seniors always treat students, so when you find that trait in nurses… it becomes like difficult to associate with them. So if I come on duty and I find somebody who whenever I ask them to show me something they don't or they judge me instead of helping me …I am usually not comfortable in that shift.
	(Student Nurse) I think you think its attitude towards to students. Yes, it is just attitude towards student in the sense that students are meant to do research, they should study, they should know the information themselves, they should not be asking too much. Yes.....Yeah, because I can see sometimes, they would even challenge us with questions that are obvious, obvious questions. So it was very hard to get information from them.
	**G59: When students feel more comfortable with one another than with staff (C) they may rely on one another for knowledge (M) limiting learning experience (O).**
	(Nurse) ...supervision wasn't really that tight... so, I think we were given, we were given, let's say if you're an older student, you were given newer students to supervise or, oversee over them, when they were doing something... so, from the seniors, there was less supervision..
	(Nurse) You know, there is a lot of calculations and in class we're not like being taught how to calculate. You get, you're being taught like these are antibiotics, these are types of that way.... Yeah. Like at a specific dosage, you know? But here, like, you know, it is, it's the actual thing. You, you, you work according to the baby kgs, that way. So we were doing a lot of interaction within ourselves, mostly on the drug part.
	(Student Nurse) Yeah, for emotional control, most of the time, I used to go to the students. By the way, sometimes it was traumatizing. You come, maybe in the night shift, you work well, you do medical management to a specific baby, but when you come maybe in the next shift, you find she's no more or he's no more. So, it was a very traumatizing experience. So most of the time, we used to console as students.
	(Student Nurse) More observation and skills, we see there are skills that you have to observe so that you can also do it, most of the skills that we learn, we learn from our fellow students, but I wish it was the other way around.
	(Resident) Basically, we just remind each other about the protocols because we are still learning the place, so we remind each other about the protocols, we give each other a lot of support when we are learning new skills because we're doing this for the first time. So, they will be the first ones to approach, you know, when I'm asking them a question about, that I think is simple...The third years, most of the time after, if I've asked a fellow first year and they're not sure about it, then we'll approach a third year to assist us with it, or if it's the first time we're doing a particular procedure, then we can just ask them to be there with us, even as we're doing it just to observe, to confirm we are doing the right…
	**G60: When students are studying different qualifications (diploma vs. BSN) (C) this may trigger judgements from staff (M) leading to friction (O).**
	(Nurse) …you find them they get things very fast whatever you tell them they pick very fast, when it comes to assessment the theory they know so much but when it comes to the procedure…the practical… okay, they…they, they know the science behind it but the real thing they are not very good at it.
	(Nurse) there are those people who are willing to learn and they are dedicated and there are those people that they'll observe you as if they are managers. They just stand, they watch you not knowing that tomorrow they are the ones who will be allocated in the same room but there are those who are willing to learn and there are those that even when they are doing something…... In diploma… okay that one… to me in diploma they…they have work at their hands.... Yeah, their hands are they…they are ready to work and they are ready to learn, when we come to degree most of them it's like that is not their work, they are more of management than on the bedside.

	(Nurse) Students, a lot has changed. But not even the students who have changed, everything has changed. Even the upbringing of our children is different. The children they have, I don't know what perspective they have. It's like those who are being articulated. They presume when they are training they are going out to become managers.
	**G61: When staff are patient with students (C) students feel more secure/safe (O) because their needs are understood (M).**
	(Student Nurse) She was nice. She was nice. She like, she would plan a time table, not time table, duty roster for students. So, like, like where you could feel, here, you could just go and explain to her and there was a way she would sort you, there is no way, you would leave there when you are like, you see?
	(Student Nurse) Most of the time you would find and you would consult them they would show you repeatedly without getting any… or without being annoyed… and the express themselves more and they give you the room to ask more questions more frequently.... Yeah, patient is the word.
	(Student Nurse) She is very patient... she can teach you, repeat 1001 times until you understand.
	**G62: If students are shouted at or feel humiliated by staff (C) their confidence will be lowered (O) due to fear (M).**
	(Student Nurse) When it comes to mentorship...Assuming I am a student nurse, I am asking something from you. There is a way you are going to deliver that message to me. Maybe I have a procedure to do... how do you deliver this? Whenever I am inquiring, how are you giving me feedback. There is someone you just go to and the way they handle you... say maybe rude, or anything... seems harsh and there is a way probably I have done something, and the way, you are correcting me. .. The manner in which you are correcting me.....
	(Student Nurse) I think it's the first impression I got when we first interacted... Harsh. ...uh Like how somebody communicates to you… …depending on…okay there is… I can come and ask you maybe what… maybe like something simple or I was doing something …instead of coming and telling me you do it like this you are doing it the wrong way, when you start shouting at me...I won't come to you next time.
	(Student Nurse) When you are being shouted at….. Yes, you'll have low self-esteem and you'll find that nobody even maybe believe that you can do so maybe you are doing something and someone, that's not done that's not done that way, so you will just develop the fear and you will just like doing things when somebody is not watching you.
	**G63: When students are from a certain institution (C) staff may refer to institutional reputation (M) and make assumptions about likely student attitude and competencies (O).**
	(Student Nurse) Students from (name of school), like it was a must for her to say, students from (name of school). ... No, we're not, eh, we're not making a lot of mistakes. Even if we were making mistakes, not that much.... I don't, or maybe, like today we come in late, we find the meeting has started, she'd be on our neck, student you are late.
	(Nurse) But it depends on the, on the area of, as in where, there institution, where they were trained. Yeah. They are just ones who are like (name of school). They're very good. Yeah. But you can get a group like (name of school) who are very slippery. Yeah. So, it depends.
	(Nurse) Let me tell you....For students It depends, students from (name of school) are good. You see I personally love (name of school) students. Number one, they will not run away, and then then they... OK, not all of them... Some have eagerness... But if you compare (name of school) students, I think they have the mentality that... they will not do bedside nursing... They'll just be in the office. …..Let's say you tell them to do this. Then they do. After that, they've not told you I'm going for a tea break. I've gone. They just disappear.
	(Nurse) Yeah, some when they come to work, they you can see it again at (name of school) you leave there the person needs little supervision. Degree, sometimes is good also depending on the university, so others somehow need time to adjust because some, maybe you have not seen the things in the university. Yeah, like that.
	**G64: When students fear approaching a staff member/ senior (C) they will consult one other (M) leading to poor learning experience (O).**
	(Student Nurse) The feelings and emotions we discuss among ourselves, and among the students. ... I would fill by sharing my emotions today with the qualified, let me call them the qualified. We are exposing our weaknesses and so much as well. .. Yeah, but we feel like it is better we discuss among ourselves. Unless something is so emotional that will require us to talk to our clinical instructor.
	(Nurse) Yeah. It largely, it, it affects. Cause you just imagine you do you, you're not familiar with something, and you are just quiet you don't want to ask. You'll end up doing the wrong thing. And that will affect the delivery of care to the patient.
	**G65: When digressive behaviour is witnessed by students and not dealt with appropriately/ normalised by staff (C) students may not know who to turn to for support (O) as they will be confused about staff values and who to trust (M).**
	(Nutrition Intern) .... And I was just like, I don't know what we are supposed to do! Cause like the people that we would have approached, you know, with this behaviour, are the ones who are present when these behaviours are happening. When these words are being said, they're present in there, and they are like, and they just laugh it out. So, you are like, okay. I guess that's the new normal, and you just move on.
	(Student Nurse) ... so, you get so stressed, you don't know who to talk to... you don't know whether to continue with doing things as they, okay, you know they normally say, there is this quote ' kwa ground vitu ni different' (reality on the ground is different). .. what you learn verses what is being practiced are totally different things....
	**G66: When staff correct colleagues in a positive and respectful manner (C) advice will be more accepted (M) and behaviour will change (O).**

	(Nurse) Yeah, they do. Of course, we just discuss, I don't just, you know there is a way of discussing a procedure and giving the information the right way. You know, I find them accepting because it comes slowly and we discuss this the way we should go about it and they accept it.
	(Nurse) You tell you don't just come and shout. You are supposed to do this like this, but you take a lead. You show them what they are supposed to do. Oh, okay. Yeah.
	(Student Nurse) Yeah, so even if I'm… I've seen a mistake I usually don't approach it in a way that I know it all... Yeah. I usually tell them so when you do it like this is better than that way. ... So we tend to appreciate more than… more than if…
	**G67: If staff resist correction (C) colleagues may disengage (O) to avoid confrontation or because efforts are considered futile (M).**
	(Nurse) In terms of communication, there is not that mutual respect one nurse for them. If you find you like to correct someone, then someone is answering in a rude manner, it's that communication issue. ...Yes, they do not acknowledge that they need correction.
	(Nurse) So it looks, it depends with how approachable.... Yeah, the person is because there's someone you can tell and get the person angry but there's someone else who'll just take it positive, the person won't take it negatively.
	(Nurse) So you tried to tell them……someone tells you like, that thing don't tell so and so about this because they might take it personal or something ……
	**G68: If staff are deemed to have a superiority complex (C) colleagues may disengage (O) as they perceive them to be resistant to change (M).**
	(Student Nurse) I think it just comes with the fear…the fear of how it will be perceived. Okay, you know, I don't know If you've experienced it but there's something about somebody who's above you...... There is that I don't know how I can describe it. If somebody is above you they'll always want to show you they are above you. .....: So there's no way you'll correct them at that point. .... Yeah, unless you use somebody else who you're comfortable with to talk to them. .....Yeah. If somebody is doing something and I think it's…it's not right and if I don't speak of course it has affected the care that person would have received.
	(Nurse) It depends on who, yeah coz definitely not everyone that you can offer advice to, yeah coz you see in terms of experience and age there is that person who you may think now huyu ananiambia nini [what is this one telling me] we have been here for so many years. So at the end of the day you look at who…who you're talking to.... I'll al… I'll always choose to tell that person you know that will listen to you and will take it positive, yeah coz irregardless whether you are young or old there is that person that you can tell something and they take it serious. ...Yeah, and there is that person you will tell and take it negatively, yeah.
	(Student Nurse) But you see here most of our doctors are a bit reluctant to be helped. You see someone standing with something that you can do in seconds. When you tell them that want to help them it is like you undermining them. So, you give them a face and let them try and try and when you see, they are not able to do it when you step in.
	**G69: When a staff member adopts an encouraging/ polite/ lovely way of correcting colleagues (C) colleagues will not fear them (M) and are more likely to accept correction/guidance and improve (O).**
	(Nurse) You tell you don't just come and shout. You are supposed to do this like this, but you take a lead. You show them what they are supposed to do. Oh, okay. Yeah.... It cuts across the doctors
	(Nurse) You see now, when I'm on duty, like now I'm working with somebody who is somehow lazy. That day, that person will work hard because he doesn't want to me to see he's lazy. So, I have influenced that person to work hard that day especially when I'm on it because they say, "Today you are with someone who is lazy. This day you are going to be working alone, almost." But when I arrive, there is a way I can talk to that person. So, I have arrived on night duty, there's a way I can talk to that person and we can work very much. That night we can do a lot of job, now the laziness now is gone. Unlike scolding somebody, there life both of them are just there, they can't work hard......Yeah, very much because I know how the way I can talk to that person and the way I can work can't sit down now, this person is working hard. So, I can join. So, we work the whole night, we work.
	(Nurse) Well, this one is an intervention that I take one on one, because you don't make it a general comment, you take one person whom you see has got a weakness or a problem about handling certain issues. You take time, when you are free, you sit and discuss it slowly by slowly and then you make somebody understand. So that is the only way but if you bring it generally, within the setup, somebody might feel you are undermining them in the process of their work. Yeah.
	(Nurse) We had one who was in management of patients. Sometimes when you are giving a report she could discuss other things, and mothers are around. Okay. So I just talked to the person that is not good to give reports when mothers are around but there are some confidential messages about patients that we should just discuss among ourselves because if you discuss a patient and the mother is around and that's not the mother of that patient, this mother can pass the information to other people. And then she will.
	**G70: If a leader/ staff member feels a member of staff is 'rigid to change' (C) they will feel their efforts are likely to be futile (M) so will be discouraged to give directive to the individual (O).**
	(Nurse) ….. I realize people look at me like am trying to be too tough on them... so, I let it go.... say, the T-sheets are not signed, they are not signed. I won't ask.... I won't press anyone.... but initially I would do that. I would correct. you must sign this, if you give this, you must sign. .... but then I let go....

	(Senior Nurse) Mm… I have not had an experience those who are difficult but yes they might not be so much difficult but there are some who are not flexible..... Because for us they believe if it is this way it is this way. When I want to do it when I don't want to do it so..... Yeah. Maybe there is something you want to discuss about maybe a management. You see they tell you this is why I cannot do this because of this. So once even you discuss they still remain rigid..... They don't they don't see the need of maybe whatever you are doing.... Yeah they just feel it's not of any importance to them.
	**G71: When staff are identified as reliable (C) they will attract allocations (O) as leaders will be confident the work will be completed (M).**
	(Senior Nurse) Neutralize yes [by pairing up with other staff]. You know when you neutralize them you will not even notice this rigidity. At least they will find out oh whoever is working also I'll have to do it. At least you try to motivate them to do it. Maybe they fear that what I told you lack of knowledge or experience they are not competent.....Yeah so they can try to work.
	(Nurse) It could be the juniors or even there are some seniors but most of the time is the juniors, utaona(you will see) 'Mum can I set up your trolley ….what do you want me to do for you', you send them to get something there are even seniors who can go, but it depends on people.
	(Nurse) OK. When we have new Staff, there are people nominated to mentor that person, but supervision is mostly done by the team leaders. In every shift we have team Leaders on the ground, and for example, if today I am allocated in the (HDU). With a new staff, then it is automatic that I am the one who is going to mentor that new staff. So during allocation, they normally look for a number of things, their experience and their level of education. For them to balance. The allocation.
**Access to knowledge, skills, experience**	**G72: If a colleague's scope of practice is most aligned to query (C) staff will seek their advice (O) because they are most likely to have relevant knowledge/ skills (O).**
	(Nurse) Like the doctors, the doctors, well, I speak to them like for example, let's say I'm in a room, the doctor who is in that room, I'll constantly speak to the doctor because I'll need, because you see we are… Yeah, we are collaborating on the care of the babies. So if for example you, if I have IV lines, if I have a very sick baby, you see we would constantly talk with the doctor then I'll see the plans that he has for the babies on that particular day.
	(Nurse) I also speak to the cadre, I speak to everyone, but now, depending on now what I want to ask. If it is something concerning the equipment, there's X to ask, to assist in preparation and also to help in making me understand more on how it works because we have so many equipment's in the unit.
	(Nurse) Usually we communicate to our doctors in case there is a problem, ...OK, you are in a room... something has happened to a baby. Like you need a doctor... Maybe you are resuscitating, and you need the doctor to come and help in the resuscitation, that is when you call them. ... For the nutritionist maybe, maybe there is a baby who has been prescribed nutrition...some fluids or feeds for nutrition, usually they are usually with us, so you inform them like today this baby has been put on Iv fluids/feeds please calculate for us because they are the ones who know how to calculate. The doctors only will prescribe but the nutritionist will now calculate according to how I think the calories, then she will write on another form, then we will, us, we will now follow what the nutritionist says, we put the fluids according to how they have been prescribed by the nutritionist
	**G73: When a member of staff is deemed to have the most 'finger-tip' knowledge about a patient or the ward, e.g. nurse, COI/MOI (C) this will be valued by other staff for decision making (M) and so the member of staff will be sought for this information(O).**
	(Nutritionist) It depends on what advice I want about the patient, because the first person who'd engage is the, the primary nurse in that room of the patient and the, the other person is the primary doctor of the patient. He or she can also, depending on what question I have, because with the doctor, I would want to know what is the plan for the baby with regard to maybe fluid intake or what has changed, because now this baby's behaving this way. And for the nurse, maybe I would want to know whether the mother has shown up or if there is, if she has, she would also be telling me that this mother doesn't have enough milk. So it gives me a head start of my work. When the mother's come in, I know that I need to prioritize so and so, or she'll tell me, this mother has a challenge with positioning the baby in the breast like that. So that is how I choose. I can also choose to look for the in charge of the unit. If I feel it is something the primary nurses cannot answer.
	(Consultant) Ward manager to report on clinical issues or any other, my 2 MOs, primarily the one on call, we speak a lot during the day and night, mostly for consultation purposes and for the running of the unit because they are there a lot longer I am and they are more immediate to the interns and the students.
	(Senior nurse) Mostly first we ask whoever has been managing, like I'll ask the night staff, how has this patient been? Because maybe this, the staff will say this baby has been having dyspnoea while on oxygen or not, it's still dyspnoeic. Then you also, okay, discuss with the day staff, the ones that have also managed this patient, how do you see this patient? Then we also, now after we get our, okay, the basis, now we discuss with the MO and we, and we can also escalate to the, to the supervisors of the MO, is the consultants.
	**G74: When a senior person gives advice (C) it has weight (O) due to status and authority (M).**
	(Student Nurse) The one which I'll give a lot of weight, it's maybe, let me say for the nursing, eh, the, the nurse in charge, for example, she is the overall boss and knows of everything that is going about, so that coming to a specific decision, it has taken a lot of consultation, now it will have much weight than a nurse who is communicating about something. Yeah. So for the nurse in charge and the people who have, the higher administrative, I can take much consideration.
	(Student Nurse) I think the neonatologist.... whatever she'd say, I would take it to be the gospel truth... she was very knowledgeable.... like she can teach. Like she teaches, and repeats, she's patient.... very patient. She understands that you're a patient and you need to learn, and she will repeat as much as possible... until you get it.
	(Nurse) If the consultant says do this, you don't have to question, (laughs)

	(Medical Officer Intern) ....aah, if it came from the consultant, I will not question. I do not…we don't question consultants. We do what they say......it doesn't matter...I will do what the consultant has said.
	(Nutrition Intern) I'll probably believe if to be the gospel truth [if information comes from senior]. Because then, I don't, like I don't, this is probably something that I don't know how it's done. You know... if say, they told me, babies under say 1 week, are supposed to be on this type of feed,... if I've never heard of the feed, this is my first time am interacting with this baby, I'll take that information to be the gospel truth, because anyway, they've been practicing longer
	**G75: Staff/ students will seek advice (O) from one within their own cadre who is seen as more knowledgeable, more specialist, or more senior (C) as they have expertise but are also approachable (M).**
	(Resident) Yes, for the part threes, also it was, we were, we were communicating well, because we also felt like they were our colleagues. So anything, any questions we had, we were asking them, and it was better because they already, they already had experience in NBU. So they were able to guide us better. Yeah, the part threes were really good. They were really good.
	(Nurse) Well, I would… it depends on the work I want to be assisted with. So I will look at the nurses and see who can be able to assist in that. Like if I'm in ICU with ventilated babies, the babies on ventilators and I see I need assistance, maybe the baby has removed the tube… …I will look at among them who is an equal nurse, who is a specialist nurse and that is the person I will call.
	(Nurse) It depends with the team that we're working with. So, if we are many, I'll find that I'm talking to the most experienced more than the less experienced based on the condition of the case we are dealing with.
	**G76: If staff have specialist qualifications (C) they will be sought for advice (O) due to their superior knowledge (M).**
	(Nurse) Yeah, within the nursing team, there are people who are the special, specialized in certain areas, so we must also listen to, to them. We have neonatal nurses, critical care nurses, there are physiotherapist and occupational therapists. So we have to get that advice and take in what is right for the situation we are in. And in the nursing profession, we are different cadres. So we have to listen to one another for us to perform our duties well.
	(Nurse) Yeah, because these two are what we call ETAT trainers. So, they know more. So, they've done they've gone through this training, they are also specialized neonatal nurses. They know more. They have more knowledge.
	(Nurse) Well, it will depend with, if work related issue, it will depend with what, what kind of help am I seeking? If it's something that is critical, for example, if it's a baby on CPAP or mechanical ventilation, I'll approach someone who has done neonatal nursing... The common task, now I can approach anyone.
	**G77: An individual with relevant experience/ training/ done same course (C) will be sought for career advice (O) as they have relevant and valuable insights (M).**
	(Nurse) So you look at maybe how they work, their seniority, how many years they have worked what different places they have worked except the NBU. And maybe your... Yeah. Exposure. Cause you may talk to somebody who has just done neonatal nursing, has never worked anywhere else. So maybe they'll encourage you to be in neonatal nursing. But if you talk to somebody who has maybe started as a general nurse, did a diploma, later did a degree. You may find a different reasoning from somebody who was, who just did neonatal nursing and has not done a degree, or somebody who did a degree without doing neonatal nursing or a higher diploma. So when you talk to somebody who has an exposure, you, you're able to make your decision well.
	(Nurse) .. mmmmh, I think most of the times, okay, I've talked to people about that, but I think what I considered is because this person has done this course... so, how do you feel about it? is it a challenge? The time? The timeframe?... and how can I manage between my work, and that course and, yeah... the person has already gone through the course, coz they've done that.
	(Senior Nurse) So what we normally... what I normally…what I do, whenever I want to do something also carry… you always look for that person who has a qualification or a personal experience. At least seek, ask them how it is because I cannot ask somebody who doesn't know what it is. .. Yes at least you search… look for somebody who at least has knowledge about that whatever you want to do.
	**G78: When an individual displays skills and knowledge through their actions (C) others will go to them for information and advice (O) because they have demonstrated genuine quality in their practice and are therefore trusted (M).**
	(Nurse) At times, it's how also people work. How somebody works. You're able to know, this person knows how to prepare something. This person knows how to deal with something. Now, with that, it cuts across, I can go to anyone. Cause they're those seniors who don't know how to do something things, they come to the junior. So working is easier because we are able to know this person, it's, it's somehow in a, now in our, in our unit now there are people who we know they're good at something.
	(Nurse) The competence, competence, how competent they are, because for example, if it's IV line, it's not everyone who is good at inserting IV lines. Yes.
	(Nurse) competency, competency... mostly, through observation.... their standard policy of procedures. If it's followed or not...for instance, drug administration... there's a way you administer drug like dilution,.. safety, safety to ensure nini (whatever), all the rights of drug administration are followed... whenever someone is eeh, you can just judge.. by observation. Does he follow the procedure or not?.. yeah, and the way she…, maybe you're, just aah, on a normal chat, as students pose a question to a colleague, but the colleague has no capacity to answer, he or she refers the students to you... mmh. That's when you can gauge competency

	**G79: If staff have worked on the unit for a long time, or are usually allocated to a specific sub-unit (C) others will assume they have experienced similar patient/ward scenarios before (M) and will go to them for advice (O).**
	(Nurse) Because if maybe if I have something like it's a resuscitation he's somebody that will respond very well, and he's somebody that you can talk to and for sure she…she is a senior in a way that she…she has been in the NBU for a long time, so if you want something that she explain to you and she will even show you.
	(Student Nurse) Yes. Older nurses, the reason these people have worked there for more than 10 years, 20 years, they have seen a lot. When you are met with a challenge you ask them first, but since they have the old knowledge, you get that fast. And then you look for your colleague. You feel when you feel they are going the same route, you know you value both. The most people to value are the old nurses.
	(Senior Nurse) There are those nurses who have worked in the unit for long maybe there is something you need they want to know about NBU you find out from them. Me personally I find out from them what they used to do or what they do about it.
	(Nurse) Conversant with that room. Maybe I've been allocated NICU one, and I'm usually not allocated there. I will go to the, the neonatal nurse, those who are usually allocated there to consult.
	**G80: When staff in other departments perceive neonatal unit staff as having knowledge and experience (C) they will value the advice of current and former neonatal unit staff (O) because they assume competency (M).**
	(Nurse) From staffs from NBU [neonatal unit] mostly because they have experience on the babies...Yeah and maternity... ..Even the, the doctor in the ward, I will not take their solutions until I consult the guys in NBU...Yeah because they have much experience with them... Yes, because they handle most cases.
	(Nurse) Yes, I can go for a second opinion. And also, I will still say it also depends with who gives the, the information that looks contradictory. You say like, for example, if I have a colleague come from the New-Born Unit and you see these are guys who really a hands on with the newborns, as compared for with us who most of the times you conduct a successful delivery, your time will be quite limited as compared to them, then they tell you maybe we have a new protocol or this procedure. So, I might consider someone from a New-Born Unit giving me a piece of advice on a special or a respective procedure that I'm performing. So, I will consider a second opinion but also based on from which source.
	(Nurse) Okay, in this eh, situation, assuming it's a baby, we have nurses who have been in that new born unit and they are very good in resuscitation, they always want to, I always consult them, and they are very good in what they do.
	**G81: If a member of staff is felt to have poor skills (C) others will not go to them for help/ advice (O) because they feel the member of staff can't help them, and their approach conflicts with own knowledge of good practice (M).**
	(Student Nurse) .. but the ones I interacted with I felt like, this is my own feeling, I felt that they were not that knowledgeable... so, (laughs), like they were making some funny, they were even giving some diagnosis you question like, heh! Okay, I am a nurse, but I don't think I would do this
	(Nurse) ...because, sometimes you find, you find someone, you feel the skills someone has, you have more better skills, so, it's like you're downgrading yourself like, you're already sure, hah! hawezi nisaidia, unaona (s/he can't help me, you see)?
	(Nutrition Intern) ..probably from the output I've seen, with the work that the've done, I'll be like, aaah, eeeh, you know?.. from what've seen, output, like the output I've seen them give at work, how they do their things. You know .....taking advice from them, would majorly really depend on how I've seen it work here, but what others have said will influence how I will interact with them....

EMPOWERING	
Facilitative hierarchy	**EM1: When there is a hierarchical hospital management structure (C), senior leaders will receive formal information (M) to disseminate down their professional silos (O).**
	(Nurse) Through the in charge or through the team leaders. As in they start hierarchy, for example, if our in charge receives any information from the senior (Nurse), then she conveys the, the information to us. We have a WhatsApp group or even during the CME days; the weekly meetings.
	(Nurse) ... aaah, I think the, like a unit, the, let's say the (consultant).... comes and talks to the in-charge, then the in-charge talks to the nurses and the nurses to the students.... it trickles down again....
	(Resident) From the consultant to us directly, sometimes from the fellows to us.
	**EM2: When there is a hierarchical structure (C) juniors and students will identify their leader as gatekeeper (M) and will depend on them for information (O).**
	(Student Nurse) The information comes from the… from the nursing in charge of the ward… then it goes to the assistant. Then from the assistant in charge we have the team leader... then from the team leaders the information now is everybody gets the information from the team leader. And for example, when the team leader is not there… when during the ward, time when working, if you want something you inquire first from the team leader. ... If the team leader is not in the capability to do that you go to the assistant, if the assistant is not able you go to the nursing in charge. ... But overall information just comes from the nursing in charge.
	(Resident) Through our Chief registrar. So, the chief registrar will tell us whatever the admin wants us to do. They want us to cover a particular area.
	(Student Nurse) I think it is systematic, because it would be wrong for me to go directly to the in charge by passing my student instructor. So I first go to the student instructor before I go to the in charge.
	**EM3: When a leader recognises their role as gatekeeper (C) they can intentionally manage information transfer for team benefit (M) leading to more effective communication throughout team (O).**
	(Student Nurse) If there's information concerning the student, we used to receive it from the instructor to the team leader, the student team leader. Like I was the team leader. So sometimes (student mentor) would call me, or what would WhatsApp me about something going on, or what we are required to do and then I, I disseminate it to the students.
	(Resident) We would receive through the, our rep, our representative. So, they'd communicate to her then she posts in the group. Like, "This is what has been happening this week, or this is what's going to happen next week, this is how we're going to do it."
	(Nurse) So, we normally have circulars. So if a circular is written, it is directed to the ward in-charge. So the ward in-charge has the responsibility to disseminate information to the people on the ground the nurses in the unit. Obviously, it comes up to down. The same way if you have an issue in the unit, you have to relay to the ward in-charge the ward in-charge relays to the senior assistant chief nurse is the one who is covering the department from here it goes to the one who is covering the post as director of nursing services.
	**EM4: When there is a functioning hierarchical structure (C) there is clarity about information flows and processes (M) which can assist oversight and communication (O).**
	(Nurse) Well, I will communicate more with the… you see the in charge as long as I've gotten the report from the room, if I find there is something that she needs to know about I will communicate with her because there are things that the in charge… the in charge will care to know. If I am the team leader, and something crops up, even when she's at home and I feel she requires to know, because there are things that can happen that she will require to know, she will be even be contacted from above…
	(Clinical Officer Intern) You can call our consultants direct... Yes. So if there is any issue and no change has been done, you can still consult them...It makes you feel good. You can feel comfortable doing your work… because you know in case of anything, I am free to call anyone.
	(Nurse) … there is two pathways. One is for the case of nurses information will flow from in charge to the team leader… to the primary nurses... Yeah for the case of the doctors from consultant maybe to the team leading doctor then to the other doctors... Then information from us to the doctors… we interact at the ground level… from the nurse to the doctor, maybe there is something you discussed in a meeting so you…you just go and inform the doctor and…and also the team leader can inform the other team leader of the doctors so that they are also aware that's maybe the… maybe I would say the structure.
	(Nurse) My immediate supervisor is the in-charge. And also in every shift, there is a team leader. So, those supervisors, they supervise, if there is anything, we consult them because they are the immediate supervisors.
	**EM5: When staff follow the instruction/ leadership of their siloed cadre-specific hierarchy (C) leadership consensus/ collaboration (M) is required for unit-level change (O).**
	(Consultant) Then, the other is just on consensus building, you know, agreeing among us, do we need to do this? What is the aim of this? Do they buy in? because if you push as an individual, you are bound to lose out. So, convincing the rest of the team that this is important, coz communication is 2-way, it can't be 1-way.

	(Consultant) It is frustrating…because the cadre lines are very clearly drawn in our public facilities, so I have to take it with caution. It is very difficult for me to discipline a nurse regardless of, you know, of the occurrence. I have to go through their ranks.
	**EM6: Medical doctors (C) are deemed to have relative authority over patient care decisions (M) and are therefore sought for patient care advice (O).**
	(Nurse) Definitely if it's coming from a consultant, I'll give it more weight. If it's coming from the MO, I'll also give it more weight.
	(Nurse) I think I'll have to ask now the senior most first, not first, but I'll have to seek opinion now from the, maybe from the doctors now.
	(Nurse) Because the Doctor is the one seeing the baby, the doctors are the one who reviews the patient, so you talk to the doctor who is allocated in that room and if you feel the doctor has made a decision that is not so adequate, maybe you can talk to the consultant first you have to talk to the doctor in that room then you tell them you are not happy with the decision you are making or we can look for more other decision so you can call on the consultants we discuss together.
	**EM7: When hierarchy is clear (C) knowledge of etiquettes and brokers, including who they cannot approach (M) will be used by staff in selecting who to go to for advice (O).**
	(Student Nurse) Being a student you go to the primary Nurse who you are working with. When you notice a dose is high or low, you inform her and tell him or her if you do not mind, you can involve the doctor. If you see she is also resistant, you go to the team leader, and the team leader will intervene. .... Yes, you have to follow that chain.
	(Student Nurse) So the first person that I normally go to is my colleague, the one that I'm working with, a fellow nurse. That is where I start from. If they are unable to sort it out, I move a notch higher, consult the doctor who is on call at that moment if unable to, I also make my own judgement. I can still upscale to the fellow on call if he or she is not around. Yeah, if the fellow on call is unable to handle, we escalate to the consultant..... It's vertical. We start with the nearest.
	(Medical Officer Intern) .. like when you are deciding on admitting, whether to admit a child, okay sometimes you can decide on your own but then at other instances, there are situations that are grey, you don't know whether to admit or not. So, in such a situation, the person you call is the MO. ... Then when you are not sure whether the patient can be managed within our facility or cannot be managed here, the person you call is the consultant....... eer, coz you know in a day you admit like, you can admit up to 8 babies. I don't …. (laughter). I wouldn't try to call the consultant 8 times a day.... coz we have MOs, we have MOs. I will talk to my MO first before I talk to the consultant.
	**EM8: When hierarchy dictates that nurses should approach the most junior medical staff first even when they feel this is inappropriate (C) they may feel undermined and frustrated (M) leading to either circumvention to consultant, or tension between cadres (O).**
	(Nurse) That one I think it's based many times with the issue… it's just some not all, but when you reason together you you come to an agreement. It's about like if I'm communicating an information like as a nurse to an MO he would ask like you're not supposed to talk to me directly. Is there an MO intern on the ground? Are you seeing.......You know at times maybe I have something which needs to be done. Maybe the MO on the ground is busy or we are not reasoning together. So like I feel like can we… I talk to the higher level. So somebody will know let the MO on the ground call me you see?.... So that is a barrier. ... Hierarchy of communication now.
	(Nurse) Mm, Yeah. Not much but, maybe the communication between like the consultants and the nurses. Yeah. Should improve. Should improve, because sometimes you have some MOs you cannot approach. Yeah. You call an MO, she tells you, have you talked to the intern? So you see. Yeah. And you know, intern is a student. ... So my feeling is I wish, I wish we could be on the same page when discussing a patient and not to tell me I talk to the intern, and I'm qualified nurse. I've worked here for five years and an intern is just here for 12 weeks.
	**EM9: If a nurse feels a patient life is in danger due to the decision of a junior doctor (C) she may attempt to override the decision to save the life (M) and may speak directly to the consultant (O).**
	(Nurse) There are others that you can just let it slide but if it endangers the life of the mother we override them and we go to the consultant.
	(Nurse) Because the Doctor is the one seeing the baby, the doctors are the one who reviews the patient, so you talk to the doctor who is allocated in that room and if you feel the doctor has made a decision that is not so adequate, maybe you can talk to the consultant first you have to talk to the doctor in that room then you tell them you are not happy with the decision you are making or we can look for more other decision so you can call on the consultants we discuss together.
	**EM10: When staff of the same cadre form a WhatsApp group (C) this allows immediate and complete dissemination of information and notices within the group (M) making them a crucial source for awareness and belonging/ identity (O).**
	(Nurse) And also we have a group, a social group, whereby we communicate. In NBU social group, we communicate. .... Yes, the WhatsApp. If there is anything, any minute, any time, any change, we communicate. For everybody in NBU, we are there. Just nurses.
	(Nurse) Not all staffs. Like ours is for only the nurses that we are there... Yeah. It's nursing related.
	(Clinical Officer Intern) …not as per the departments, the Med sup will go through our coordinator then she will communicate to us. We normally have a WhatsApp group for all the COIs.
	**EM11: When WhatsApp is used to share administrative updates (C) access is easy and transparent (M) and dissemination is effective and equitable (O).**

	(Student Nurse) If there is a proper coordination so that the information can reach all the people, maybe it can be maybe via texts. Common message or via WhatsApp group, so the each and every person can get the information.
	(Student Nurse) We have the WhatsApp group.
	(Senior Nurse) The…they always write a memo... The hospital writes a memo and then its posted it is distributed in every department but what we also do is done even via WhatsApp.... So when it comes we have a forum for the whole hospital nurses though there are some who are not in but still every department I think has a WhatsApp group. So whenever that day comes you take a photo and post everybody to see.
	**EM12: When senior staff respond quickly on WhatsApp (C) it may be an effective way of gaining expert advice (M) so staff may use it to aid decision-making in patient care (O).**
	(Nutritionist) ..... aaah, okay, if there is, let's say for example, something to do with feeding tolerance... she can scan a bit of the guideline, then she send to me.. share to me, so that I can roll it down to the, to the mothers and agree on a common ground....
	(Clinical Officer Intern) Yes, we do. We have the pediatric WhatsApp group.... All the MO, the MOIs, all the COIs, all the MO qualified.. And consultants..... You can share...It is immediate. Yeah, it doesn't take long because mostly you find that the qualified people, most of them they're online..... Maybe [you receive an answer] after like 30 minutes or 20 minutes. But if it's urgent, you post then you make a call.... For example, you're sending an, an x-ray, or you're sending a lab result and you need it to be interpreted, you just send then you call.
	**EM13: When there is a group/ team leader (C) they can be a gatekeeper for communication (M) disseminating administrative updates (O).**
	(Resident) Yeah, she's a part three team leader, so she's also team leader for the whole of NBU
	(Student Nurse) Most of the time you were called by the, what's she called? Student leader (referring to student mentor). If there is something she wants to communicate, she calls all of us..... Yes. She would call us together and tells us. Or she tells our team leader (our student team leader), she calls them and tells the student team leader to pass this information. Then he would pass it to us via the WhatsApp group or sometimes if he gets us at school, he tells us.
	(Student Nurse) So from the in charge the communication is given during the morning reports from the in charge but maybe some information can be passed from the admin, to the school, then the school can reach us.... We have the WhatsApp group.... Noticeboard, the canteen, memo.
	(Resident) Through our Chief registrar. So, the chief registrar will tell us whatever the admin wants us to do. They want us to cover a particular area.
	**EM14: If a nurse is self-confident and feels they are acting in the interests of the baby (C) they may ignore the directive of a doctor if they oppose it (O) as they feel justified (M). (less strong)**
	(Nurse) ... they want us to be transferred because now we know a lot, we are directing them. Yeah, yeah, yeah, we are directing them on what to do, because their word is final, they believe. Yeah, they believe they are the final people, us we can't say anything, we have to follow instructions. So, now, that's another thing.
	(Nurse) .... I was told to write a statement. Of which I declined.
**Constructive centrality**	**EM15: If a member of staff has a diverse scope of work, e.g. nurse in charge (C) they will have broad responsibilities (M) and therefore will communicate with colleagues across the healthcare workforce (O).**
	(Nurse) Well, it depends on where I am because if I'm with the team leader I will communicate… I find myself communicated… communicating more with all the nurses in all the rooms not any specific one. But depending on the situation maybe the nurse who needs assistance I will communicate with them more. Then when I'm in the room I will… I will be communicating more with the… with the mothers, when the mothers are there of my patients who are babies and also when the mothers are not there I am in the room and the doctor is doing a round we'll be… I will be communicating with the doctor when doing the ward round and all that. Like I said, I'm also sometimes tasked with the students, so when I'm with the students I will be talking more with the students, so it depends. If I'm… maybe I'm the team lead another person I'll communicate with is the… is my boss, my immediate in charge. In case I need to inquire more from her and she's giving me maybe some report some things that I need to do. So it all depends on who… where I am.
	(Nurse) Okay, I speak more to nurses and medical officer interns because I interact more with the nurses. So, whoever we are on duty with, I must talk to them, because they need something clarified or we need to collaborate on what to do in our jobs. In case in the management of patience, we do work as a team. So, I speak more with them, MO interns are the ones on the ground, so I must talk to them for every case that I get. So, I speak more to them than the MOs.
	(Clinical Officer Intern) you know like in a day you can find that you have interacted with almost everyone. Every shift you interact with the ones who are there also the ones who are leaving. I used to interact with everybody there is nobody I was not interacting with.
	**EM16: When staff have a clear supportive role on the unit, e.g. team leader, in charge (C) they will be a reliable approachable source of information and help for diverse staff (O) because of the responsibilities and authority associated with their role (M).**
	(Nurse) thankfully, you always have team leaders in every shift. I'd approach the one who's currently present just to, to hear their views. And then I'll be like explain how this is done, even if I know, and there is that person who mentioned how it's supposed to be done, so that I can have clear information on how I should be going about the filing of that document.

	(Nurse) Mostly you, you, you ask the team leader. ...Yeah. The team leader. Anyone who is senior. The ones who have the, the ones who have stayed there for long. Mostly they're the ones who know more about the, how the hospital works, how the, how to manage the babies. Yeah.
	(Nurse) ...for instance, our in-charge, our in-charge plays administrative role. Of which, what she…, her input will have more weight, than the rest of us. Because of the administrative powers she has.
	**EM17: Junior doctors have a broad scope of work and are under the consultant (C) so are approached by all cadres for patient care decisions (O) because they have 'custodian status' of medical decision-making authority (M).**
	(Medical Officer)... If it's medications……for example, we talk to the nurse giving the medication.... So actually, we review all T-sheets on a daily basis. So if we see a medication is missed we actually tell the people giving the medication why is this not given. Then there is feedback either there is no line or stuff like that. ...So, whoever is on the ground on that day giving the medication we talked to them... If it's now further consultation where we don't… we don't know what to do or we are stuck then we call the consultant. .. Then if it's like other issues like management things … So depending on that, we can talk…we talk to social workers as well. ...Yeah, those technical things we talk to nurse manager...
	Observations - strong
	**EM18: If a member of staff is shy, or worried about possible consequences (C) they will not speak out and may quietly implement their own plan (O) as they will try to avoid conflict and assume someone else will speak out (M).**
	(Nutritionist.) A little I can't say one hundred percent. I can't say a hundred percent. Maybe I'm the one who's scared to speak out, especially when something is going bad......Because you see maybe whatever you are going to say is going to affect someone else's job.
	(Nurse.) Yeah, it is individual but now, you see when somebody is at the wrong, we are very many here, someone else will see and report to the, to the team leader and then from the team leader to the in charge.
	(Nurse) ...you know sometimes, sometimes, sometimes you value that co-existence, more than arguing..... I think that is the thing. Because, if we start arguing, ... then we are not proving each other right or wrong. We're just arguing, you see?
	**EM19: When staff prefer to work alone or avoid communication with colleagues (C) the enjoyment of work perceived by their colleagues will be reduced (M) so colleagues will not want to work with them (O).**
	(Student Nurse) you know I like the place I'm in to feel as if you, you are wanted there? ...Yeah. That you belong, not that you're just somewhere, like you're on to your own thing, they are to their own thing, when you ask something it's like they are not interested in answering you
	(Nutritionist) As in someone who's uninteractive, you talk to them, they don't want to respond. Their mind is somewhere else or, yeah, such. Yeah.
	(Nurse) Yeah, because there are those I can say it's like it's their nature, they don't talk much. For example, if it's the team leader, you see team leaders, they are the ones who write the report. Maybe than one she will just sit at the desk or she's on calls, the telephone calls and write report. There are those, but it's like the nature of someone, and there are those who are not so social.... Yeah, quiet person.
	(Nurse) One, they're not team players. How, like you cannot organize like you know, when you, you enter a shift to make work easier… you all organize yourselves...So you might find him or her excluding herself, she or he doesn't want to play together along, she's doing her things alone....
	**EM20: If certain cadres/ students are not members of a WhatsApp group (C) they will feel excluded from other members of the team (M) and miss out on information dissemination and this form of participation (O).**
	(Nutritionist) ...If the, at times we do feel like, you see the NBU team, they have a common WhatsApp group... For the nurses, and sometimes even for the doctors, they are both, they are both there both of them. But for us, we've not been added so far, so most of the time we find our communication through another party.
	(Clinical Officer intern) The [WhatsApp] groups that I am in...they are not...have no newborn issues …its quite quite… they just talk generally other things medicine.....The new born I mean specifically on new born no but concerning the other departments yeah
	**EM21: When the unit has a WhatsApp group (C) contributions may be skewed to certain staff (M) leading to dominance of some and disengagement of others (O).**
	(Nurse) . .....Probably they're just those [referring to other people] silent readers..... Yeah. If it's something that would affect us and the patient care, it's, it's important to always mention.
	(Nurse) Well, you see if, for example, like when you post something on WhatsApp, do you know not everyone will read, some people will just pass the information. It's not like comparing like when someone goes there and they are taught in person.
	(Nurse) .. They do more, though most of them are mute, most of them even if they are asked questions, especially most of the time we use WhatsApp, our WhatsApp group, you find that any matter arising whenever we discuss, some just don't answer. They don't contribute anything yet when you check, everybody is seeing whatever you're talking but somebody just decides not to, to

	contribute.... Well, you get to understand because you never know why somebody is not answering, maybe, maybe they, maybe she has had an experience before of the same problem and it was not tackled. Yeah, and some maybe they fear to share out to their opinions because they don't know how it will be handled.
	**EM22: If staff do not feel a sense of belonging (C) they will tend to work independently from others (M) increasing personal control over their work, but further isolating themselves (O).**
	(Nurse) I don't bother now..... I do my shift, I do the best I can do, I go home.
	(Resident) Okay, for the nurses, content basically was just maybe supplies. Maybe, "I can't find this in my room. Can I get this? Or maybe if the patients," you know, maybe I want to communicate to the mother, "Help me this." It was mostly ward-work based? I don't think we ever went extra with the nurses. Yeah, it was just patient centred.
	(Nurse) .... I don't know why I think like everything I try to propose to some people,... it can't work so that you don't look like you're trying to, to lead the way or something... that's my feeling
	**EM23: When staff are not included due to other, e.g. lower social status (based on cadre, junior role etc.) (C) they may feel a sense of reduced belonging and may disengage (M) and give limited contributions to care, with lower satisfaction (O).**
	(Student Nurse) With the doctors, I was feeling free to ask, especially the two I've mentioned, but this one also comes with the doctors, because I realized there were certain procedures that we could help. Actually, we could perform well than even the doctors, but they're afraid to approach the students. Like the, all they wanted was like, is there a nurse? Are you with the nurse there? Who are you with? You get?
	(Consultant) I think it is culture. I sometimes feel we don't engage them enough in the ward-round, to feel useful. For instance, in my ideal world, I would like to see a baby, listen to the presentation (from the interns) and listen from the nurse…like actually hear from the nurse: What is your feel managing this baby? Many times, the nurse is the silent bystander… and within 15 mins they (nurses) disappear on to do other things where they feel more visible.
	(Nutritionist) Sometimes if you are invited, but sometimes you see their meetings are for nurses
	(Student Nurse) I feel ward rounds now there have been left for doctors in the hospitals. And I feel they have left one part of two people who are very important. They have left out the nutrition, they left out anything about nutrition and we have left the out nurses, this nurse has stayed with this patient for 24 hours. You have to come there for 15 minutes. I think it is good to ask me how your patient was before even going to hand it over to a registrar who came in the morning, did the ABGA very fast and sat down to write the summary. They have not even checked on the patient, but that nurse has a lot of information about the patient, but she will keep quiet because she is not asked.
	**EM24: When specific cadres/ students not invited or invited too late to CMEs or are excluded from communication (C) there will be reduced sense of belonging (O) as they have missed out on interactions and information (M).**
	(Student Nurse) okay, there are normally CMEs but I don't know why, I've never seen students going.... I've never seen students going as opposed to other hospitals where I've been, these CMEs, was mandatory
	(Nutritionist) I've only attended two CMEs, that we were invited to attend...Sometimes you don't know even.
	(Nurse)...... Late communication, is always communicated like, you'll find that we have a CME tomorrow, so to factor in in my plans is sometimes difficult. It's always like impromptu. It's not something that is planned like next week on this this day we'll have a CME on this and this topic. So, if I'm just told there is this CME and you don't even know the topic, yeah, it becomes difficult even to plan for it.
	(Nurse) I have never attended. You see we usually, we are usually, it's the in charge who releases people.
	(Senior Nurse) And when seminars come you allow, you don't repeat the same people.... You don't repeat the same. So they get motivated at least, a certain person went, another one has gone, and another, unlike a situation whereby it's certain people who go. So, that one also motivates them. I think it's that.
	**EM25: When individuals are unable to attend CMEs, due to workload of poor administration of meetings (C) updates will not be effectively disseminated (M) leading to lack of continuity and reduced sense of belonging (O).**
	(Nurse) .....Late communication, is always communicated like, you'll find that we have a CME tomorrow, so to factor in in my plans is sometimes difficult. It's always like impromptu. It's not something that is planned like next week on this this day we'll have a CME on this and this topic. So, if I'm just told there is this CME and you don't even know the topic, yeah, it becomes difficult even to plan for it.
	(Nurse) Mostly Wednesdays as from 12:30 to around 2:00.... But according to our shifts, maybe sometimes on Wednesday you're on off duty so sacrificing coming for the CMA it's hard... Yeah. So if you're around you attend, if you're not around it passes.....And also most of the time, most of the time, that time at around 12:30, you're handing over....So after handing over it's around 1:30. So if you go to the CME, maybe they are halfway or also you feel, I'm hungry let me just go home, I'm tired also.
	(Nurse) Will you see like, if they are organizing any let's say CMEs, and you find yourself you are few in that unit, most of the time it's postponed. So, we end up not attending. If not postponed now because you are, if we are few, of course, we'll not attend, we will not attend. So, most of the time we don't get it.... We miss out, yeah

**Roles clear and aligned**	**EM26: When staff are clearly delegated specific roles (C) they will be able to build communication ties with necessary colleagues (including external staff) for their role (O) because they have the delegated authority to do so (M).**
	(Senior Nurse) Supervision, eh, it's a responsibility of all of us, and far more the team leader or the ward manager. And you see, you can't be able to supervise alone or to manage alone. So I have identified mostly senior nurses who have experience in the unit. And have delegated. Delegated, because since I can't be able to do, yes, I have delegated some of them. I tell you, you are going to be in charge of consumables. Ensure when they come from the store, you check the (ordering form), confirm. Ensure they have put well, and they will be entered in their consumable book. And ensure, be checking sensitize people, when somebody picks things from the store, has to document somewhere. Then I, I delegate somebody. Your work, you will be going able to check whether vital observations are being done. So it's like targets have given people.
	(Nurse) So, we normally have circulars. So if a circular is written, it is directed to the ward in-charge. So the ward in-charge has the responsibility to disseminate information to the people on the ground the nurses in the unit. Obviously, it comes up to down. The same way if you have an issue in the unit, you have to relay to the ward in-charge the ward in-charge relays to the senior assistant chief nurse is the one who is covering the department from here it goes to the one who is covering the post as director of nursing services. ......So something emanates from the nurses it goes to the team leader. So if the team leader is unable to handle, transfers to the ward in-charge, so from the ward in-charge, the same channel, to the senior assistant nurse who is covering the department. The senior assistant nurse now relays to the main nurse.
	**EM27: When individual roles are clear (C) others will more readily identify go-to people (M) leading to more effective communication (O).**
	(Nurse) Yeah, something like that. That one is also applicable. Maybe I feel like I, I need some time off to do my other things, maybe I have an exam and I need some time. I'll go directly to the in charge and explain to her my situation, and how she, how she, how she may help me.
	(Nutritionist) Okay, it will depend with the situation I am in. So like, if a baby doesn't have a feeding tube, the first person I look for is a nurse. Yeah, that is the first person I'll run to. I'll let her know, the baby is supposed to feed but doesn't have a tube, so she can fix, he or she can fix it. And if we've calculated a parenteral nutrition and a baby doesn't have a line, I'll look for the doctor who is in that room, so that she or he can assist to fix the line so that the baby can receive the parental nutrition now.
	**EM28: When staff are allocated (by others or self-allocation) to certain specific tasks on the unit, e.g. equipment, medication (C) this can become their boundaried scope of work, as seen by self and others (O) as they increase skills in this task and deskill in more general nursing (O).**
	(Student Nurse) Another person I would prefer to be on duty with X because you see without sufficient functioning of these equipment, it might be very difficult for you to operate. ……So after every hour, you go, go around, you take the vital signs of all the babies. But you see, it depends with the efficiency of this SPO2 machine. So, I could see X every morning, he would service them, he would clean them like every morning, every day. So you see is like what he's doing he's helping us also do our work efficiently. Yes.... Things move smoothly, very quickly.
	(Nurse) Well, I communicate with almost all professionals within our unit …… because of the issue of equipment, it touches around almost every corner. The content of my communication with them is based on effective use of the machines, care of them, preparations and disinfection of all those machine parts.
	**EM29: When staff have a clear role but fail to perform their role adequately (C) others will feel aggrieved (O) because they are not contributing sufficiently to overall function and performance of the unit (M).**
	(Nurse) So there are some people who take to…to delay by time like no, let's do this when you know it's not supposed to be…like that way. So you keep on arguing arguing for the sake of the patient and at the end of it you feel like …what are we doing here?
	(Nurse) Mostly they are these interns, the MO interns, they're very green, so you will find that as much as you're with them on duty, you are the one doing most of their jobs. You are the one, in case of an emergency, you are resuscitating, you're putting the lines, you're putting the IV fluids. So, you find at the end of the shift, you are so fatigued.
	(Nurse) and I know my in-charge can't listen to me on any issue.... yes, there's always like a …l fight.I.... I can't, I wouldn't insubordinate my in-charge.... yes. Or it has to be through her.... yeah. so, I stopped. I don't say nothing.
	**EM30: If a staff member considers a task should be undertaken by a different cadre (C) they may refuse to do it (O) as they consider it not to be their role (M).**
	(Senior Nurse) Yes might not be willing because some…there are some procedures somebody feels this is not mine.... You know there as a nurse you can even do extended work. You can do some work for the doctors so some feel this is not my part… it's for the other person so they are not willing to do it.
	(Nurse) And they're supposed to deliver because, okay. Some of the complications in labour ward, like maybe shoulder dystocia or they are supposed to, like if we, they go into ward and the nurses are there and the nurses are not able to do like the delivery of bridge or shoulder dystocia, they're supposed to chip in because they're the senior.....Most time they see a bridge, they disappear.... Yeah. Even during the resuscitation of the baby, it's always the nurses who does that....Probably lack of confidence. Yeah. And, Okay, another thing is I think they have this mentality that delivery belongs to the nurses.

	(Nurse) Maybe when I am a team leader. When you tell someone to do something... like Maybe you can ask them can you please go to the pharmacy?... maybe it was my work to go to the pharmacy but I feel I'm overwhelmed with my work so I request somebody ...can you please go for the drugs. You see they will be like that's your work..... Yes...but not all they are few, in fact, most of them agree and they don't refuse.
	**EM31: When a staff member prefers a specific type of role (e.g. practical vs documentation) (C) they may informally assign less desirable roles to colleagues (M) leading to piecemeal (and gendered?) delivery of care, poor quality documentation, and deskilling (O).**
	(Nurse) There are people here who don't like writing, like for men, men don't like sitting down and writing..... So when you know you are, you are having a man, that man will tend to do most of the manual works, deliveries, what, and you will be the one to be writing.... So it's easier for me that way, when I work it that way. When I enter I know, this one loves this and this but doesn't like this part of work, so I will go do the part that she or he doesn't love and the person works where she or he likes. So it's easier, so you don't even disturb each other.
	(Nurse) So, okay, me I'm, me personally, I'm a kind of person that loves to manually do things.... Yeah, hands on.... Yeah. So even if I'm doing a procedure, most of the time someone will tell me, (name omitted), I'm tired of writing, come and write also, then I tell the person, you just continue writing. So, I don't prefer writing so much, I prefer working......Mostly you know females here are many, so most of the females' love writing, they don't take it so… [laughter]... For the men. Most men love task, they don't like writing.
	(Senior Nurse) Okay. Let's say now like right now we have, our numbers have gone up, maybe, like yesterday we had seven turns, you have somebody who just comes how, he wants only to do, to do documentation and not hands on. Just to sit down and document. Maybe now as a X you have to do other managerial work. Maybe let me say that very day the in charge is not around or she's gone for meetings, and you're alone there with the same staff who like sitting.... She doesn't know many procedures. Now you have to do all those things, before you start managerial work maybe it will be around three, you leave here at six or seven-ish?
	**EM32: When a member of staff with a specific role is absent (C) work will be difficult for the wider team (O) if there is nobody else who can perform that role (M).**
	(Nurse) So maybe, you know, there's, there's a specific person dealing with specific things. And they're not around. They don't do not want to disturb them when they're away. So such time is you may miss the information....
	(Nurse) For example, now we are with the MO we are doing night, decision making, this neonate you need to do this and this, even me now I'm stuck. So now, that's when now you call the MO maybe the MO is not taking your calls, you see now you are stuck.... You just leave it till the morning, yeah.
	(Student Nurse) For the doctors, they don't pick calls. And by just displaying who is, who is on call can also help. Sometimes it's hard to look for them, the hardcopy stuff; sometimes they don't pin them, you have to do request, "Who is on call," vitu kama hizo [things like that], yeah.
	**EM33: If staff feel underutilised in their skillset (C) they will feel less satisfied and will deskill (O) as they cannot be their true self/ exercise their skills (M).**
	(Nurse) To some extent because I feel like I'm underutilized because I'm a critical care nurse, I need the skills, so I think I'm not satisfied with that.... So if you get somewhere where you can utilize your skills, you feel more satisfied with your work than, like for me in the postnatal I feel like being wasted because I'm not practicing my skills.
	(Student Nurse) I am empowered to do something there. Probably now like there are procedures that I am empowered to do as a nurse. When you come to these institutions, they have their way of doing things. This should be done by only the consultant or the medical team.... So, you cannot do it. Yes. You have the skill and the knowledge to do it... But despite having the skill and the knowledge you are limited.
	**EM34: When a member of staff with a clear role has too many completing tasks (C) they will adapt implementation of role based on prioritisation of tasks (M) leading to omission of less urgent tasks, e.g. attendance at ward round (O).**
	(Senior Nurse) Ward rounds are usually done on Tuesdays and Thursday, but sometimes it's a challenge for, for especially as team leaders to join. But I always encourage the primary nurse to ensure he or she joins the round. But me once, I'll just go attend maybe one or two babies. Because that is the time, you have other responsibility. You are expected to order to go orders, to prepare duties to go for the meeting. You are being called here and there. So I can't be able to attend all the rounds
	(Nurse)... Three would be enough because if you see right now, in X...X there are 14 babies at the moment. The nurse is one...the nurse is feeding half of the babies...nine babies. You, see? That same nurse is feeding those 9 babies. That same nurse will do vitals, that same nurse will write the kardex, that same nurse will attend the round.... That same nurse will do top daily. Maybe that same nurse maybe the doctor will say, I want us to do this procedure... will do what?
	(Senior Nurse) ….. we have very, a few staffs who cannot attend to those patients. Now you cannot just locate yourself that you are doing managerial work.
	(senior Nurse) And in fact if you go there you will not, you will not imagine she's an in charge. You will find you imagine that is a, a, just a normal nurse because she does more of the care than the managerial work.
	**EM35: When individual roles are unclear (C) responsibility of individuals also becomes unclear (M) leading to evolution and duplication of roles, conflict, ineffective handovers, inappropriate delegation to students, non-completion of tasks and risk of patient harm (O).**

	(Nutrition Intern) so, I found this lady, out of her, out of her goodwill of her heart, she was helping feed the baby. But the way she was feeding, from a far, I was about to die coz I was like, that baby is just gonna aspirate. So, I just went to her, and, and, and, and told her, what she was doing was not correct, but thanks for helping either way, so, I did try to be as polite as I could, while still feeling very... yeah, as if I want to punch her.....oh she was like, oh, sikuwa najua (I didn't know) but now I know.
	(Senior Nurse) …….., they even do the work that is not, okay, they even cross the board because they, okay, they'll even do cannulations. ... Yes, they do more than nursing....
	**EM36: When a role is perceived to be unfilled in the unit (even if someone formally has the responsibility for the role) (C) others may step in to informally fill the role (O) due to personal interest/ambition/attitude (M1) or sense of duty (M2).**
	(Nurse) Mostly they are these interns, the MO interns, they're very green, so you will find that as much as you're with them on duty, you are the one doing most of their jobs. You are the one, in case of an emergency, you are resuscitating, you're putting the lines, you're putting the IV fluids. So, you find at the end of the shift, you are so fatigued.
	(Senior Nurse).... She doesn't know many procedures. Now you have to do all those things, before you start managerial work maybe it will be around three, you leave here at six or seven-ish?.. And you know you, you cannot be telling an adult to do this and she knows what she's supposed to do.. And she doesn't want also to know... So it becomes difficult.
	(Medical Officer) …he assists us like he doesn't take it like oh I'm just a X I'm not going to. He helps us with lines (canulation)... He helps us talk to mothers who we can't sometimes get through to.... So like I said, it's like teamwork…... So actually he's one of the people we… him plus his team
	**EM37: When uniforms are consistent, e.g. same cadre in same uniform (C) it is easier to identify staff by professional role (M) improving role clarity as well as security (O).**
	Observations
	**EM38: When uniforms and ID badges are not worn consistently by staff (C) it is difficult to identify people on the unit (M) and incorrect assumptions may be made (e.g. male nurses in white dust coats) and risk of non-identification of strangers (O).**
	Observations
	**EM39: When clinical staff are not formally/ clearly delegated a teaching role (C) they will not see this as part of their job (M) leading to poor student and staff experience (O).**
	(Nurse) So, that now you find there are very few people who can teach those students, like X nurse now. So now, X nurse can teach especially. Yes. Once it's daytime, she can teach these students, they are supposed to be learning. But others they say, it's not their work. They are supposed to be taught by the mentor, because this student mentor is being paid some money from those schools. Yeah, that's the thing now. So, some people say, "Me I can't teach because nobody's going to pay me." Now, you see
	(Nurse) you know the whole hospital, it's only one person who's selected to mentor all these students in this unit.. in all this unit and yeah. So, you can imagine...the, I don't know whether it's the work of X and Y to mentor these students,... I think we just do it out of, out of love and out of, you see?. out of just helping. Because I don't think that is, that is their work.... because I know there's a student mentor who is supposed to come and mentor those students, every day, on what we are doing. But we teach them!..we just teach them. Yeah, we mentor them.....regardless.
	(Nurse) ideally, the students are supposed to be mentored by the clinical instructor appointed by the hospital..... but on our setup, mostly they are just deployed to the unit, so it takes an individual interest, either to share, if you want to share knowledge with the student so that whenever they qualify... atleast they are competent enough to, to also discharge care coz, we always try, we put ourselves like heh, in the next three years... you can be, you can also be admitted, and the same same person, you didn't mentor, is the one taking care of you... that's why we tend to just share information and try to mentor them.
	**EM40: Allied health professionals may selectively attend the WR (O) due to competing pressures (C) and prioritisation of tasks (M).**
	(Nutritionist) Yes, I do. Not all of them, because they're usually they can all run parallel at the same time. So what you do, you just time the baby that you have interest in, that's when you keep dashing into either round.
	(Nutritionist) Every ward round if it's there, mostly on Tuesdays and Thursdays...... No, I attend for the premature babies.... Yeah. Then my colleague… attends for the NICU.
	**EM41: Where nurse workload is overwhelming (C) they will not attend the WR (O) as they fear abandoning the babies (M).**
	(Nurse) It could be the workload. There could be alone like in our HDU. Sometimes you find one nurse with even 20-25 babies. It is sometimes impossible to attend ward rounds, depending on the workload that you have.
	(Nurse) Yeah. But let's say not often because of the workload. You find you are in a third-room; you are the only nurse. You are nursing 10 and more babies who need a lot from you. So, you don't abandon the baby to attend that round. You just work with the doctors because the care of these babies will be compromised. That's why sometimes if you have more or less, you can follow them and attend. Sometimes you are in the room, they are doing their... they are talking, you are listening. So, you are attending to this patient but you are also listening what they are doing.

	(Nurse) Yes, we are supposed to attend but it is a different situation. Like if it started and maybe the procedure that you are doing, like you are putting up IV fluids. You know there are things which you cannot postpone to go for the ward round, I'm going to put this baby on IV fluids, I'm going to put the baby on IV fluids, then by then we finish the ward round, the baby has dehydration. So you wait as your room, as they call you in your room. If you are not busy, you show up.
	**EM42: If a member of staff does not wish to expose incompetence (C) they may alter their work (O) to avoid the specific procedure, for example (M).**
	(Student Nurse) Any other thing, most of the time they are… they use… they criticise you like why don't you know and you are in third year?... I'm like wah, that one it's a discouragement on its own so, yeah and also most of… most of the time they live you with the work..... Not all the work but… like when you are doing the work and you find you don't know you fear asking, the fear so you just think or you just walk away slowly and ask another person then you come and do it.
	(Senior Nurse) ...Yeah. Towards work towards some specific I mean you know there is Somebody everybody has a weakness somewhere. so maybe I don't know, like setting a CPAP somebody tells you, no this one maybe doesn't need CPAP because he or she is trying to avoid it.... Maybe trying to avoid it you can also know whether she knows or she…she doesn't have knowledge about setting the CPAP for example or they don't want to put it.....Lack of knowledge also... So you know you try and avoid it.

LEADING

**Setting positive values tone**	**LD1: When leaders set a positive 'values-tone' on the unit, e.g. listening to juniors, kindness, civility, good leadership practices, praise (C) staff are encouraged to 'follow-suit' (M) leading to a more organised, positive and respectful place to work (O).**
	(Nurse) Yeah, if you find leaders who are really guided by the values, it'll really impact on what you do. So maybe you find leaders who embrace teamwork. You will also follow in the same line. Teamwork.
	(Student Nurse) Yes. But another thing that came very clear was the leadership, the in charge. The leadership sometimes, very early in the morning, she's there, you come and you find her and you're like, then tomorrow I have to… I have to come a bit earlier so that she finds me here. And then the in charge, you'd, you'd, you'd, you'd, you'd hear like give information to the nurses, more information, in a very polite manner. Yeah, the one that everybody agrees with. And she would also inquire from different people and then decides on just one thing to be to be done and everybody picks it. So… The leadership style of the in charge is also, is also nice, yeah.
	(Nurse) I, okay. The, I can say their leadership skills to me, to me, they were good. As in, they were role models to me. Yes. The way they handled things, the way they brought people together. The way they worked with people, some of them even challenged us to, to further our studies. Yes.
	(Resident) I think people, yes. The workers, the in charges, they have done so many work that so that NBU can be organized and NBU can be a better place.... I think yes, the leadership, yes.
	**LD2: When leaders display admirable characteristics, e.g. further study, good work ethic, patient-centredness, collaboration, professional behaviour/ attitudes, mastery of clinical work (C) staff will emulate these (O) as they are encouraged/ feel empowered when these qualities are shown by someone in a senior position/ leader who they respect (M).**
	(Medical Officer Intern) patient care affects her directly on a very personal level. When we lose a child, she, you can actually see she has personally lost something, you know... yeah. It's not just doctor-patient relationship, she…. yeah, she is really invested in what is going on in the unit, she really wants to make things better, she really wants to make things work, she really likes seeing babies come up, yeah, she is very passionate about whatever she does.
	(Nurse) The senior, the senior colleagues. You find that someone handles the, if, if a case comes, someone handles it perfectly. So maybe if I'm improve on my knowledge or I improve on my studies I think may, I may be somewhere.
	(Clinical Officer Intern) She was very dedicated...she would come even during the weekends even during the odd hours. And then the way we interacted it was not like boss-servant…
	**LD3: When leaders display an open attitude to learning (C) staff will feel able to correct a leader (O) because they feel empowered by the leader to do this (M).**
	(Nurse) Yes, because somebody can tell you, you should have done it like this or this like maybe they tell you we need to have two nurses on this one can work on the patients with X and the other will work on the patients who are not X...it changes me because I have to look for an extra nurse to work in that room at least I have seen now it is giving an impact.
	(Nurse) MBT: Yeah, very much. There is. Like now there is a time the MO can hold her, the patient needs a CPAP, you can just go there and access with her or him and you can change everything and you can listen, she can listen and change. Or concerning medication, cannot one tell you, tell her, "I see this one is supposed to be one, two, three. She can change.
	(Nurse) Maybe now you know something and you are sure you know, but here comes your senior, like the MOs and the whatever, the consultants, you try to tell them these things is supposed to be like but they think that they know much better and they don't believe that you know. So, they can say, "No, that thing is done like this and this." So, maybe you're not given chance to apply. So, that's the thing.
	**LD4: When leaders set a negative 'values-tone' on the unit, e.g. blame, incivility, overwhelm (C) staff will lack an authoritative positive example (M) leading to normalisation of undesirable staff behaviours (O).**

	(Nurse) It's not bad. Okay, depending on which, which if it's my immediate supervisor, she's normally patient so she's advising or supervising us there, but sometimes when overwhelmed (laughs), you can really be a dictator, she can be, she can be harsh.
	(Consultant) .. saying that very carefully, studying your face, because once the creased lines appear, she will not give you the details..... That you are going to disapprove of what they did.
	(Senior Nurse) Sometimes you have to be harsh on them and tough on them because you warn them if you don't do it you have to document why you have not done it.
	**LD5: When staff are not supervised by leaders (C) they will feel unsupported (M) leading to reduced satisfaction (O).**
	(Nurse) from the nursing service manager, we've got unit-in-charge. So, the unit-in-charge is the one who has been mandated with the supervisory role, in that, mostly... it's not on patient care, it's just on reporting.... as in reporting is, have you come on duty or not?.. it ends there, it's just limited to whether you've come or not...but going down to either you're doing the right thing, what I can say, there's no supervision.... it's more about, like, your presence at work.... support, there's no support...
	(Nurse) I... to be supervised, I don't think
	(Nurse) Supervision, it's a long time since I saw it. Isn't it about the seniors coming?.. It has been long since I saw it here... It has been long.
	**LD6: When leaders are blaming, policing, unappreciative, humiliation in front of mothers, demoralising (C) staff/ students feel discouraged/ fearful/ aggrieved (M) leading to erosion of self-confidence and 'othering' (O).**
	(Nurse) The other one is maybe somebody sees you've done something that's not supposed to be done on, or in a way that's not supposed to be done. So instead of waiting, maybe it's feeding time, mothers are feeding they'll come and talk it out too loud, and everybody's around. …… it's good you intervene immediately, but not in a way that makes the other person feel as if they are so junior. So maybe you can show me how to do it, and when the mothers have left, or we are the two of us, and you know, show me, tell me if you, you feel like I was ignorant, you can now really talk to me when we are two of us. You feel like, now you, you feel the need to change. But you now you've seen me doing something and you just bring it out so loudly in front of everybody.....
	(Nurse) ....Hmm. I don't feel it's supportive supervision. I feel it's policing supervision. .... Policing supervision is like, don't do this, don't do this. The don'ts, are many than what you should be doing, and what you are being told, I feel if you don't have this, do this, if you have this, I feel you should add on to this, but it's like, don't do this, don't do this. So you are working like, as if you are, you are afraid what happens. Will it be now, if something happens, it'll be me. I'm the one who's, who's accountable.
	(Senior Nurse) Yes negative one is when you've done something and people don't see.... yes, you are not appreciated... Lack of appreciation and then…. Everywhere all over from even the mother it can come from the client, the staff, the administration. …all directions because maybe you've tried your best anyway then somebody comes and says you know you have messed up you could have done this this the baby could have survived this you know, and if personally… your best, you tried your best. So that is what demotivates.
	**LD7: When leaders display norms of practice (C) others will follow (O) as they respect their judgement and position of authority (M).**
	(Resident) Mostly once you check in with the fellow, she'll be able to guide you and tell you, "This is the practice that we use, or this was what was used zamani [long ago], we don't use it anymore," you know?
	(Nurse) I'll say the seniors, and those you, you communicate to mostly. Those who feel free with mostly. So if seniors are doing things a certain way, you will feel the need to follow in their way, if they're reluctant you'll be reluctant. Cause if maybe you may feel they are, they know something that you don't, they may have experienced something in the past, you know they've been here for long. They may have certain experiences with certain ways of doing things.
	**LD8: When errors/omissions are immediately taken up with an individual (C) this issue can be quickly resolved and learning gained (M) leading to better care and improved team working (O).**
	(Nurse) If you say what is right at the right time, it will help so that the mistakes should not be repeated or the procedure should not be repeated. You correct it and you do the right way and it will help the patient not to long stay in the hospital
	(Senior Nurse) I get hold of the person plus the mess or the document. We come and sit here and ask, this is what is happening today that this is what happened, the other day when you were there, is there a problem?....... And I still do the monitoring and I always after if they improve I always even at the desk say, so and so has really improved. Let's clap for that person.
	(Nurse) Okay. I tell you as a person you have done this and this, it's alright. Next time, do better. And I'll tell you how to do it. I'm not going to tell you to do better, if I'm not telling you the right way to do it. I tell them, if I've done something which is not right, even if I'm your senior, tell me. Don't fear and say this is my senior. No, we work for the betterment of our outcome, of our patients. If we are shying off each other, then we don't expect a good outcome for our patients.
	**LD9: When a leader communicates clear consistent expectations for staff behaviours on the unit, e.g. punctuality, attendance at handover (C) these are more likely to be upheld (O) as all staff are aware of expectations and will fear consequences of deviance (M).**
	(Consultant) Yeah, there is this baby who is dirty. I will say it twice. If you're not going to clean, I'm going to clean myself..... Then you will see the outcome. So the next time you see the baby is dirty. You probably do it coz you know if you don't do it, I will do it.......

	(Nurse) People come here ready to work. You see, it's not like you know other wards I… where I've worked in ward where by you go, you receive a general report …and then you wait to be allocated..... Here we come when we are already allocated and you go and you start your work then there is so much routine here, mothers coming every 3 hours it keeps us on our toes.... I must have done this before they come.
	(Student Nurse) We would be called in a room, if it specifically for the students… Yes, we are called in a room and we are given the advice and the motivation. We are told first you did this and you are supposed to be doing this. Then after that you are told…
	**LD10: When there is a legacy of poor reinforcement of standards by a leader (+/- no processes for reporting) (C) digressive behaviour will be normalised (M) leading to a poor working environment for all, sense of futility for leader, and frustration by others who do not have necessary power to intervene (O).**
	(Senior Nurse)..... She doesn't know many procedures. Now you have to do all those things, before you start managerial work maybe it will be around three, you leave here at six or seven-ish?......And you know you, you cannot be telling an adult to do this and she knows what she's supposed to do.... And she doesn't want also to know.... So it becomes difficult..... And they're rigid to change.....And they're just timely. Somebody just want to, okay, maybe now you, she reported in the morning and is supposed to leave at 12:30, once 12:30 reaches, she doesn't know the procedures. [laughter]. Where the, where she had reached for document, that's where she will stop.....Yeah. You know now this nursing whoever said is handing over is, because now somebody says, I now want to handle over all the procedures I had not done, but she was just sitting.
	(Student Nurse) Yeah. You're forwarded to the, to the, it's called what? To the sixth floor, the nursing standards..... She was, okay... the, the reason to that she was giving excuses. Sometimes they weren't making sense, but, to some extent they were being believed. [Laugh]. Yeah.Let me say, it was just luck.
	(Health worker) mmmh, (Pause). Why does that one person keep coming into my head? Like I said, they are very chauvinistic, ...yes, it's the same person I described. I feel like, their people skills, you know, how they interact with people is, really missing.....it's in absentia, it's absent. Out of…. it's not, coz like I felt very…., it's so sad coz it's just didn't happen to me, it happened to another colleague of mine....... I don't know what we are supposed to do! Cause like the people that we would have approached, you know, with this behaviour, are the ones who are present when these behaviours are happening. When these words are being said, they're present in there, and they are like, and they just laugh it out. So, you are like, okay. I guess that's the new normal, and you just move on.....
	**LD11: When a grievance is met with perceived inaction (C) futility and lack of trust (M) will lead to frustrated staff and workarounds (O).**
	(Nurse) I don't know. Because nobody will listen to me. Am not the in-charge, right?...so, I have to raise any issue through my in-charge.I... and I know my in-charge can't listen to me on any issue.... yes, there's always like a kasmall fight...... I can't, I wouldn't insubordinate my in-charge..... yeah. so, I stopped. I don't say nothing....I felt bad those initial days, but I got used to it... I don't bother now... I do my shift, I do the best I can do, I go home.
	(Nurse) ... It did not happen. So, now you just put them in the photo, you do everything and just keep quiet.... And just, we just pin them there and there in the morning or in the evening when I'm coming for night, "You didn't do that and that because of that, that one has happened. So, you see
	(Nurse) I don't think it is well received by top managers..... It does not really affect me personally. But when you look at the goal, the ultimate goal or the broad objective, it somehow affects us because you see that there are so much that is limited, there resources are limited, you are understaffed and when you communicate, that is not taken care of. So, I think it can improve. If there were listening to what we say or our opinions, I think that can improve the quality of care and it can even do much better.
	**LD12: When handover/ ward meeting has clear expectations and is respected by staff (C) it enhances belonging, freedom, collaboration, flattening of hierarchy, and sense of community (O) due to inclusion of staff in a reliable common forum with opportunity to contribute, raise any issues, and be updated (M).**
	(Nurse) Handover meetings. OK, the same meetings. We normally have a handover and then we have other things in the meetings after the handover meetings. So, it is a mixture of everything, as in, you can hand over, you can share information, you can teach during the same meeting
	(Nurse) Okay meetings so if we have a topic that we have to sensitize others, so we talk the person who has information prepares and comes to give us the information like CME. But if there is a letter from the administration or management somebody comes and reads the letter the way it is at the meeting so sensitization happens like CME alafu (OR) another type of communication is when one tries to raise something in the meeting like complaining or affirming, we will be told who wants to say something they pass the information... they give us......WhatsApp we communicate..... Like people whose licenses have expired they are being told... if there is a letter that it has come to the office, we tell the people kwa WhatsApp yake (on their WhatsApp) generally we are informing people there is shortage of this drug please do not bother to check on it......Supplies, if there is a meeting notifying people on the meeting like mortality meeting for CME WhatsApp is for informing...
	Observations
	**LD13: Structured and respectful handovers (C) are respected by other cadres (O) as they recognise the value for patient care (M).**
	Observations
	**LD14: When leaders reinforce structured and respectful handover (C) latecomers will behave humbly (O) because their lapse has been noticed (M).**

	Observation
	**LD15: When there is clear expectation about the content and format of handover (C) staff will recognise it as an essential part of the job (M) and it will become the norm (O).**
	(Nurse) Handovers. Starting off with definitely there is a, the way we do it. The way you mentioned the, the name, you confirm the name tag, the IP numbers, the condition of the baby and the current management so far. Let me mention on the most sick baby, probably the baby is on IV fluids, probably not feeding, you just go mentioning the, the details on the current plan recommended by the doctors. So that when that nurse comes and start the nursing care from where they, you stopped, it'll be easy for them too, to know what next, what to do. And most times if it's a very sick case, you'll find that it's easy even referring to the file. Cause you know, where that's where the doctor's notes are. So that you can see the current review and the current plan. And then from there, you are able to pick up, oh, okay, this this is what we are supposed to do, and how the baby is and then you carry on with the plan. .....
	(Nutritionist) When you are going for a break or, for leave, we have an official handing over form. Which is an evidence that you handed over to someone. The person signs and you write all the patients that you think need extra follow up.
	(Student Nurse) Knowledge? It's shared mostly in the, in the, in the giving the reports. They, they normally discuss about issues and upcoming trends and maybe things that they should adopt. .... It's during the handover. They usually meet on Monday, Wednesday and Friday, in the morning.... About maybe upcoming issues and also when new devices have been brought, like for the people of NEST, they only come and educate them. And also passing of information probably about the patient. The progress of the patient is given by via report.
	**LD16: When a leader proactively manages the online WhatsApp space on the unit (C) content can be organised, informative and inclusive (M) and foster belonging and awareness of staff/ students (O).**
	(Senior Nurse) WhatsApp we also share our duties after I've organized the duties, the duty rotor is ready. We take a copy of photos of the duty rotor, and send them on the WhatsApp, so that those ones who are off, they don't need to call or to come to check their duties. Eh, WhatsApp also is also a way of communication for issues of us in newborn unit. Like right now we have two of our colleagues who lost a, grandmother, who lost her sister who are the guardians. So such we communicate on the wall so that we get people to release. .. It's like a social welfare
	(Resident) We have a leader; we have a team leader, everyone has a team leader. There is a nurse in charge who is a team leader, there's a doctor who is a team leader at certain things, there is a WhatsApp groups. So the team leader, if the senior communicate to the team leader, a team leader now disseminate the information through the WhatsApp cause you can sometimes you can't find everyone at the same time. If there's a WhatsApp group, you just continue saying, “This and this and this is what is required, this is what we have tomorrow, this is the meeting you will have tomorrow; this is what we do. There is a change in something, now that we klebsiella outbreak there is this thing that you need to sensitize and this we need to insist,” that's what happen.”
	(Resident) ..The team leader always communicates. Every second they're like, “Oh, you know, this has been said, do this. The rota is here for this week, do, these are the people.” So, it was a continuous communication, and we still are in the [WhatsApp] group.
	(Nurse) WhatsApp we communicate.... Like people whose licenses have expired they are being told... if there is a letter that it has come to the office, we tell the people kwa WhatsApp yake (on theirWhatsApp) generally we are informing people there is shortage of this drug please do not bother to check on it.... Supplies, if there is a meeting notifying people on the meeting like mortality meeting for CME WhatsApp is for informing
	**LD17: When the use of WhatsApp is widespread on the unit and within different subgroups (C) its role and administration/ governance may become fluid (M) potentially leading to safety issues (O).**
	(Medical Officer Intern) you post to the whatsapp group. ... you give a brief history, if you need to attach images of whatever the problem is, you attach images. If you need to attach results, or radio x-rays, or ultrasounds, whatever it is you attach and wait for your colleagues’ responses. There is always someone who knows something.... very useful...once in a while, like once in a month… I’ll start with the unit’s colleagues, I’ll also consult the MOs, if am seeing it’s like no one has a clear picture of what is going on, you just post it there
	(Nurse) ...we have a nurses worldwide, where people pose questions.... it can accommodate, let me confirm for you,I: is it nurses from all over the world?R: yeah, they are nurses from different countries..... so it has, it has a lot of people... it has 250.....yeah, 250 members....
	(Senior Nurse) ...they are always there you can even ask you can even just within the environment but even you can go as far as we have a forum. So sometimes even ask there who knows somebody who can advise for this?
	**LD18: When staff supervision focuses on clinical care, and is timely and supportive (C) it can improve staff belonging and patient care on the unit (O) because good practice is consistently championed (M).**<br>**LD19: When staff supervision does not focus on clinical care or is not timely/supportive (C) staff will feel unsupported in their practice (M) and delivery of care will not be consistent (O).**
	(Nurse) ... aaah, actually, it exists in indirect form. What I can say.... mostly, from the, from the organogram, of the institution,...from the nursing service manager, we’ve got unit-in-charge. So, the unit-in-charge is the one who has been mandated with the supervisory role, in that, mostly.... it’s not on patient care, it’s just on reporting..... as in reporting is, have you come on duty or not?... it ends there, it’s just limited to whether you’ve come or not.... but going down to either you’re doing the right thing, what I can say, there’s no supervision…. there’s no supervision on clinical care.... it’s more about, like, your presence at work.

	(Nurse) ...to be supervised, I don't think.... I don't think there is any.
	(Nurse) Supervision, it's a long time since I saw it. Isn't it about the seniors coming? It has been long since I saw it here.... It has been long.
	**LD20: When learning is valued/considered continuous (C) staff will feel freer to express and discuss and be open to correction (M) leading to better collaboration and teamwork, and changes to practice (O).**
	(Student Nurse) Actually, it a lot, it does a lot. Cause it matters in the attitude and how you're performing some tasks, if you are told, okay, this is not correct, you have to change and you must receive positively so that next time you can be corrected when you're doing something wrong. So, to me, I take it personal, I take it positively.
	(Resident) Yes. So the advice is sometimes is you may be doing what you call under dosing a patient or not giving the correct dosage, or maybe you have written a correct dose but you have not posted it in the treatment sheet. Now someone tells you, "You tend to forget to write on the, on the, on the work plan, but you have not transferred the information to the, to the treatment sheet, kindly do that. Don't forget it, just do it." Yes, you just take the advice, you put it the correct way.
	(Nurse) So, I remember, you've seen this senior nurse who has come here, (X), she told me, "(Own name), don't be stacking them together because when you stack them together, you will not decontaminate. So now you have to separate them."...It was not even in my mind that I have cleaned…
	(Nurse) Yes, I have that freedom. Because if something goes the wrong way, actually I don't hesitate, I measure it. And I don't… Especially in the meetings, you find something is going wrong and it involves everybody. In the meetings, you come and I have an issue and you correct it and they take it positively.
	(Clinical Officer Intern) So that's why they used to correct me a lot because I was a bit green.... So there was nothing… I used to take it positively… …I have landed in the NBU there is the standard of doing things there..... There is no way I have never said, no this way I take it negatively I always take it positively at the end of the day I was learning.
**Respected, self-confident**	**LD21: When a leader has self- confidence in undertaking their role (C) they will be more effective (O) as they will feel comfortable carrying and using their authority (M).**
	(Senior Nurse) ...Eh, because me having have worked in this unity for a longer time of almost 25 years in newborn unit, I know what goes on the ground. I know the staffs, eh, I can be able to tell you, this one is moody, this one is okay, this takes the responsibilities or the correction positively. So having known what goes on the ground, having known the staffs that I have, I'm able to stand, and say, this is what I want. This is what we should stop doing. And those sensitive things that I know how they react, those are the things I throw back to them and ask them, how better do you think, we can do so that themselves, they come up with the solutions. So to me, I have no issues of standing and saying, this is what we need to do or this is what we should do.
	**LD22: When a leader is respected by staff/students (C) they will be able to perform their role more effectively (O) as staff will make effort to support them/ follow their leadership/ show their respect (M).**
	(Student Nurse) A lot of weight. Okay. The one which I'll give a lot of weight, it's maybe, let me say for the nursing, eh, the, the nurse in charge, for example, she is the overall boss and knows of everything that is going about, so that coming to a specific decision, it has taken a lot of consultation, now it will have much weight than a nurse who is communicating about something. Yeah. So for the nurse in charge and the people who have, the higher administrative, I can take much consideration.
	(Nurse) Okay, most of the time like advice from my in-charges, yeah. Whenever they give directives or advice, and then you follow them accurately, it helps a lot. Yeah, it has helped me even improve my, my working.
	**LD23: When a leader feels undermined and lacks self-confidence (C) they may become disengaged (M1) and feel overwhelmed (M2) leading to a perceived leadership vacuum and associated degradation in respect from staff (O1), which will further demoralise the leader/ reduce self-confidence (O2).**
	(Senior Nurse) Yes. They prevent me if they don't cooperate.... Maybe it's something that needs teamwork... If they don't cooperate....You become demoralised
	(Senior Nurse) And you know you, you cannot be telling an adult to do this and she knows what she's supposed to do....And she doesn't want also to know... So it becomes difficult.
	(Nurse) ... I don't know why I think like everything I try to propose to some people... it can't work so that you don't look like you're trying to, to lead the way or something... that's my feeling
	**LD24: When a leader is not respected by staff (C) they will lack social authority (M) so they will be unable to perform their role effectively/ staff will not value /will resist their inputs (O).**
	(Senior Nurse) Yes, its not easy. You have to handle so many things at a go. Its not easy because you meet people of different types, different characters... so it's not easy.
	(Nurse) .. for me, I will not escalate. Personally, because, ... I know, nobody will listen to me.... I don't know. Because nobody will listen to me. Am not the in-charge, right?... so, I have to raise any issue through my in-charge.... and I know my in-charge can't listen to me on any issue.... yes, there's always like a kasmall fight.
	(Nurse) It, it affects because you see like when you put a consultant, you see consultant is the final decision. When the consultant makes that decision, may be even his or her decision is wrong, most of the people tend to side with him or her.... And you maybe you can remain there alone. So you see, the consultants, those MOs, when they make the decision, it doesn't matter to them whether it's right or wrong. What matters to them is that I am senior, I have made a decision, and so you have to follow it. Unless you find someone else, you now go to another consultant who will override that decision.

	**LD25: When staff respect their leader as insightful, showing concern for wellbeing, approachable, and working for their good (C) they will bring difficulties to them (O) as they feel safe (M).**
	(Nurse) That is one. And in any case that you may find that you are stuck somewhere suppose either administrative issue or maybe you have an issue with a mother here, when they're team leading, you can comfortably approach them, and they're ready to assist. Are ready to assist in the sense that they offer, they're able to come up with a problem solving solutions that are going to, to steer you to, to make a shift smooth. And also to help in terms of even maybe you have lacked something, supplies. They make connections here and there. Maybe call different departments should you lack something.
	(Nurse) The support. The support from the management. At least if you have something that needs to be corrected, they take it up and they also pack it, they pack up on you. They echo the same sentiment on what is supposed to be done right.... Yes, the in-charge. If you have identified something that needs to be corrected and bring it out, she takes it up. She makes sure that it is well articulated and well corrected. Also, the support from the consultants and also the support from the doctors and registrars.
	(Nurse) Yeah, also from the leadership there is. Because. when you go to the office (in charge's office) and you want something from there, yeah you receive support cause let's say you are on duty, let's say tomorrow you are on duty and something just came up, an emergency which is unavoidable. So, if you just approach the office. They'll just give you an OFF. Then they will know how to compensate for that day you were supposed to be on duty, they know. …..So, if you need anything, if you need support, or where you're stuck, you can always approach the team leader.
	(Nurse) Like, let's say if somebody has a social problem, yeah, you find that many times they, they have no one to turn to. And so, it affects their performance. Like, for example, coming late and nobody bothers to ask you why you are late. Why do you how come you are an habitual late comer? You know, there must be something. Yeah. But you see, nobody asks or nobody cares and therefore, this colleague feels like, "I'm not cared for," and therefore it affects their performance
	**LD26: When leaders are supported in undertaking their role (C) they will have greater capacity (M) and be more effective (O).**
	(Senior Nurse) Whereby there, I need more. I need more support staffs who can also work on the equipment. So that one is about shortage. Supplies. We need more machines. The machines we have, they are not, if I talk of machines, general, the equipment's they are short. So we need more supplies.
	(Nutritionist) It depends, with what the, sometimes their hands are tied because they are, the, the problem lies within another unit or the hospital, like finance or supplies. So if there's some things are missing and they're making your work, not move well. Then I always tell them, and now, when they get to their end, now they'll tell you now the far we've tried now the ball is in this court, so, it means there are things that are beyond them.
	(Nurse) And out of those maybe you have around 5, 8 about the… maybe you have oxygen they are about 5 you have machine, CPAP they are about 3. ... you see now this one you feel like now you just call the management to come and see what I'm supposed to do and what I'm not supposed to do. .... But you see with that you also have that gap that what if they come and they are like… you see now you get……. Fear, fear, fear there is that fear that what will happen.....
**Inclusive, collaborative**	**LD27: When a leader is inclusive and collaborative in their approach (C) diverse staff will feel encouraged to engage in unit discourse (M) leading to improved awareness, staff satisfaction/ sense of belonging, and investment in team rather than over-reliance on individuals (O).**
	(Senior Nurse) The knowledge in newborn unity is quite a lot. ……Sometimes we can get a condition, and you find that the, this patient is not being managed, the nursing part is not being managed well. So in such a situation, I even organized for CME at the desk and I talk about that condition on the management, on the nursing part of it. So we let each one of us at the nurses' desk to share and put the..., come up with the inputs on how better we can manage and especially like now when we have this issue of X, you cannot manage alone, it's teamwork. You have to involve other staffs, but us nurses, what are we not doing?
	(Nurse)... with the new leadership, I have seen as if people have changed and have become positive... That is my opinion, but I don't know. But I could see people that there is no pressure. I think it's because of communication. Maybe people sometimes when you communicate with people, and give them information about what is happening. Yeah. And then you do CMEs every time some people pick some information and utilize so like. I think communication, I don't know, but I feel people are better now than sometimes and because like it is like maybe the in charge is listening to them, you know, when you listen to them, even if you don't give somebody something, sometimes they feel motivated.
	(Nurse) …. so the doctor can come and ask you, nurse so and so, what is the problem? Why are the mothers coming back with the infection or which medication can we use? Even if they do the investigations in the lab, maybe the swabs, but they need to get some experience we've been having these cases now and then, so you can advise them on the medication to use, how to dress the wound, the antiseptic to use on the wound and also how to handle the whole issue about the mother. Yeah.
	**LD28: When leaders encourage staff (C) staff confidence and competence improve (O) due to sense of teamwork, belonging and able to be themselves (M).**
	(Nurse) ....she's one person I would say mentored us so much. Taught us to be fearless. To share to, to not feel like your pressed down or something. Cause even in my, in my first times the time I was in admission room, she used to come and support us. And she was such an encourager in that she would just tell you, and, by the way, in fact, I remember one day I was so overwhelmed, she came and started assisting me and I hadn't asked her…..

	(Resident) We are being supervised by our seniors. Most of the time the fellows, the consultant, yes. Let's say a more of a mentorship kind of a program, yes, and that's observation with, yes. So that is the main thing, yes. And most of the time you the, the fellows and the consultants, so they will mentor us, they will teachers, they guide us, yes, kind of a family.
	(Nurse) Honestly speaking, my, the current in-charges also motivates me in a way. They really support me, they supported me so many times. So, I feel that they, they also motivate me in a way to work in this department because I've been here for quite some time now and I feel that them plus other colleagues also motivate me in a way to be here. And that aspect of me being able to make a decision. You see some departments you cannot do a lot without the influence of a consultant or an MO, but here you can do so much before even a consultant comes to see that patient. So, that motivates me, that freedom to manage patients. The ability to deal with that case and manage such that when a consultant is coming, he's just repeating what you have already said or done.
	**LD29: When leaders have established a regular forum for open staff discussion (C) it can be used to correct practice and educate (M) leading to whole team learning (O).**
	(Nurse) It, it gives, it gives people a more sense of responsibility. Responsibility in the sense that you find some things other people overlook, but at the end of the day, you feel like, no, it should not, it should not have happened the way it did. So people raise it, they discuss, and then at the end of the day, it's basically just trying to enlighten us that you should be more careful in maybe carrying out a certain procedure or handling babies in a specific way. Yeah. It goes a long way.
	(Senior Nurse) Eh, when they come up with those recommendations during our meeting at the desk, I now just go through to such a thing from whatever we've identified. I talk about it, generally, I don't pinpoint …… But I talk about the unit. Then sometimes I even welcome them to give their feedback on the audit that they did, and the recommendations. Then from there we pick up and we do a follow up. Now I do a physical follow up. .
	(Nurse) Now…now that one is something that is communicated in meetings and people are told like irregardless of how busy you are this is what is supposed to be done. So maybe she'll also tell you personally......that there is a gap here, there is an omission that you need to correct.
	**LD30: When leaders integrate invited participation and open floor opportunities into meetings (C) belonging will be enhanced (M) and teamworking improved (O).**
	(Nurse).... with the new leadership, I have seen as if people have changed and have become positive...That is my opinion, but I don't know. But I could see people that there is no pressure. I think it's because of communication. Maybe people sometimes when you communicate with people, and give them information about what is happening. Yeah. And then you do CMEs every time some people pick some information and utilize so like. I think communication, I don't know, but I feel people are better now than sometimes and because like it is like maybe the in charge is listening to them, you know, when you listen to them, even if you don't give somebody something, sometimes they feel motivated.
	(Senior Nurse) Yes. Because to me, I allow anybody and everybody to express freedom of expression. And that is why at the desk during the meeting, we create time for AOB, who has something they feel they could share or talk about or any experience that you have, you let the people open up on the issues they have or in case they have. So to me, I feel they have freedom of airing out what they feel and when they air it out, I take it positively.
	(Nurse) By encouraging, encouraging one another, encouraging one another to bring, to air out their views, to feel free to communicate, and whenever there is a problem one should, should be, should be free, you know, to say it and see if we can we can, we can, we can come in and, let's behave like a family the way a real family normally behave. I think with those ones, some of those who normally confined or mute themselves, can be able to open up.... Encouraging communication, that's it.... Well, whenever you ask them questions or whenever you want to, you want to get something out of them, just do it by writing. Because whatever, whatever their, whatever they were to talk it, if when, whether they were to, whether they were to air it verbally, of course, it's inside them. So, when you ask them by writing something, obvious, they'll have to respond, you inbox them.
	**LD31: When the unit has an inclusive WhatsApp group (C) contributions are theoretically open to all (M) which may contribute to discussions and consensus building (O).**
	(Nurse) Mm. Sometimes occasionally you may find some webinars, maybe somebody shares in the group. There is this and this webinar on this and this training.... Yeah, you can send maybe the document or the link. If somebody's interested, they can check.
	(Nurse) Yeah and most of the times even we have it on the… we have maybe like on the WhatsApp, we have a thing that we keep on updating ourselves what is happening and even the conditions, like for example, we have a new condition in the… in the unit we'll keep on being updated. So the flow of the information is good.
	(Nurse) Like, information like us here, we have our WhatsApp group whereby if I have educative material I just send it on the wall and ask people to go through it. Also we normally have CMEs. The CMEs we have them where we are in different target groups.
	(Nutritionist) the whatsapp, the, the meetings, aah, I think it brings people on board and actually have that kind of discussions, yeah.... which makes people free,... free to talk, they feel motivated at long last,... to give their best.
	(Nurse) Mostly, mostly you'll find that maybe there's a condition which has come up, a mother has come with it, so mostly that is when we will look at it, we all just look at it, we discuss it or there is an information. A condition has come up and if you're not in the shift then someone can post it and we start discussing it on the[WhatsApp] group, about that condition
	**LD32: When an online platform is used for meetings, (C) diverse staff can theoretically join, (M) creating opportunity for collaborative working/ learning (O).**
	(Nurse) No, we have only one [WhatsApp group] in the unit for nurses, but I don't know if the doctors have their own..... The common platform it's during this Zoom meeting, that's where we [nurses and doctors] meet.

	(Resident) So mostly CMEs, you can join from, okay, you can join even from outside. Like, maybe a ward is presenting on a case and you join, it doesn't have to be just NBU.... Yeah, there are online, all these CMEs are online. So, you can join and then you get more perspective from a case.
	(Resident) They were online.
	**LD33: When staff and students are included in meetings (C) they will feel a sense of belonging (O) due to their share in collective awareness (M).**
	(Resident) Face to face during ward rounds. Then we also had the continuous medical …, the seminars and the meetings we used to have…... They were online... I don't know which day is which day, but we usually have one on Monday and one on Wednesday, and then we also have a mortality audit once a month.... So one day is for the whole paediatrics, the other day is for the neonatal, I can't remember which day, one day is for the neonatal.
	(Nurse) …and also other carders but CMEs mostly we are nursing…we are nurses there. Yeah.
	(Nurse) Yes, I have that freedom. Because if something goes the wrong way, actually I don't hesitate, I measure it. And I don't… Especially in the meetings, you find something is going wrong and it involves everybody. In the meetings, you come and I have an issue and you correct it and they take it positively. ... I can say not everybody, but most of us, most of us, we have that freedom to share what is happening....
	**LD34: When educational meetings are tagged onto handover meetings (C) staff are already assembled (M) so will enhance inclusive/ equitable teaching and learning (O).**
	(Nurse) At the nurses' desk station. Every morning. Those three days. Morning hours.... Yes, at the front site. We usually meet there early in the morning...Even the CME, that's where if you have a CME, you give it there. On those specific meetings. ... We have meetings and within that day of meeting, we also give CMEs. .. We discuss about the management of patients, the newborn unit, if there is any introduction like, caffeine management, administration and care of a patient on caffeine administration. And how to manage those patients.
	(Nurse) Yeah, they are. But I've said you utilize those meetings in the morning like we yeah, the CMEs here during such moments, we asked for any issues that members could be having. So we utilize such moments to get feedbacks and maybe we don't have enough time. We plan for a CME for that particular thing that has been raised.
	**LD35: If ward rounds are not inclusive, e.g. favouritism, fear (C) a clique evolves and some staff are excluded from problem solving work (M) with reduced ability for learning and collaborative decision making (O).**
	(Student Nurse) I feel ward rounds now there have been left for doctors in the hospitals. And I feel they have left one part of two people who are very important. They have left out the nutrition, they left out anything about nutrition and we have left the out nurses, this nurse has stayed with this patient for 24 hours. You have to come there for 15 minutes. I think it is good to ask me how your patient was before even going to hand it over to a registrar who came in the morning, did the ABGA very fast and sat down to write the summary. They have not even checked on the patient, but that nurse has a lot of information about the patient, but she will keep quiet because she is not asked.
	(Student Nurse) Mostly the doctors communicate during ward rounds. And then they are giving orders for the care of the patients that are supposed to be given.
	(Consultant) I think it is culture. I sometimes feel we don't engage them enough in the ward-round, to feel useful. For instance, in my ideal world, I would like to see a baby, listen to the presentation (from the interns) and listen from the nurse…like actually hear from the nurse: What is your feel managing this baby? Many times, the nurse is the silent bystander… and within 15 mins they (nurses) disappear on to do other things where they feel more visible.
**Situational awareness**	**LD36: When a leader is proactive in gaining situational awareness, e.g. attentive listening during handover, present on unit, intentional, interest (C) she will better understand the needs of the staff, patients and the unit/ have oversight (M) and will be able to identify and mitigate gaps (O).**
	(Consultant) Yes. Newborn care is very dynamic, so dynamic that 6 hours makes a lot of things, so that is why when I come in in the morning, I tend to just first know what transpired. And the best person to give me a summary of that is usually the in-charge because she has a sort of real-time access on the nurses on the shift. Sometimes I may want to unpack that with the specific nurses, but she usually she has an overview of the goings on in the unit.......
	(Senior Nurse) Eh, sometimes in the morning, in the morning I enter inside the nurseries, during report handing over so that I'm able to listen on how the report is being handed over. And to know the patients to understand the patients that I have and in case there are any issues, so that we are able, administratively we are able to handle. So I get that information and still where we get that they are shortcomings, we again, discuss it, during our meetings.
	(Nurse) First of all, we have the in charge, when she comes in the morning, before she received the report at the nurse's station, she… she goes round the unit and this also should happen if like if I'm the one who is in the office,... The one standing in for the in charge, so the in charge goes round the rooms and checks on literally checking on spot checking on let's say where we write our reports, cardexes whether they are properly written.... Yes, today she went and did that... Then whether the…the observations are properly… the observation charts, monitoring charts they are properly put with all the data, biodata of the patients and all that. So in other words, the in charge does a sport check on all the rooms.
	**LD37: When a leader has gained a good understanding of the needs of her team, e.g. skills mix, availability of consumables, professional requirements (C) she will be able to provide effective and timely support, e.g. supplies, allocations, supervision, tailored reminders (M) making the working shift much easier for staff and more enjoyable (O).**

	(Nurse) That is one. And in any case that you may find that you are stuck somewhere suppose either administrative issue or maybe you have an issue with a mother here, when they're team leading, you can comfortably approach them, and they're ready to assist. Are ready to assist in the sense that they offer, they're able to come up with a problem solving solutions that are going to, to steer you to, to make a shift smooth. And also to help in terms of even maybe you have lacked something, supplies. They make connections here and there. Maybe call different departments should you lack something. And majorly another thing is probably should get a, a, a procedure that you need two nurses, cause one in particular, I know there is actually the first time I worked in ICU, I was with (first female name above), and I cannot forget that day because, you know, it's a place that you have not been so well acquainted with and especially in NICU, where the neonates are. It was a very easy shift for me. Cause at least she would tell me this is what we do, 1, 2, 3. Even if it was just a half shift, but at least I was able to be oriented within the, those six hours. So you find it at least it was, you'd able to maneuver.
	(Nurse) Well, because there are those who are ready to help, let's say like they are the team leaders, you'll not, you'll not be stuck. You'll approach them freely. Let's say you're in a busy room, you're giving medication, you have IV fluids, you have transfusions, they will be there to help you. They'll even come asking how they're doing in your room. So when you are in shift with them, you feel you will not, you will not get burn out because they will be there to help you.
	(Nutritionist) I think good teamwork and as a leader, the leader of the unit should be very wise on how to handle her staff.... Wise means that you should learn your staff, all of them. You know that this one is like this, like that. And then even as you plan duties, you try to balance those equations. So that, you know, even with this duty, you have a strong point person, in case of anything.
	**LD38: When a leader has active situational awareness (C) she will more readily identify lapses on the unit (M) enabling her to offer 1:1 correction and support to staff (O).**
	(Senior Nurse) I think I've done quite... if I identify, sometimes mothers can come and talk. Sometimes you just observe yourself as you go around. So, such staffs, I always call them in the office. I sit them down, ask them in case they, how is, how are they fairing on? Do they have issues in the unit? Do they have issues about themselves first? So after establishing that automatically, some will say yes, some will say no. Then now I asked and deliberate towards, I narrow towards the problem. .....So I talk to them. Then after I've established that actually they have a problem, we talk and see how to help one another.
	(Nurse) Supervision, so she gets first-hand information, she supervises by correcting. [interruption] by supervision, when she listen to the report she can tell where there are gaps and she is able to ask why they are happening and to teach and…and… let people give direction on some issues. Then officially yeah mostly it's going and yeah.
	(Nurse) In terms of supervision, the supervision is usually done by the in charges but not always.... Most of the times they come in the room and see how maybe you have worked, there are things that were supposed to be done and they are not done, maybe they'll have to ask you... if maybe you're in a room where vital observations were not done, they'll…they'll have to be concerned about it.
	**LD39: When staff recognise the value of an overseeing leader (C) they will proactively share relevant information with the leader (O) as they expect support for themselves or their colleagues as a result of the oversight (M).**
	(Nurse) thankfully, you always have team leaders in every shift. I'd approach the one who's currently present just to, to hear their views. And then I'll be like explain how this is done, even if I know, and there is that person who mentioned how it's supposed to be done, so that I can have clear information on how I should be going about the filing of that document.
	(Nurse) And also now our in charge is in the office 24/7 for the day. She, she rarely leaves. And that's where also if you want to make a decision in your room, that's where you go. And also her she's able to communicate with us, one-on-one in the rooms. So I feel communication has really improved even in the care of the patients.
	(Nurse_) So the team leader's responsibility is that everything is running smoothly in the unit. If there is anything that needs to be sorted, any communication issues. In terms of resources, we have enough resources in our particular nurseries. Ensuring that she answers calls, ensures that the issues of the family centred care issues, if the mother has any concerns, that is her responsibility. Something that cannot be handled within the particular nurseries. So that is her responsibility to ensure that she oversees that everything is running smoothly.
	**LD40: When a leader is proactive in information sharing with other cadres (C) this will improve situational awareness and continuity/ consistency across the whole team (O) because shared understanding has been gained (M).**
	(Senior Nurse) ......So the spirit of teamwork, it's very good. And I think that one is motivating staffs. The way we are also working with the doctors. The doctors we have, they are good doctors, you approach them and give suggestion, you just talk and agree, there is not this one is learned, this one is not, we are all working as a team. What else can I say a motivator? I think I can say basically teamwork.
	(Nurse) Even on patient management you can be told like this baby should be… like if we have an infection, these babies are supposed to move to this room then another one says no this one, so at that point exactly where do we stand, so you get that from the consultant and the in charge so that we are at par and from there now you can also tell people it is this way and everyone it can be put on the wall, this is what we are doing.
	(Resident) Yes, or even the doctors that you're within the same cube, they can tell you or the seniors will tell you, "This and this is going on, this is what we do." Otherwise the, the other information is normally receiving from the seniors and also the team leader, then the team leader also will share with the, with the larger group.
	**LD41: When staff are confident that their leader has taken responsibility for effective oversight of the unit (C) they will feel able to focus on and be effective in their allocated role (O) because they will know that the leader has allocated remaining roles and tasks to others, so that the overall goal of patient care delivery will be achieved by the team (M).**

	(Nurse) Supervision. It's good. It's good. Thankfully you find even when okay, let's start with the team leaders, because those are the main, the main icons in the supervision. At least they go around the rooms just to find out if everything is okay, make sure supplies are there, contact the necessary offices. Should someone need help outside, outside...i would say it is cool, it's say, it's up to, they're up to task.
	(Nurse) Support… Especially, you are in a room and your supervisor is there. Maybe you needed to order a drug and you are to order some medicine or drugs for this patient. They have been ordered by the doctor and you don't have that time. Your supervisor is there to assist you and once you approach them, they do it and they order online and it comes to the patient. Also… You can miss something like tubes, like feeding tubes in the room. They are somewhere in the store and you don't know where they are. So, your supervisor should provide that. Once you consult them, they assist.
	(Student Nurse) So, I noticed for the nurses when they have any information, they give to, they give it to the team leader. We have respective team leaders of various shift. So, they would give information to the team leader and then the team leader will relay that information to the in charge, so that the following morning when, when, when, when we come, it is now the in charge who would tell us what happened; like a brief, a brief information of what transpired and the way forward.
**Appropriate delegation**	**LD42: When staff are allocated to patients in teams (C) skills mix can be tailored to need (e.g. neonatal nurse) (M) thereby improving supervision, learning and patient care (O).**
	(Nurse) …one sio [not] character wise, if I find I'm in a shift where I'm the only senior nurse that one I'm not happy because I know in case of anything I am the only one. So I would want someone… a shift where we are… we have… it is more what? No…not having one carder we are mixed.
	(Nurse) Fresh graduates, I can say they are also, they're real eager to catch up, with whatever is happening. So when somebody comes, they're mostly attached to a person who has been here for some time, to show them the way. ….You have to be attached to somebody who will maybe show you how things are done, around........ Then you cons, if you bring a neonatal nurse you, it's seniority, I think you cannot tell a person who has not specialized, to orientate or precept somebody who is specialized. Because the job description is different. So they do consider the, the cadre and maybe the, the shifts that the other person is working in.
	(Nurse) You know like here, they have...when they are students, there is a clinical instructor in this unit who is allocated to students, but that doesn't mean that it is the work of the clinical instructor only, because the clinical instructor will allocate those students, and will take them around the unit to show them about the unit. What happens in the unit? But after that allocate them in rooms. Now when we are in the rooms, that is when we mentor them, we tell them if it is this, it is done like this. If it is observation it is done like. It is making fluids you show them.
	**LD43: When a leader/manager is clear about their role (C) they will be less overwhelmed (O) as they will be able to differentiate which tasks should be undertaken by them, and which should be delegated/ allocated to others whilst providing support (M).**
	(Nurse) Okay as a, when you are a team leader one thing you when support you have to give to the staff if you don't then you are failing, ensure the consumables are within reach. There is really… it is really very bad when a…a nurse who is in NICU with ventilated very sick babies having to come for things in the store. .... I should be able to make them be near them.
	(Nurse) Supervision, it does. Because when somebody is put at a team leader, you're the supervisor for that to work. You're supposed to be going around the unit, what is lacking, what is needed, as a team leader. In terms of mentorship, any new staff coming to the unit is normally allocated somebody. We are giving that person a certain period to work together with that person. And that person is competent to work alone..... Students, when they come in, we have been having a student mentor who has been with the them, orients the students, allocates them.
	(Nurse) My juniors like today we need to intubate the baby in one of the rooms. So, if the nurse in the room needs some requirements like the baby needs a mechanical ventilator, she has to liaise with me to see that we have received the tubing's from x so the baby can be intubated, I liaise with the nurse in the room to make sure she continues to resuscitate the baby and I talk to the nurse who is preparing the equipment I send the messenger to get the tubing's from x, so I have to co-ordinate to make sure that everything is in place so they can set up the mechanical ventilator then we connect the baby.
	(Nurse) In terms of supervision, the supervision is usually done by the in charges but not always....Most of the times they come in the room and see how maybe you have worked, there are things that were supposed to be done and they are not done, maybe they'll have to ask you..... if maybe you're in a room where vital observations were not done, they'll…they'll have to be concerned about it.
	**LD44: When leaders can differentiate nurses based on qualifications and/or seniority (C) skills mix can be tailored for each shift, improving staff satisfaction (O) as it is easier for leaders to identify the range of skills within the team (M).**
	(Nurse) AHHHH...Yes, you can come to a shift, and then you ask ...these people did not see when they were making the roster? Because you are the only senior nursing officer or the rest are young, they are young, they are junior. So, you can see when you know you need some advice, maybe regarding... they have, maybe they have called, they want to admit a baby, you know there's something you need to communicate with somebody who is at your level or higher. .... Not really but sometimes you can find that in the unit, you are the only senior one. So, they need to balance to get people to balance. That's what I mean
	(Nurse) When you have new people who are fresh from, when you find it's only one or two people most of the time, one or two people. And I feel like we are able to take them through. First of all, when it comes to now, how we've been divided into groups, I feel they won't miss somebody who's they can approach and ask. And also when they come, they're attached to someone, who is able to take them through how we work here. So when they're on shift, they work with them in the same room. Yes. I feel with that it brings out ease, and confidence.

	(Nurse) I can say it's good because like when we have a new staff, they're usually put under someone for like a month, so you'll find you'll be doing the same duties. So if you're in this room, you will be there with that someone, you show them how a certain procedure is done. Yeah..... Like a month..... Yeah, the students when they come, they are all, they have their supervisor and then when you are allocated with them in a room, you are, you are supposed to show them. So, you delegate, for example, you can tell them, you show them how a certain procedure is done and then you let them do.
	**LD45: When leaders are aware of skills within the team and delegate appropriately (C) staff satisfaction will improve and leaders will have greater capacity for management/ support (O) because of empowerment of others to undertake certain leadership functions (M).**
	(Nurse) There is teamwork. And then there's also equality. I feel like we are being treated, or the work workload is being, being directed equally according to now the persons like the person, the qualifications. Like now you can't be put in, in ICU and you know nothing about ICU, which I feel is okay, to make everyone feel comfortable with that. There's teamwork, there is the equality.
	(Senior Nurse) Supervision, eh, it's a responsibility of all of us, and far more the team leader or the ward manager. And you see, you can't be able to supervise alone or to manage alone. So I have identified mostly senior nurses who have experience in the unit. And have delegated. Delegated, because since I can't be able to do, yes, I have delegated some of them . I tell you, you are going to be in charge of consumables. Ensure when they come from the store, you check the (ordering form), confirm. Ensure they have put well, and they will be entered in their consumable book. And ensure, be checking sensitize people, when somebody picks things from the store, has to document somewhere. Then I, I delegate somebody. Your work, you will be going able to check whether vital observations are being done. So it's like targets have given people.
	**LD46: When a leader does not delegate appropriately to others in the team (C) staff satisfaction will reduce (O) because their skills are not being utilised/ they are not being trusted to engage in leadership (M).**
	(Nurse) The other thing, recognition.... Yeah. Sometimes you're not recognized. You've gone for training, you've gone for those higher diploma but, maybe you've gone to degree but it is not recognized.... You go to the same place.... Where you came from, yeah, so there's no motivation.......Maybe you can be placed in a, in an area whether you have …. Yeah, that matches what you've learnt.... Mm-hmm. Even if you don't have an ICU unit you can, you can find a place where someone can feel a bit recognized. Yeah.
	(Nurse) coz now if you're the in-charge, and you ask a very junior nurse to work for you,... to take up your role. You don't come to work that day.... you ask someone very junior to work for you, who is still on probation. I mean, what is that?... you see now.... so, that's what I mean.... but to me, it's looks very unusual.... but to me I find it very unusual, to do that.
	**LD47: When a leader does not adequately delegate to others in the team (C) the leader will be unable to perform their role effectively, leading to staff frustration (O) due to overwhelm and subsequent perceived inaction of leader (M).**
	(Nurse) Yeah, it can be improved, let's say Nurse in charge is the only one there and the X, but I think if we can subdivide, let's say there's a, there's a, instead, instead of waiting for (name, in charge) to come in the morning or during the day, we can have some small groups, someone sort of maybe let's say team leader or certain, yeah, while when you're working you can, you can easily air out one, two, three out there so that, not that you are the one on duty and also the same person going to other departments looking for whatever that is not there.
	(Nurse) It's not bad. Okay, depending on which, which if it's my immediate supervisor, she's normally patient so she's advising or supervising us there, but sometimes when overwhelmed (laughs), you can really be a dictator, she can be, she can be harsh. Of course, you have to understand because there are some matters which have to be, to be attended to and there's expectations on the course and I take them positively, I take them positively.
	(Nurse) So maybe, you know, there's, there's a specific person dealing with specific things. And they're not around. They don't do not want to disturb them when they're away. So such time is you may miss the information..... there could be somebody, but they also don't have the, the, the whole, the whole information. There's something missing.....

LEARNING

Structured learning on unit	**LN1: When information/ clinical guidance is communicated through a formal meeting/ CME (C) it promotes common understanding of best practice within the team (O) because the information can be disseminated and legitimised (M).**
	(Nurse) Well, in this situation, they are always offered opportunities during seminars to go to gain more knowledge. And during the continuous medical education, they are brought to the level where they should be able to understand what they didn't get clearly. Yeah, so continuous medical…
	(Senior Nurse) Then the CMEs that are held every Wednesday. Yeah, the new information is delivered there.
	(Nurse) Now…now that one is something that is communicated in meetings and people are told like irregardless of how busy you are this is what is supposed to be done. So maybe she'll also tell you personally... that there is a gap here, there is an omission that you need to correct.
	**LN2: Where mentoring immediate juniors is an entrenched norm (e.g. medical team) (C) continued supportive relational structures (M) will lead to trust and enhanced learning (O).**
	(Resident) We are being supervised by our seniors. Most of the time the fellows, the consultant, yes. Let's say a more of a mentorship kind of a program, yes, and that's observation with, yes. So that is the main thing, yes. And most of the time you the, the fellows and the consultants, so they will mentor us, they will teachers, they guide us, yes, kind of a family.
	(Medical Officer) So, for the MO interns we are supposed to mentor them anyway. .... So we kind of if they don't… we tell… like we do CMEs as well...... before they present they send us the presentation… correct them stuff like that.. then even among ourselves. Like maybe… maybe not just me we correct each other
	(Resident) When you're new, you are oriented for two weeks where you shadow the senior residents....You shadow, you shadow the senior residents for two weeks. So, you're not allocated any room alone, and you shadow. So I think that really helps in orientation. .... Every morning, there will be a ward round for the newborn ICU, for the NICU. So, there'll be a ward round which I think is good because you can get a lot of feedback on how you manage you manage you patients. Then twice a week, there's ward round for the other rooms. So, I think the supervision is pretty good. Then there are always fellows in the unit, so they also supervise you.
	**LN3: When unit staff must assess students (C) teaching relationship will be influenced (O) as staff will require awareness of student competency, and students will recognise power/authority of staff (M).**
	(Nutritionist) Yes, because at the end of the week, you're also supposed to do an assessment, to fill for them an assessment form regarding their performance. So, you should supervise everything that they're doing, if they're doing something wrong, you're supposed to be there to correct them.
	(Nurse) No, they are assessed now from the units. Say students are in this department, the in-charge now assesses them and gives the feedback to the student mentor.
	(Resident) There is a clear understanding that we're here for just a time and then we are being evaluated at the end of the rotation. So you also are very keen not to ruffle feathers, so you want to do your best and leave. Because every place has its own culture, there is a way people have been working before you came, there is a place they'll continue to work. So for the three months that we are there, you don't want to, yeah, you don't want to ruffle feathers too much. So, I would say no......
	(Resident) The consultants because they're the ones to sign me out and to give me marks.
	**LN4: When there is no clear go-to person for students on the unit (C) they will be unable to share questions and concerns with staff (M) leading to stress and misunderstanding (O).**
	(health worker) ... Cause like the people that we would have approached, you know, with this behaviour, are the ones who are present when these behaviours are happening. When these words are being said, they're present in there, and they are like, and they just laugh it out. So, you are like, okay. I guess that's the new normal, and you just move on.
	(Student Nurse) ...nobody shares that with you, like, you're not told on the first day that if you have this and this, this is the channel, you can talk to this person, this person. I've never heard that.... so, you get so stressed, you don't know who to talk to... you don't know whether to continue with doing things as they, okay, you know they normally say, there is this quote ' kwa ground vitu ni different' (reality on the ground is different). .. what you learn verses what is being practiced are totally different things....
	**LN5: If students and new staff receive orientation by unit staff (C) expectations and support are clearer from the outset (M) leading to improved experience (O).**
	(Nurse) When they come they are told how to be responsible staffs and also knowledgeable. They should work as per their objectives and they should be, what's the word? They should be somebody should be ever there at work place should not be somebody disappearing.
	(Nurse) ..mostly the students are, generally, orientation to the unit... reminding them on their objectivity, their roles... their scope, and supervision.... I always encourage them, unless you're sure, don't do anything that you ain't sure. At least you seek advice, or you can do under supervision... you can give it a shot under supervision if you're not sure.
	(Nurse) .. first of all, we should have a…., when maybe, maybe when we arrive, there is the whole orientation and so, we get this what, we get these papers from school, like what you should do in the ward,... like your goals in the ward. What you should do before the end of the rotation. I think people should go, step-by-step.... looking at those different, those different goals set by the school, to reach your, to reach your goal at the end of the rotation.... they don't even ask for the paper these days....

	**LN6: If training institutions do not follow up students (C) students who have poor commitment (M) may have very poor learning (O).**
	(Senior Nurse) But for those institution, like the (name of school) staff, students who come with the ......clinical instructor, they know that here we must learn. So if students are followed from school, they will learn. If they are not followed from school attitude, attitude, that I'm just passing by. And especially for those ones who don't have exams. They know, I'm just passing by, if it's work, I'll do it once I qualify, here I'll just pass by. So you get that these students, sometimes they don't learn.
	Observations
	**LN7: When there is poor collaboration between training facility and clinical unit (C) lack of clarity, oversight, delegations, and responsibilities (M) may lead to a chaotic learning environment, discord, and frustration of clinical staff (O).**
	(Nurse) ...first of all, we should have a…., when maybe, maybe when we arrive, there is the whole orientation and so, we get this what, we get these papers from school, like what you should do in the ward.... like your goals in the ward. What you should do before the end of the rotation. I think people should go, step-by-step.... looking at those different, those different goals set by the school, to reach your, to reach your goal at the end of the rotation.... they don't even ask for the paper these days.
	(Nurse) What happens, I think there is a slight gap when it comes to updates that you receive in the hospitals and what is taught in the schools, because you'll find students, some students still have what was taught about three, five years ago as procedures. So, when they come here, they find it totally different with what they learnt in school. So, if I will say there is that gap between the training institutions on updating themselves on the current trends and events. Yeah........... you find even a tutor will come and as, as you go around with them, taking them through your procedures, they can even find that chart, maybe on whichever medical obstetric emergencies, they can find a chart that you have, and they look at it, and they be like, "When was this updated?" So, but at least we appreciate the attitude that when the find this thing is new, they will always document it, somebody will take a screenshot. They will want to go and update on their notes as they teach. Yeah.
	(Nurse) Some they know some they usually say that they are learning in the ward so I'm not very sure whether they do learn in the school, because you see even some of the procedures, even some of the things that they meet in the in the room it's like it's very new to them.
	**LN8: the way in which CME is structured (C) sets the tone for learning on the unit (O) due to demonstration of values around sharing of knowledge and inclusion (M).**
	(Nurse) but we also have CME, also have CME's Mondays and Fridays where if you have something to share with the rest or a piece of new knowledge, then you can have the advantage of the congregation and share whatever you want to share
	(Nurse) ... having I think, having time, maybe the CMEs can be held per unit for everybody to get the information.... yeah, everybody can be there... it should be per unit, maybe, let's say today it's the general ward, tomorrow NBU...so that…., you know every unit has different issues...so, I think that will be for the best.
	(Student Nurse) Seminars we quite often get being done within the unit because of a number of conditions, especially the several outbreaks of infections, it makes us have a lot of teaching and researches being done and therefore, we have been having, almost every month, we get seminars being done within the hospital. Yes.
	(Student Nurse) There are some that are organized by (hospital). Then there was the one by the Pediatric Protocol Update, then we have the one by the WHO.
	(Student Nurse) There was, there was a seminar on CPAP though that day I was not in but there was.... students attended. Yes, it was done in the unit even
	(Nurse) Ok, we have if somebody wants to share something, we have what we call continuous medical education, the CME. So, if somebody wants to share something...Because we normally, let's say we have our report..., which is the general report about the ward where everybody converges. That is Monday, Wednesday and Friday. So, if somebody wants to share knowledge or update people on something will take advantage of those three days. Yeah,
	**LN9: Convening of staff on a WR (C) can facilitate collaborative working (M) improving awareness, learning and a shared patient care plan (O).**
	(Nurse) They're also nice, in terms of sharing information about the patient, although I, sometimes it'll not be written in details, but there is some sense of knowledge sharing. Cause in case you do not understand anything you will consult the doctor who was concerned, what is this and what, what does it entail? And they will gladly share.
	(Nurse) So they'll be like asking what, what has been done to the baby since maybe the last round. If something has been done, if it's not done, why it's not been done. And sometimes they'll now they'll explain to you, further, why something needs to be done. Like why you need to do sugars this oftenly, why you need this done and why you need it done promptly not to be delayed.
	(Nutritionist) The experience in the ward round is good. It's very educative, in fact, for me, after I attend a ward round, I can document a file without making a lot of reference. Because I know that in that ward round the doctor gave a summary of that patient. It also, assuming I came from leave, I've not been around for like two weeks or more, it can bring me up to speed for what is being done for those children, without having to, to peruse through the file. So me I appreciate ward rounds and I feel bad that now I cannot be in all of them at the same time. ... Mostly I listen, but if there's an input I occasionally step in, or if I'm asked something.
	(Nurse) Yes, yes. We usually get because most of the time you get the updates of the current management..... Yes, mostly from the doctors or you know, it's a lot of so many people that going for ward round...Yes. So if there is an update of something, somebody with update will just mention it and sana sana [mostly] even the doctors they usually have everything on the fingertips
	(Nurse)... it does. It does because it's attended by different cadres. So, while you give information then there is a consultant there maybe to prove, to prove the information, then clarify on the same.

	(Clinical Officer Intern) Learning there is any time during the ward rounds... yeah. Even after the ward rounds maybe maybe lets say there's condition maybe that is like a bit challenging to everyone... We share thoughts together... Yeah then you come up with one conclusion.
	**LN10: When a consultant/ senior doctor and staff are physically present together on a WR (C) there is an expectation that learning and teaching will occur (M) encouraging staff/ students (O).**
	(Student Nurse) Like her ward rounds were so thorough. A lot of information about the, the baby, the file, the investigations. As in she would not leave anything unturned..... A lot, even during the ward round times, even if I'm not in that, like if she is in ……., I used to go and hear what she saying.
	(Student Nurse) The experience with the ward round was kind of, well, because they could discuss the diseases at a much, at a much, broader view, on how the condition is being managed. And they could give an history on how the previous management have been and the impacts of the management to the new born..... You were free to ask a question.... And they could answer you.
	(Resident) Ward rounds are mostly a learning experiences, so you would give, you've taken the history, you give your treatment plan, you've done your investigations. So now, the fellow, the consultant when they come in, they're able to add on. Yeah, and you're able to now do better next time when you meet the same patient. Now you won't forget what you forgot in the first patient. You'll be like, "Now I'll do all of them," you know.
	(Student Nurse) So, information when we learn a new thing you know during ward rounds, when we have the consultants coming in, the professor coming in, you know there is something new that they will always say, something that you had not heard before or something that you are doing wrong then you correct. So there is always discussion about a particular patient on the management and yeah. So when we are having a discussion as students, you can chip in and say, today the doctor came, this and this was discussed.
	(Clinical Officer Intern) During the ward rounds …each…each condition that we are let's say presenting to the consultant, to the MOs… The aim was the consultant most likely consultant they go deep concerning that condition. .... So you get at least from that knowledge that we had…. …you are added some more. ..... Yeah because lets say if maybe me I was interested to doing this this way but consultant asks by the way currently the guidelines have changed they are being done this way. .... You see that even you, you will adjust from the norm that you had..... Yeah because it's everywhere from the students, the nurses, the nurses... during the ward round there are the interns… there are the students and the nurses.... Qualified yeah and the MO.... So when the consultant is like telling us concerning or learning teaching us more concerning anything that maybe right now is a bit shallow to us… she expound about it all of us we gain.
	**LN11: When new learning is effectively disseminated on the unit (C) all staff/students will benefit (M) and patient care will improve (O).**
	(Nurse) Yeah. That's why we have meetings, so, during the meetings is when they teach us.... Or maybe they post in the WhatsApp group. Maybe if there is a pdf, we go through a, we go through it. Yeah.
	(Nurse) mmmmh, (laughs), I think am one funny person, because aaah, we have a group, so, there is this time we went for, … for nini (that), for training. That NEST training... and I found the information was very good.... and it was really needed in our unit, so, I just took all the material and I shared it on the whatsapp group.... so, I won't choose, because I know, you know, even this person who you don't feel like working with.. in one day or a time you'll find yourself together.... unaona (you see)? so, you have to equip him or her with this skill because you don't want every time to be, 'what do we do this? How do we do?', yeah.
	(Nurse) Yeah. There's also management of bleeding, prevention of bleeding in post-delivery, I went for a training, came back and shared that information which has changed like almost, though we went like five of us, we shared the information at different locations, and it has really proved. That is what we practice right now.
	(Nurse) Like I said like those training we go, maybe people did not have an opportunity to go, so not everybody goes. So those few who have gone will share knowledge. Mostly it's like that ...somebody will stand and say I went to such and such a seminar and this is what we were taught. You will share at the desk (nurses' desk), yeah.
	**LN12: When impact of implementation is tangible (e.g. reduced mortality) (C) staff will be encouraged (M) and implementation will continue (O).**
	(Nurse) coz for ETAT, whenever, even when you're alone, in case you're caught up in an emergency, you're good to go.... you just settle everything because you got the ETAT training with you.
	(Nurse) So I got it, I found it very positive, and I tried it and it worked very well. Although there are challenges, we have so many babies, you can't have every baby with one. Sometimes there are those challenges, but if you do the right way, especially the way I was advised, the baby improves within one to two days, the disease is clear. So I found it very positive.
	(Nurse) There is improvement of care in the unit. There is reduced mortality rates.
	**LN13: If training and knowledge received is not disseminated on the unit (C) there will be a lack of awareness and consensus (M) leading to poor implementation (O).**
	(Nurse) Let's say on the side of implementation you can find that you're taken for training and the time that you've been taken for training maybe this nurse has not said that he or she is taking a transfer, she has kept quiet or you'll just be removed from that department and taken to another very different department, so you are not able to pass the information or whatever you learnt to the other staffs. Sometimes trainings it's very unfortunate, you know this is a big hospital, so when training comes its written administration first, so before the training gets to the department that is required, it is like 30%. So the administration person is the one who will go for that training and it's not easy for that person to come and give you that information.
	(Nurse) Because now that I have never gone and I've never been trained on anything, so I don't know. Because you see when you go, you'll learn as an individual and no one will come to tell you that this is what we learnt.

	(Nurse) Like, for example, if, if I'm right, if, for example, if you, you acquired a certain, certain information and you want to share it out to your colleagues, and then maybe you call them as in, "At this time, we're going to have a meeting, so we want to share whatever we've been taught and availability is a problem." Some now people start giving apologies, "I won't be I won't attend." Or even if it's just to discuss it through the WhatsApp, you find that now, people are not even contributing or even asking any questions. And you feel so discouraged
	**LN14: When training is not specifically targeted or mandated (C) it may not be taken up by digressive individuals or people may consider allocation to be unfair (M) thereby wasting opportunity for correction and leading to disengagement (O).**
	(Consultant) That is an area I am trying to figure out… am trying to have conversations on the side, and engaging individuals to see why, because for instance, as we speak, I can mention for you, maybe 2 or 3 people, who I find their skills quite lacking, their work quite unsatisfactory, and yet they don't come for trainings…
	(Senior Nurse) But still you will find those who still they are not interested, you know you cannot force them.
	(Nurse) Don't care attitude that I never went so I don't want to know about it. And number two I won't use it.... The selection may be unfair or maybe even the in charge may be seeing as if I can't, I'm not, I…I can't get anything from that, that education or that information. So it's like those who went let them do....They usually… Really they…they usually like now if you had gone for another training, the next one will come they will select other people, it's not necessarily that it's about the cardre or anything …for them they are fair to me, to me I can say it's fair because even they are those who are going to be selected and say they say no. .... … you'll just give out excuse and maybe you won't go but what we usually do is like there is this…this one like the one we…we just listened about caffeine is like everybody went apart from maybe you had some critical issues
	**LN15: When the hospital is seen to support training (C) staff will have greater opportunities to gain new competencies (M) and will feel appreciative (O).**
	(Senior Nurse).... It [hospital] pays for you and releases you. So they are those courses, even courses, seminars, training. Not all, you have, they are some (hospital name) pays for you, and releases you....Very relevant. I can't be who I am if it wasn't (hospital name). For my master's, (hospital name) released me and paid for me. If it didn't do that, I wouldn't be a master person because the money is too much. Or I might have had money, where do I get two years' release? So to me, I just appreciate (hospital name) for those release because without it.... it's too expensive, but (hospital name) releases and pays for staffs to go for the training, for one week, two weeks. So me, I can say (hospital name) is really doing good on the release.
	**LN16: When posters/ protocols/ guidelines/ SOPs/ job aides are posted on the unit walls in strategic places (e.g. incubator guidelines on wall next to incubator) (C) they provide a trusted, clear, timely reference source and reminder of what should be done (M) and improve ease of work, continuity, and student and staff learning (O).**
	(Nurse) Yeah, they are, there are work instructions in every room, they cover so many things. Maybe for example, let me start with the in brief three, the, the feeding, the feeding schedules that means the baby, those babies with tube feeding, cup feeding, breastfeeding. Okay. There is the kangaroo mother care…. there is schedule on measuring or regulating humidity in the incubators. and there is a scope of, of things?
	(Nurse) For example like in every room, you find for example if it's incubator settings depending on the baby's weight, if it's how to do kangaroo mother care and the benefits of kangaroo mother care, if it's like neonatal resuscitation, the steps that you are supposed to follow.... Yeah, we have charts.
	(Nurse) I'm saying, like the work instructions, most of them are on the walls.... They are there in the walls. If it's doing a balance score, we have those charts on the walls. If it's doing the cooling therapy the charts will show you the minimum temperature. We have charts all over in the unit for different things.
	(Nurse) we have a, nini (whatever), charts, heh, on the walls,.... yeah. Only those charts, as well as we've got some guidelines in the unit.... yeah, MOH approve
	(Nurse) aah, the charts. We've got 'speaking walls'. I.. 'speaking wall'..... yeah. Those are the charts, the guidelines like standard management of neonatal pneumonia, neonatal sepsis,... charts on Apgar score, charts on fluid management,... yeah. Kangaroo care.
	(Nutrition Intern) there was, there was, there are this charts that are put in NBU. What was it grading? Is it the z-scores? There, there are just very interesting things in NBU... especially at the resuscitaer, there is something that they use to score, is it, something score, am forgetting the name of that founder, yeah, but there is that score that I found very fascinating and I just went to look it up like how do you assess this, how do you assess this, and, in fact the people in NBU there was a time we had a case that just came in and I just approached one MO and I was like, what do you mean when you people just calculate the z-score and go like, oh, this score was 8. Is 8 a bad thing or a good thing and how do you grade? How do you get to 8? And they were very open to teaching and they were like, so this is how we score, and we scored a baby together and I was like, that feels good...it actually feels good, but there was never group discussions
	**LN17: When there is a lack of structure to meetings and other communication formats (C) information will not be shared effectively (M) leading to staff frustration and lack of unity (O).**
	(Nurse) Late communication, is always communicated like, you'll find that we have a CME tomorrow, so to factor in in my plans is sometimes difficult. It's always like impromptu. It's not something that is planned like next week on this this day we'll have a CME on this and this topic. So, if I'm just told there is this CME and you don't even know the topic, yeah, it becomes difficult even to plan for it.
	(Nurse) I think if just proper channels like timely, timely time, timely kind of information. If a person goes for training for example, they give feedback immediately. So, I think that will improve the quality of care. And also, things like information from the top management to the department should also be done in a timely manner. So, timing is everything for me........They are not adequate. Like

	we need to have more trainings, we need to have more CMEs because we only have a CME once in a week and sometimes you might not attend that CME, you are not at work, so you can't come from home to attend a CME, it's hard. And also, there should be like those many charts on information, charts and posters, such things can improve.
	**LN18: When training is included in a CME (C) staff will consider the new information to be legitimate (O) because it has been validated by the hospital/ unit (M).**
	(Student Nurse) Okay, if, if someone let's say finds something new, I can give for example, cooling therapy, they have to, they have to give a CME on the same CME
	(Student Nurse) Yeah I see… I usually hear people around CME sometimes you might not see people and then you hear they are on CME.
	(Nurse) The person who is ready for that, normally. There are some we say this thing, they have changed the protocol, but he's not getting what you're saying. So, there's is somebody who is very much, very much in need of that, you can share with that person. So, we can't just say it to anyone else, because some they don't want new things from you, they went to go to the CMEs so that they can get the knowledge. But something you have read it has changed, you say you have read the protocol, it has changed, this thing, they do this and this. He's not going to take that information because she or he can tell you, "Have they called CME to change that thing?"[Laughter].. We need a CME for that.... Yeah, yeah, they want to be called, to be told the protocol has changed, management of this has changed this, you see now?.... People prefer the CME more than just from the internet or such...... Just the culture I think, because they need CME. CME has to go round everybody to attend, that's what they want.
**Allocation of learner to teacher**	**LN19: When staff deliver ad-hoc teaching to students/ juniors based on opportunity (C) practical on-the-job learning can occur (M) improving satisfaction of learners (O).**
	(Nurse) I think uuhm, but it's not a routine, maybe once in a while, we get time where, an in-charge teaches students.... so, for those who are not there, we're told to pass the information.
	(Resident) Yeah. we used to also have some teachings in the newborn unit. So we'd have some skills teachings, some skills teaching like for intubation, giving surfactant... that would be given to us by the fellows or the consultants.
	(Student Nurse) So, for the doctors, most of it we were observing the procedures. Like for example, the intubation, the tracheal intubation process, we were also, sometimes you could go and check how they do surfactant; that, that is a procedure. So while they do, they explain to us, you learn and at the end of it, we again, we gain knowledge and experiences, yes.
	(Student Nurse) …. after doing the days or the shifts duties, she would sit you down and like tell you, today let us discuss this..... this condition or let us discuss this procedure.... call us as a group, yes. Actually, she's the only one who would take that initiative of calling all the students as a group. Like, today I want you to learn something... let us discuss CPAP or let us discuss phototherapy.
	**LN20: When students/ junior staff are allocated to a specific staff/ senior staff (C) supervision is integrated into delivery of patient care (M) enhancing learning and sense of belonging (O).**
	(Resident) Students, yeah, the students that I'm allocated with, because you see the students are usually very many and our unit is very busy. So the students that you are allocated with, obvious you will guide them, so you will be in constant communication with them.
	(Student Nurse) Then you'll tell the nurse maybe you are allocated together, the nurse you are with, this baby is not well maybe they could just increase she could… the baby to be put on the photo for some time because the jaundice is still there, and you'll find that the nurse will take your… will take into consideration and ensure that the baby was put on photo.
	(Nurse) We just engage the students in our work. We engage them. If you're doing something, you make sure that you, you, you involve them in your work. ...Like, if you are, if you are top tailing, you top tail with them, after finish, after doing it together, you assign them a baby to top tail, so that, to ensure that ... So that, to ensure that they've learned.
	(Nurse) when you are allocated student, you do communicate. Like you instruct them what's supposed to be done, how it's supposed to be done, and also you, you observe them on what they're doing. Try to correct. Try to encourage something they've done correctly.... We do delegate, maybe preparing of fluids, doing vitals. Yeah. Such things.
	(Student Nurse) So in in our case, we used to have a licensed, a registered nurse in this specific cube you are allocated to, so that will be our supervisor at that level.
	**LN21: When students/ new staff are supported to undertake duties/ procedures relevant to level of training/cadre in psychologically safe environment, e.g. be "hands-on" through return demonstrations (C) they will gradually build their self-confidence through supervised practical learning/ exposure (M) and will become competent (O).**
	(Student Nurse) The, the primary nurse, who you've been put up with, supervises you
	(Student Nurse) I'm saying, if there's a procedure, a nurse can just begin the procedure as you observe, then if the next baby will need the same procedure, it will be a return demonstration.
	(Student Nurse) first time I used to observe... The second time, we used to gather the things in the trolleys preparing the trolleys for the procedures to be done. .. Then after that I started now thinking I could do it and I tried first....Most of them I couldn't make but as time goes by I was good in it and I was okay.
	**LN22: When students/ new staff receive supportive supervision (C) fear of the intimidating/ unfamiliar clinical environment is mitigated, e.g. small babies (M) improving confidence and competency (O).**
	(Student Nurse) The fact that you were being, like, the fact that we used to do like hands on. That one, like it would, you know there was a time we were new in the unit, you could just see a small baby and you are like ..... they are putting on her NG, but now after staying for a while, you are doing the NG, you doing the feeding, so, you are like, "So, I can do this." So, the confidence is build.

	(Student Nurse) Yeah. I can say all of them, most of them, if not all, like they had to, they, they're the way they used to think about these small kids, that they're so fragile, they can't, as, you can't handle them like this, like they, right now I think they're more confident on taking care of these small, small babies.
	(Student Nurse) Yes, That is something I can say, (name of hospital) is doing best is our student? To have confidence in themselves and that part I can give them 100%, you see we come here with fear but when you are exposed to the same situation in week one, week two, week three, this one is doing this, that persistence gives you confidence. So, when I leave here, I know I will be a more competent nurse than how I came here.
	(Nurse) I can say, you see when, when, when people are new, when the students are usually here, they usually don’t want to touch anything, not unless you show them because they are afraid maybe these babies are too little, they are fragile, and they don't want to mess. So, they usually ask first what, we show them. If it’s like inserting a tube, they tell you, “Sister, show me how are we supposed to insert because we don't want to mess with the baby.
	**LN23: If staff are overworked (C) they may feel that students are a burden (M) and may not be able to adequately teach them (O).**
	(Nurse) Because now some of us we are busy. That one requires somebody who is just free to teach them on most of these things. Like our ward we’re very busy, we just tell them one, two, three, we start medication, we start this and this, we don’t have enough time to teach them. It requires a lot of time. By the time you’re finishing your work, it’s already 1:00pm. So, there’s no enough time and that’s why I said they need that mentor to come to the ground to teach them well, to demonstrate some of these things, it requires a lot. So, you are two of you on duty, you can’t get enough time to teach these students, to support them.
	(Nurse) ... aah, basically, we never used to interact that much... coz they had a lot of workload..... they had a lot…. they are either writing.. inserting IV lines...
	(Nurse) heeeh! Sometimes it’s hectic, let me say that... sometimes it’s very hectic,... because, you see, when a, you’re given like, 7 to 8 students, and they’re green.. so, you have to teach them one by one. So, it means, that day, you’re teaching,.. and you’re giving care..... so, you’re tired double.....yeah. Heh! It’s hectic.
	**LN24: When there is inadequate clinical supervision (C) some students will practice beyond their competency (M) leading to poor learning, cover-ups, risk of patient harm, more work for staff, and student second victim (O).**
	(Nurse)... for the students, it’s not that much, students are given like the leeway, as in, it’s almost same as just staff, in that, in that the supervision is only to ensure that they’ve name tags, uniform, they report on time, they leave on time.. but, basically on a clinical aspect, there’s no supervision that much.
	(Student Nurse)... I think, the first week now was a bit shady shady.. coz we’re not nini (what), we are not, you know what, we are not what? We’re not yet knowledgeable.... in the routine. But once we got the hang of it, I would say, we improved the quality of care of those kids. Coz, you see we are many,... we are sure that each and every child is looked at, is taken care of, as opposed to like when they’re alone, like, at night, it felt like maybe this azimio babies, these kids who are homeless.... nobody shughulikiad (took care of them) them, coz the nurse was one... mmmh, so I’d say, we improved quality of care.
	(Student Nurse) Delegate and go to the tea room. And once you just do something wrong, they'll come and reprimand you. And yet they are not around to guide you do these, these and this.
	**LN25: When there is low staffing (C) supervision will suffer (O) due to patient load (M).**
	(Student Nurse) But some other nurses, their tendency may be the supervision is not much, and I do not know whether it is the shortage of staff. Because I am assuming when you get into this room, I have like 13 babies and I am just reading the notes and I so this nature (short staffing), as much as she wants to supervise you more, it will be, it also becomes a challenge.
	(Senior Nurse) The first year student, first year student. So that one you find, most of the patient care, they don't know. So you have to be there to monitor, to teach. And sometimes there's shortage. You can't, you don't have that time to follow student to teach. So you find that it gets to a place staff neglect them, now I will waste time, to train, I don't have time, I do what I'm supposed to do, let them stay there, maybe I'll train them on their OBs, so, that they can do the OBs.
	**LN26: When students feel supported by staff (C) they will feel at ease (M) improving satisfaction (O).**
	(Student Nurse) Like she, was very understanding to the students very much. I remember there this time I didn't show up for the shift because of some reasons, I couldn't avoid, but now my mistake was I didn't communicate to, to her directly, I sent a student, but she never delivered the message. So the next day, when I came I explained to her, but she didn't have an issue, she was like, it's fine.
	(Student Nurse) Yeah, most of them, it influenced them because of the working environment. Cause we had access to everything, and also the nurses were supportive. You know we had much access to information, now it was kind of positive to us.
	(Student Nurse) The have very good listening skills...when you are not getting and they are explaining maybe the work was too much, ... they tell you go for a break then after that you come back and maybe you’ll start your work more with a fresh mind, yes... Yes, considerate. ...About them they were also during break time or tea breaks... during maybe we are documenting they will tell you why, they educate they tell you why we do this, this is how you do it and explain to you why we do this way.
	**LN27: When there is an assigned clinical instructor (C) students know they are monitored (M) setting an expectation of learning (O).**

	(Senior Nurse) …. students who come with the ... with the clinical instructor, they know that here we must learn. So if students are followed from school, they will learn. If they are not followed from school attitude, attitude, that I'm just passing by. And especially for those ones who don't have exams. They know, I'm just passing by, if it's work, I'll do it once I qualify, here I'll just pass by. So you get that these students, sometimes they don't learn.
	(Nurse) ... they usually come with their lecturer....Yeah. Clinical Instructor, their clinical instructor. Yeah, she's usually, she's usually here...
	(Nurse) The…the…the students they have a clinical instructor who always have eyes on them and there's also a nurse from the unit who is also allocated to deal with the students. There is also the doctors and the primary nurses in the rooms who also guide them... The primary nurse supervises… they…they allocate the student to patients; they tell them what to do.. they… they also supervise when they are working... so that they are sure that whatever is being done is the correct thing. Yes, and they also teach them a lot.
	**LN28: A clinical instructor (C) can enhance student learning (O) by setting expectation, admin and pastoral care, point-of-contact, induction, practical training in procedures (M).**
	(Student Nurse) The one I valued the most was our instructor, student instructor..... Because you see we have manual, our student manual is there, so it's like we refer to the student manual. Like likewise to her. So, I would prefer an information coming from our instructor, yes, and even the in charge.
	(Student Nurse) Yes, Clinical instructor. She is the one who introduced us today to the unit. The way she tells us to behave the way we behave, but with time you come to learn different behaviors from different primary nurses and primary doctors then you get the right behavior.
	(Nurse) … you have the clinical instructor f…..She takes them through a group. ..Yes, in case they have challenges, something that they need to be taught. They have talent. .
	**LN29: When mentoring is successful (C) it is valued by both mentors and mentees (M) leading to satisfaction and personal growth (O).**
	(Resident) We are being supervised by our seniors. Most of the time the fellows, the consultant, yes. Let's say a more of a mentorship kind of a program, yes, and that's observation with, yes. So that is the main thing, yes. And most of the time you the, the fellows and the consultants, so they will mentor us, they will teachers, they guide us, yes, kind of a family.
	(Consultant) ...Very fulfilling. The best thing is to see your mentees grow, become significant… having them make significant strides, even better when you see you have made a mark in their lives. I think that is the most fulfilling thing. I have had the privilege to have at least people I have mentored, and years later we later met and they told me 'had you not told me…' it is very fulfilling and it takes you even through the rough patches (laughs) and it makes you say, 'i am not all that bad, I have grown some good ones…it was not in vain after all!
	(Nutritionist) it's quite a good experience... yeah. Am, am always happy when I mentor somebody,
	(Resident) Okay, me personally, yes. The paediatric unit had allocated as mentors as we started the program. So,.... Not fellows, they are consultants.....Yeah. And I would say my mentor is good, because we've met about three times to just discuss my experience I made and what my plan is and what career, yes. So, I think it's good.
**Student initiative**	**LN30: When students are proactive (C) nurses will be more likely to teach them (O) as they do not have to spend effort rallying students when they are already overwhelmed with clinical work(M).**
	(Student Nurse) mentoring, I would say, mentoring? Okay, a little, a little bit..... I don't know if they're overwhelmed or,..... that's why they don't mentor as such..... yeah. But if you'd ask, you'd get, but they would not take the initiative to...... but maybe it's because they're overwhelmed or something..... the support is there, when you ask for it. ....it has to come from you, yes.
	(Nurse) ... And also it's the student's initiative to be able to want to know what they'd want to know. So I feel like the nurses are always willing but now it's, it's upon the students because you are overwhelmed. You can't work and still try and mentor somebody. And even them, they're not willing to even ...
	(Nurse) Yeah, you tell me sister I want to learn how to give medication. Sister I want to know how to put an injection. I won't be telling you that now because I'm working. I'm supposed to teach you on job. If I am doing a procedure and you decide to sit down or go outside to receive a phone call, I won't come looking for you to find you and teach you.
	**LN31: When learning is self-directed and based on personal initiative (C) students and new staff will be proactive, or observers, or do the bare minimum (M) leading to spectrum in learning – from hands-on and ready to work, to not learning quickly, to avoidance and no learning (O).**
	(Student Nurse) Yeah. But if you are the kind of person who, you, you know what you came here and you want to gain something from the experience, you'll just go to ward rounds, even if you've not been allocated there, like it was your own initiative, if you don't have that initiative, you'll come take the vitals and leave, the next day vitals, you go.
	(Nurse) Most of them are green, somewhat like okay, some will find that they're willing to learn others.... Okay. I wouldn't mention the institution....There are those students who would come, who are willing to learn. Others are busy dodging, for those who are willing to learn, you know, at least you, you know, by how they want to assist you in doing something, they're teachable. You'd carry out procedures together. But some, when it gets to another time, it's almost end of shift. They just, like, they won't even tell you they left, you just find them missing.
	(Nurse) For the students, the challenge is some don't want to learn, some are willing to learn. Some come, clock, they just loitering in the unit. Others come straight and they want to ask and learn. There are different aspects.
	(Nurse) ... like the students, si I said there are 2 types of students. There are students who are good and there are students who are just there for the rota to end... so, if you find these good students, today you teach them, tomorrow you're with them, and after 2, 3 days, you see a very big difference, they're doing the right thing.

	**LN32: If a person is not motivated to change, or their motivation to attend a course is not primarily for learning (C) training received will not be implemented (M) and no change will occur (O).**
	(Senior Nurse) Sometimes I went to the training because I wanted to get off the ward, I was stressed by the in charge, so I applied to go for nephrology, but my passion was not for that course. So, I went and studied it, now I'm through, but I don't want to work there.
	(Senior Nurse) I think, for those people, they have positive attitude, you will do it, you will apply. But you know there are other people who come from training, it's like they did not even attend anything. And others just, they continue practicing their former practices than, than whatever they've acquired of which they should be bringing a change.
	(Nurse) So, like personal attitude is like if that person feels like.. me ...I don't want to do it now...they keep on procrastinating, I will do next time, those are things personal to a person.
EQUIPPING	
**Physical environment intentionally enabling**	**EQ1: Where there is a low staff: patient ratio (C) overwhelm of staff (M) will lead to shortcuts, workarounds, missed care, mortality (O).**
	(Nurse) Workload, when you're alone in a room with about 10 or 20 babies and their mothers you don't really have that time to really check on the mother the way she is breastfeeding and all that because you are also having, among them there could be a…a very sick one whom you are concentrating on. ….. So the only way to sort out that is to link the mother who requires any assistance with the with the nutritionist. Otherwise as unit, as a nurse you are not able to do it and maybe later as you continue not doing that you kind of forget.
	(Nurse) aah, for our unit, we are sixteen... so currently we've got like 3 on month leave, so whenever we find ourselves like, whenever one falls sick, like has a sick off... there's a massive shortage. You find ourselves like one nurse taking care of 60 neonates. Definitely as much as you try, you won't give quality care because of, that large number of neonates. You can't give quality care.... coz of, you'll be distracted. As in, you'll have to divide yourself into, into 60 parts
	(Nurse)... But you might find yourself you're so busy with everything with you like you have so many babies. By the time you start your management for this one, you go to this one, you go to this one, and when you go to the other one who was prescribed something else, you have not done that, maybe that, due to overworking, that can make or really hinder you not to do what is right
	**EQ2: When there are insufficient staff and the unit is busy (C) managers may perform a patient care role (M) and neglect managerial tasks (O).**
	(Senior Nurse) But most of the time it is burnout and time and shortage so you don't even… you can't even create an opportunity to come and sit down and discuss with your staff.
	(Senior Nurse) …… most of the times I do hands on and in fact I don't, I rarely do managerial work [laughter].
	**EQ3: When equipment is insufficient on the unit (C) workarounds will be employed (O) as best practice is impossible (M).**
	(Nurse) For example, okay, for example, if, if you have, let's say five babies who are jaundiced, you need to, they need, they need phototherapy and you know very well, they phototherapy machines are few. And you're forced to use one machine to be shared by, let's say, five babies or four babies, you know very well, they'll not be getting enough
	(Nurse) Probably in the use of equipment's, for example, CPAP. There is a time you find that the, there are missing parts so that we can get a complete one. So you get someone telling you, by the way, it reaches at some point, if you go, someone tells you, if you missed such a part, use this, it's an alternative, and you do exactly that, and you see it works.
	(Nurse) As in, you see in class you learn too much theory. But now, in the clinicals it's the, it's like the real… Yes, the real situation. Maybe in, in class, for example, if it's inserting, like a certain procedure, the way you are supposed to set up a trolley on the top shelf, the bottom shelf, but now you come here we have no even those trolleys, so you have to improvise whatever is there to perform that procedure.
	(Student Nurse) Like us not having the VE packs you have to improvise by using the patients cotton so that you can do a VE instead of doing with the the sterilized equipment's.
	**EQ4: When equipment is not readily available (C) staff will feel frustrated and may display blame/ incivility (O) because they cannot provide the care they want to and undertaking their job becomes very difficult searching for everything (M).**
	(Nurse) With that also to being unrealistic, some things are unrealistic. Like you're being told now there's a certain medication, you're supposed to use a pump, and it's supposed to go for one hour. The pumps, first of all are not there. And now giving this medication with a pump, it, it's, it's just impossible.
	(Nurse) That's what we are told to do. But you don't have enough. You must have boots, goggles and proper heavy gloves. But we don't have them as part of the organization. ...We've raised the issue, but they're saying that use whatever you have in place. It becomes difficult for us. But just try your best, that you don't get contaminated.
	(Nurse) Yeah. Like I mentioned earlier, is lack of basic things, basic things like you can work, you can come even to duty and you find there are not even gloves, there are no syringes, there are no basic drugs. Yeah. And so, those are first line. So, if you're coming to duty, you're having to give medication, there's no medication, there's no syringes, so you feel like, "Have I just come to document that baby failed and retained?" So, yeah, and even the oxygen is not there. So, sometimes you even tell them, "Why can't you just close the unit, when you get this thing then we come back to job?"

	(Nurse) Two, the negative also part is that sometimes we can come in labor ward then the first thing you're told when you enter is there is no gloves, so you're like, how am I going to work? So equipment's, may be today there are gloves then tomorrow you find that there is no gloves, today fluids are there and tomorrow they are not there, so equipment's tend to fluctuate.... So it kind of makes work difficult. You, you want to help out but you cannot, so equipment's also have stress.
	(Senior Nurse) What another thing the negative part is when you lack supplies....Yeah you strain looking for this supply its hard...... But when you have everything in Place at least you are motivated. I think even in motivation is when everything is available. Whatever you want is available.... It will affect you because you will not want to deliver what you want to your patient.
	**EQ5: When equipment is kept orderly (C) staff will be able to locate what they need for smooth working (M) leading to greater satisfaction and smoother working (O).**
	(Student Nurse) Another thing is the environment by the way. …… because if you see how they are arranged, they are washed, they are cleaned thoroughly, and it's even appealing to the eye when, when, when, when, when you go into that maybe one of the cubes; you just see by, for yourself. Yes.
	(Nurse) I should be able to make them be near them.... Available, so that they don't miss that. So when you come before you even… once you receive the report for me the first thing is usually take the things I know they will use very fast, even without a trolley, very fast put them there like suction, suction catheters I make them available and the gowns for wearing and then now I can come with my trolley and after I have done a quick check in that trolley, in the trolley where we put the consumable, what is really missing completely. If there are no gloves completely and the…there're no suction, I'll put them very fast then I come and now equip the rooms properly.
	(Nurse) Me I am one person who likes about my safety then patient safety. If my environment is safe..... Yes, normally I do my own arrangement I know everything is ready before I start my work. Yes. So in case the information actually I want to ask is… be prepared for anything. When you come to workstation ensure your work environment is well equipped, arranged… so in case you have an issue you know where to get whatever you need.
	**EQ6: When equipment is poorly organised (arranged, stored, labelled) (C) it is difficult to locate the item (M) leading to workarounds (O).**
	(Nurse) Yeah. So you may ask and you look for it, you can't find it. And maybe somebody knows, maybe they're aware they're not in supply in the hospital, or they are they are placed somewhere maybe where we don't know. So sometimes you can miss such kind of information. …….. And somebody who knows maybe where the chart is or where the file is kept, it has been misplaced. So you may get, sometimes you may get something missing.
	(Clinical Officer Intern) You just use what is there, sometimes you find the strappings are not there you are just there you want to canulate there is no strapping.... you go asking from other wards. Sometimes either the cannulas that we use are finished.
	Observations
	**EQ7: When the person who knows the location of equipment is not around (e.g. at night) (C) staff will be unable to locate it (M) and feel overwhelmed (O).**
	(Nurse)... you have to go and borrow from the other ward, (chuckles).....if, they don't have, then that's all. (laughs)
	(Nurse) Yeah. So you may ask and you look for it, you can't find it. And maybe somebody knows, maybe they're aware they're not in supply in the hospital, or they are they are placed somewhere maybe where we don't know. So sometimes you can miss such kind of information.
	**EQ8: When an item is considered protected/precious (C) those who are considered to be overusing it will be blamed (M) leading to incivility (O).**
	Observations
	**EQ9: When an item is scarce and in high demand (C) staff may shout for it (O) because this is the norm (M).**
	Observation
	**EQ10: When physical areas are designated for specific functions (C) this will improve working processes (O) as staff will expect those tasks to be performed there and know where to find the necessary items/ paperwork (M).**
	(Nurse) You, you need to bring to bring them close, so, okay. I make sure they, they're well oriented to the place. Yeah. This is what we do. This is what we do, this and this when you want this, you find it here, such things. Just making the environment conducive for them.
	Observations
	**EQ11: When the physical space in the unit is overcrowded (C) staff will find it more difficult to carry out their roles (O) due to lack of physical space to undertake duties effectively (M).**
	(Nutritionist) I've just said that colleagues who don't exercise teamwork as you say, you get in, and you see this shift, no, this will be a long day, or an environment that is not conducive. Like for now, I feel with all the things happening here, maybe they should consider expanding this place. How? I don't know. But it's not the best with that crowding.
	(Nurse) Like You can't neglect baby because there is no space but at the same time the infection prevention is…its gonna be really hard like maybe they might share infections...... Increasing the like maybe bed capacity. Yeah.

	(Nurse) ....cleanliness.... the place wasn't that clean. Maybe it was, it got washed once a day.... and it's congested
	**EQ12: When staff feel collective responsibility and sense of belonging (C) they will contribute to the order of the unit (e.g. clean, tidy, organise) (O) as they recognise the contribution of an orderly environment to provision of patient care (M).**
	Observations
	**EQ13: When patients are grouped in the unit according to their clinical needs (C) staff can be more appropriately allocated (O) as skills mix can be tailored to requirements (M).**
	(Nurse) OK. OK, I speak to colleagues when I am working, especially knowing NBU we are divided into rooms, and everyone works in their located area. So. For me specifically, I work in the NICU, and the Colleagues that I speak to are the ones that are located with and. Of course, the doctors that we are also allocated within the same room we I speak to them. I also speak to the team leader who comes to ensure that everything is running smoothly.
	(Nurse) Yes. They in different room, ……. That's how the rooms have been divided. And also in terms of now, how they have, how they have, how is it called? How they have, what they are, they have narrowed down into, because they are neonatal nurses, they're also ICU nurses.... Specialization it is.
	**EQ14: When items and equipment are arranged/ positioned intentionally on the unit (e.g. different colour bins in same location, hand sanitiser on trolley, labelling of cabinets, gowns for mothers, etc.) (C) desired actions will be implemented (O) because 'the right way becomes the easy way' (M).**
	(Nurse) Me I am one person who likes about my safety then patient safety. If my environment is safe.... Yes, normally I do my own arrangement I know everything is ready before I start my work. Yes. So in case the information actually I want to ask is… be prepared for anything. When you come to workstation ensure your work environment is well equipped, arranged… so in case you have an issue you know where to get whatever you need.
	(Nurse) I should be able to make them be near them.... Available, so that they don't miss that. So when you come before you even… once you receive the report for me the first thing is usually take the things I know they will use very fast, even without a trolley, very fast put them there like suction, suction catheters I make them available and the gowns for wearing and then now I can come with my trolley and after I have done a quick check in that trolley, in the trolley where we put the consumable, what is really missing completely. If there are no gloves completely and the…there're no suction, I'll put them very fast then I come and now equip the rooms properly.
	Observations
	**EQ15: When there is a unit telephone (C) communication between internal and external staff will be enhanced (O) as unit staff are empowered, and more accessible (M).**
	(Nurse) mmh, I think there should be a telephone in every department.I.... like they are inter-connected..... I think there should be an information desk....... communication? For there it's just a notice board, and these posters....and having CMEs in the unit
	(Medical Officer) So we used to have that barrier which has been a bit resolved now coz we have a phone in the unit. So we have an NBU phone. So if labour ward is bringing like a preterm they tell us in advance. So we had that issue where suddenly babies were brought and we didn't know who they were.... But that has been improved recently like I think it's been like three or four weeks we have a phone.... So like referrals call now to the NBU not the hospital covering. .... Coz sometimes they used to call the hospital covering. Then the covering has not reached us to tell us such a baby is coming..... but now they call NBU directly. If the covering is called they call us on the NBU internal phone. ... We have like a phone for the NBU......so I think it has improved communication skills. Yeah.
	**EQ16: When the physical environment appears to require particular actions, e.g. waiting area, signing in book, hand sanitiser with sign (C) desirable actions are likely to follow (O) due to communication of clear expectations (M).**
	Observations
	**EQ17: When different cadres/ sub-groups sit at different tables (C) boundaries are enhanced (O) as identity/ difference/relative power is demonstrated (M).**
	Observations
	**EQ18: When physical information is accessible, specific and clear in function (e.g. manual, communication books, guidelines, handover book) (C) it is a useful reference source (M) to assist staff/ students in their work (O).**
	(Senior Nurse) book in the unit. Where we again, document anything apart from, we are CME is about the teaching, but any communication, sometimes as the ward manager, we are called for meetings, the departmental meetings and we are briefed about the hospital and what is happening. So we are given knowledge from those meetings, and we come at the next nurse's desk and communicate, give the information what is happening, what is our expectation. And whatever we communicate, since it can't be able to reach all of us, we put it in the communication book.
	(Nurse) Okay, what there is, we have the protocols of management of a baby. Of course, even if you get maybe advice from me, we always refer to be sure..... Yeah, we refer maybe it was a management of a certain coordination everything is in that protocol you can discuss and maybe after whatever is settled we also go to get more information... I will do it this way but when I get time I go to the protocol and try at least to peruse and see are we on the right side or there is something we are missing.

	(Nurse) .... there is a, what do we call? there is this book in NBU, there is a book in NBU about paeds. I can't really remember the name, but there is a book. Stating drugs... different types of medical conditions.. and their management.
	(Senior Nurse) Yes we have a protocol....That is what guides us in the care of our babies... Yes we have a thought the neonatal protocol is not yet out but we have some things but currently we are using the paediatric protocol.... But we have some soft copy for the neonatal we are not we are not yet having a hard copy.... So we are have protocols there we just don't work. We are guided by protocols....This protocol can guide that's why even a nurse can act in the absence of a doctor or a clinician or whoever because the protocol will guide you on what to do. At least you will not be left without a knowing on what to do... You refer to the protocol and act.
	**EQ19: When posters/ protocols/ guidelines/ SOPs/ job aides are posted on the unit walls in strategic places (e.g. incubator guidelines on wall next to incubator) (C) they provide a trusted, clear, timely reference source and reminder of what should be done (M) and improve ease of work, continuity, and student and staff learning (O).**
	(Nurse) Yeah, they are, there are work instructions in every room, they cover so many things. Maybe for example, let me start with …., the, the feeding, the feeding schedules that means the baby, those babies with tube feeding, cup feeding, breastfeeding. Okay. There is the kangaroo mother care…. there is schedule on measuring or regulating humidity in the incubators. and there is a scope of, of things?
	(Nurse) For example like in every room, you find for example if it's incubator settings depending on the baby's weight, if it's how to do kangaroo mother care and the benefits of kangaroo mother care, if it's like neonatal resuscitation, the steps that you are supposed to follow.... Yeah, we have charts.
	(Nurse) I'm saying, like the work instructions, most of them are on the walls.... They are there in the walls. If it's doing a balance score, we have those charts on the walls. If it's doing the cooling therapy the charts will show you the minimum temperature. We have charts all over in the unit for different things.
	(Nurse) we have a, nini (whatever), charts, heh, on the walls,.... yeah. Only those charts, as well as we've got some guidelines in the unit.... yeah, MOH approve
	(Nurse) aah, the charts. We've got 'speaking walls'. I.. 'speaking wall'..... yeah. Those are the charts, the guidelines like standard management of neonatal pneumonia, neonatal sepsis,... charts on Apgar score, charts on fluid management,... yeah. Kangaroo care.
	(Nutrition Intern) there was, there was, there are this charts that are put in NBU. What was it grading? Is it the z-scores? There, there are just very interesting things in NBU... especially at the resuscitaer, there is something that they use to score, is it, something score, am forgetting the name of that founder, yeah, but there is that score that I found very fascinating and I just went to look it up like how do you assess this, how do you assess this, and, in fact the people in NBU there was a time we had a case that just came in and I just approached one MO and I was like, what do you mean when you people just calculate the z-score and go like, oh, this score was 8. Is 8 a bad thing or a good thing and how do you grade? How do you get to 8? And they were very open to teaching and they were like, so this is how we score, and we scored a baby together and I was like, that feels good...it actually feels good, but there was never group discussions
	**EQ20: When a computer is physically available on the unit (C) staff may use it for information (O) as they can access online resources (M).**
	(Nurse) Maybe if you read from internet, modules... Yes, or the computer that is there.
	(Student Nurse) Outside you would go for books and you google because you are not allowed to use phones in the NBU. ...Yes, but you could use the computer for the nini. [the unit]
	(Nurse) Well, there are several ways of getting information disseminated into the unit, because we have computers that we are having in the unit. Through these computers, we have email addresses, WhatsApp information, so we keep on updating each other about all what is happening within the unit. So at any given time, if you go through the computer, you will get some update and WhatsApp group information
	**EQ21: When staff feel comfortable with a colleague (C) they may directly call them on the phone (O) because they want to get immediate information (M).**
	(Nutritionist) ...my first choice is phone call... that is my first choice. .. if I, it's like, it's like…. one on one,I.. yeah. To ask for that help, and guidance... then eeeh, sometimes if the phone call is not received, I'll follow it up with ... with a text.
	(Nutrition Intern) I think, for when I need information and there is, and there is, the immediate person cannot really help me, and my in-charge is not around, I end up calling....I just make a phone call and am like, there is this patient…., I consult over the phone and then I get help. There is no time I've inquired, and they were like, no hata mimi sasa hapo sijui tutafanya aje (even me in that situation I don't know what we will do), and then we were all there. More often than not, I get the help that I need, even if it means making the phone call and consulting.
	**EQ22: When staff/ students use their phones as a tool at work (C) they can access guidelines (M) to assist with clinical tasks (O).**
	(Resident) Yes, I do, yes. We use internet so many platforms, yes, to check and see, what are the new things, what do I want, what do you want me to read about it? Yes.... Sometimes if possible. If you want to check the correct dosage of this medication, yes, it's possible, yes.
	(Resident) Yeah, yeah. I remember my colleague would tell me, you know, maybe there's this book that we were, people have on their phones, it's an app, so she was able to tell me to go download it and I did and it really made my work simple. Because now it was an app in the phone and any drug that I want, I can just get it, because now the other times you be like, "Oh, what's the dose of

	this? What it the dose of that?" Now that I have it in my phone, it became so much easier and I was like, "This is good." Yeah. And I would also want to share that with everyone, "Oh, download this book, it's better, it makes saves your time."...
	(Nurse) …you go you check in the book; we have some books there what is the name? in the unit… in the phones again......Yes, yes, neonatal guidelines.....Yes, so you have them. Yeah they are in the… they are in the phone soft copies yeah.
	(Nurse) Most of the time I use Google. When you are stuck, Google is the right.
	(Student Nurse) I usually go to Medscape.
	(Resident) ...internet?... Initially, very often. But then, as I went along, and learnt all the protocols like where to get all the necessary things, then I just had an app....There is one app for neonatology.
	(Nurse) OK there is also those small books... they are called booklets?.... Yeah, so like there are those guidelines. We were told everybody at least to download, to have a soft copy. So, I think most people have them on their phones, so maybe you can just refer them.
**Orderly storage and use of clinical records**	**EQ23: When there is insufficient supply of stationary for patient records (C) staff will use workarounds (M) which will exacerbate disorder (O).**
	(Clinical Officer Intern) I prefer the printed printed… stationaries Because we them, at least you can't. because you know… So you know the they are you are just filling... It's like a Q and A just filling so there's no way you can leave a blank page because even you yourself why have I ever left this page blank? ... Yeah. But you see with the plain paper, you can't… it's not that easy to remember let's say everything that is supposed be the way the standard way... You will end up missing some parts.
	Observation
	**EQ24: Poor quality written records (e.g. incomplete, multiple, duplicated, missing IP numbers, etc.) (C) lead to distrust of their accuracy by staff and families if used as a reference source (M) causing compromised patient care and dissatisfaction (O).**
	(Student Nurse) ... the student did not sign.... so she gave and did not sign. But you see in nursing, if it's not documented, it was not done! ….……..(so, it happens that), there were 2 treatment sheets again! For one baby.... she signed one, another one was in the file that is not signed....exactly, this one comes from the, the COs, they just write the, the doctors, they write these things, they hoard them.. ikishapotea, ama (when it's lost) they don't even confirm that it is lost. They write another one.....
	(Nurse) .....poor! Poor! I even mentioned. People don't even sign T-sheets. Mtoto anapata dawa, hujui alipata jana, hakupata jana, hazijasigniwa (a baby gets medication, you don't know if s/he got yesterday or s/he didn't, they have not been signed)! Nobody cares, life goes on.
	(Student Nurse) .. Yeah. You know sometimes because of the paperwork; you can misplace some papers.... Yeah, you can ask the primary nurse but when you go through the file, you can find that it is the paper that was written that is missing.....If it was, if it was, if it was history, you just come and take it from the mother..... You go back to the mother.
	**EQ25: When written records are integrated into verbal exchanges/ handovers/ taken to bedside (O) their value and purpose will be enhanced and expectations communicated (M) and quality of records will improve (O).**
	(Senior Nurse) Those ones I talk to them frequently, because most of the time I go to the bedside, and that is where I meet them and we deliberate on patient management, on documentation, on how better or they are feeling on the bedside.
	(Nurse) Yeah. When the doctors do the ward round, whatever plan they have said... they've discussed or they have decided, they normally write it on the file, so you as a nurse...definitely there's a round book you will write. You write on the cardex, then you will implement whatever was written.
	(Consultant) So I'll take for example, handover. Handover is actually written down. So you can read about the handover at least from the nursing perspective… You can read on the handover and be able to take over. …….. Just the same way with the medical officers who are writing notes while I'm leading the wardround… if you do not note that I am the one making those decisions, later on when you are being asked 'who made the decision?', you can't even remember because you did not note down. So incompleteness. There are times we actually do what I call a 'paper around' just to make sure that documentation in itself is being adhered to.
	**EQ26: When quality of written information is poor (C) staff will find workarounds to gain necessary information (e.g. handheld notes, verbal exchanges) (M) thereby further degrading value of written information (O).**
	(Student Nurse) Yeah. You know sometimes because of the paperwork; you can misplace some papers....Yeah, you can ask the primary nurse but when you go through the file, you can find that it is the paper that was written that is missing.... If it was, if it was, if it was history, you just come and take it from the mother... You go back to the mother.
	(Nurse) The doctor writes.... Yes. They're separate [from the Kardex]. Cause that's the doctor who writes. But now there's some investigations that now you have to confirm. If, if, let's say a blood work was done and I'm giving a report of blood work, which was supposed to be done during my shift, I'm supposed to go back now to the file and see if it was done. Because we don't interact much with the files. It's the doctors what we get there, maybe at the now the ward round itself.

	Observations – hand held papers
	**EQ27: When there is no adequate storage system for patient records on the unit (C) staff will take records with them to different locations as they work (M) leading to misplaced files, frustration, wasted time, incivility (O).**
	(Nurse) So maybe somebody had gone to the lab and he or she has taken time you are supposed to take the patient to theatre… … that delay. So and you are not aware that somebody has gone with that patient file. .. So you are here looking for that file… You can end up opening another one. So it becomes like double work… …because of lack of communication in between our saying like I'm with patients file, I'm doing this and this or even like there is a a carder sometimes they say the consultant, the the second on call such thing the MO. So if the message is not passed down this is what we're going to do this. So you get here down on the ground the message is not well passed.
	**EQ28: When patient records are kept in an orderly manner (e.g. within organised patient groups) (C) 'the right way becomes the easy way' and respect/ valuing of records are enhanced (M) so order will be maintained and quality of documentation will improve (O).**
	(Nurse) So you are just within the room as they go around. If there is anything they will tell you. Then later on you take your report from the files. .. I get my information. Some of them they tell you verbally, I want this hand over here. Like stat-photo. This baby is for blood transfusion, those specific vital procedures.
	**EQ29: Where documentation is integrated into ward rounds (C) it will be an accurate summary (M) and provide a useful reference source for all (O).**
	(Nurse) Yes, we do. And there are major ward rounds, which are particularly on Tuesdays, but thankfully for our unit, every day there is a doctor in every room. So there are rounds that they, they carry through every day. Every day. So with that, they're able to update you on the, the current plan of the, of the patients. And thankfully for documentation, there, there, there is a feeding chart where they always record. There is the files where they, they note the, the round notes. And then for us, it's just copying up to the, the round books. We have the round books where we have indicated each patient and the plan that they, they require. Yeah.
	(Nurse) I can say not, well, we attend but not on a regular basis, but we usually find out what the, what, what the plan, the doctor's plan for that day...... From the file.
	(Nurse) Ward rounds, so we usually attend, but sometimes the work nature cannot allow us to attend. But if there is something a doctor needs you to be involved in, he usually tells us. .... Yes, the doctor will tell you. Because at the time, we have limited time, but with many procedures. At the same time, we have feeding procedure, we have medication. And we have also other things to attend to. And maybe you are one nurse in a room with ten babies, so you cannot work with a doctor from one patient to another patient listening to the doctor's sound. So you are just within the room as they go around. If there is anything they will tell you. Then later on you take your report from the files. .... I get my information. Some of them they tell you verbally, I want this hand over here. Like stat-photo. This baby is for blood transfusion, those specific vital procedures.
	(Nurse) When the doctors do the ward round, whatever plan they have said... they've discussed or they have decided, they normally write it on the file, so you as a nurse...definitely there's a round book you will write. You write on the cardex, then you will implement whatever was written.
**Staff rest and recovery**	**EQ30: When staff feel overwhelmed, or are tired/ hungry (C) they may respond to requests with incivility (O) as they cannot cope and are exhausted (M).**
	(Senior Nurse) .....Those are long duties, and in the morning they are productive. In the afternoon they are irritable because some of them they've not even eaten. They've been in a busy nursery. They didn't have time even to take breakfast. So such a people get so irritable and you get the bad communication..... The afternoon, they're not productive, in the morning, they've worked well and they've worked smart. But since they've just continued working, they didn't have even lunch break. Because sometimes there's only one person in a nursery, she can't go for, for lunch. Who will take care of the nursery. Sometimes she's, there's a busy nursery. So you get such a person who works from morning to evening and you get it become so difficult in the evening. That person is worn out. .... She can't be able to be productive. She does, she has exhausted all her energies or his energy. That way she becomes irritable, even a mother asking something, they tell them for that one I don't know, he can't even, long duties.
	(Nurse) So, when you are tired and when you have burnout, you become mentally disturbed kind of ...so your input may be less, most of the time when you have burnout you become irritated... even communicating with the mother may not be good. You know somebody who has burnout is kind of sick and you may produce little.
	(Nurse) Yes, there is a lot that can be done. Like now you find that we work on shifts and sometimes the night shift is very very tiring. You can find yourself standing the whole night. Until sometimes you are not effective.... Overworking.... You can be overworked because of the shortage. Let us say you find yourself in the room alone with 15 babies, like even here now we have 15 babies. You are alone and the babies a very sick.
	**EQ31: When staff have a rest space with adequate facilities (e.g. fridge, lockers) (C) it will improve satisfaction at work (O) as they feel valued and can refresh and relax during breaks at work (M).**
	Observations
	**EQ32: Protected staff rest areas (C) facilitate more open interactions (O) because staff can step away from the busy clinical environment (M).**

	(Nurse) The discussions [in the tea room] about, maybe we have a case scenario or maybe you are so tormented you see like a patient can come...and this patient is saying it is today or today, this baby must get blood now or never,...and you are so tormented because you are not the one who give out blood, you go for blood ..... Mostly we talk of, it's about the patient only, the things that you are supposed to do or maybe there is something like NNAK [National Nursing Association of Kenya] have launched.....Or maybe even its about maybe the CEO.... maybe there is an information that was, there was some information maybe it was written and maybe you never saw it on the notice board... Somebody can come up with it and is like did you…you people see this?
	(Nurse) It's more social [in the tea room]
	(Nurse) .. and during our breaks. Let's say tea breaks, we tend to, like a, it's an open forum, in that someone poses a question, we discuss...
	**EQ33: When staff are not given adequate protected rest spaces (C) they must adopt workarounds (e.g. changing in corridor) (M) leading to feeling of not being valued (O).**
	Observations
	**EQ34: Where different cadres have different rest spaces (C) interactions between cadres may still be limited (O) as staff will take breaks where they feel most comfortable (M).**
	Observations
	**EQ35: When babies die or when mothers' suffering is witnessed (C) staff are grieved (M) and feel demoralised (O).**
	(Nurse)...And that one sometimes even it overwhelms you when even you go to at home, you find yourself even calling asking baby so and so ako aje? [how are they] how is that baby? Because you feel maybe that baby succumbed…
	(Student Nurse) Maybe when you are thinking of that pathway think about that patient, the mother of that baby. They pass through stress when they see their babies not feeling well.
	(Nurse) You see if a patient dies that one definitely, you, you just, when you are in a room and you have a case, a mortality, you feel that, hey, the day has not been good to you. ..... Very. Very. First of all, when you find that the mother has struggled a lot, you feel like hey, it's the end of the world. Because you see the mothers crying. So psychologically it affects you.
	(Student Nurse) Mostly if something bad happened… like maybe if you we had a maternal or neonatal death
	**EQ36: When babies are viewed as more desirable patients (C) staff will prefer working on NBU (O) as they are serving babies (M).**
	(Student Nurse) It was a nice place to work because, at the, the babies, they don't bother as much as the adults.
	(Senior Nurse) Yeah, we should not put our issues first but patient's first. Then we have the needy babies like the ones the mothers have absconded.... It, now we take them as our babies. They are our real babies because now we do for them everything.
	(Nurse) Well, you know when you deal with the babies you are dealing with, they don't talk, they are not like those adults who will abuse you, and tell you anything, but the babies are just quiet, you have to work on them. Unless you know they come with a mother is not cooperating, but generally you will find it's good.

**Support for adaptation of learned norms**	**EQ37: When staff have trained in a specific hospital setting (C) they will have learned specific norms/ workarounds (O) based on availability of equipment etc on the unit (M).**<br>**EQ38: When staff transfer to a new hospital/ unit (C) they may have to learn new norms/ workarounds (O) as these will have evolved differently based on differences in how resourced the unit is in comparison to their previous unit (M).**<br>**EQ39: When staff transfer to a new unit (C) norms may clash (O) due to differences in workarounds learnt at previous unit, which may have been poorer resourced or smaller (M).**
	(Nurse) Yes, because there are sometimes you can also be talking with your colleagues outside you find that they are doing different things.... Yes, differently from the way…you are doing them. Yeah.
	(Nurse) Okay, okay. I…I have ever met once when I came when I was new and I was you know and I was taking the baby to theatre.... where I had come from we used to use cots and we wrap the baby.... cot, cot, cot and we wrap the baby so well and then it's like you cover the baby you take the baby to theatre, but when I came here it was the other way round, the babies were supposed to be taken by using an incubator.... Yeah. So when I asked one of the senior nurses she told me that it's okay using a cot and when I was almost leaving the room.. Somebody really shouted to me and it was like even I got like now what next. So after that, I just kept quiet look at them, they were there.... shouting
	(Nurse) Now that one, that one I would attribute to maybe where someone did the training....And how active that person was, they may not be the same but one of the things that I've observed in the time that I've been here … mostly in the new born unit it has machines that so many hospitals doesn't have...: So, if probably someone did not rotate here sometimes it's not about knowledge, they have never seen some of these…. yeah equipment, so they tend to be somehow maybe confused a little bit. Now, and also the numbers that we have. You realise that so many hospitals across the country their new born unit is usually very small……with less than 10 babies then you get into a unit where you are getting a hundred plus babies and there are procedures that probably you have never done …maybe you just see it in books, so you… there is that maybe little confusion.
	(Student Nurse) I am empowered to do something there. Probably now like there are procedures that I am empowered to do as a nurse. When you come to these institutions, they have their way of doing things. This should be done by only the consultant or the medical team.... So, you cannot do it. Yes. You have the skill and the knowledge to do it... But despite having the skill and the knowledge you are limited.
	**EQ40: If the clinical training environment is under-resourced (C) students will learn shortcuts/ workarounds instead of the correct way (O) as classroom learning is impossible to implement (M).**
	(Student Nurse) ..when I come to the ground, I don't have the ideal equipment, to do my ideal procedure, how will I do it?... you see now si I'll start doing short cuts, I'll start doing what I've been, what I've found them doing....but at the back of my mind, I know this is not the right thing. This is not what I was taught.... I think it's just availability of resources, it's not...and then time, you know, just, maybe you're overwhelmed, you have so many people, you can't start doing like, these ideal procedures are very long, you know,...so, you can't start doing for every other person.
	(Nurse) ...the availability of resources... let me call that. yeah, because, you know, you know sometimes we say, there is the ideal thing and there is what is on the ground... so, most of the time we don't do the ideal thing, what is in the books... if you see, if you see like a, phototherapy is for one baby, and we're having 8 babies.
	**EQ41: When clinical placements are well resourced (C) student learning experience will improve (O) due to better exposure (M).**<br>**EQ42: If clinical training environment is small (C) learning may be limited (O) by scope of facilities/ set-up (M).**
	(Student Nurse) Exposure for example, I can give an example, if, I can give an example of, or personally us the students. For example, from (name), we have got our rotations in (name of hospital) NBU, there are some students maybe from (name) campus, their access to the NBU, maybe they're going to a sub county hospital, the NBU has got a capacity of like four to five children, and there are limited kind of resources, here you find that our exposure is much better than their exposure. So when they're delivering the upcoming times, it'll be much lower than ours. And also the exposure to the learning materials and everything. Maybe the education system that is being given, on how to manage.
	(Nurse) For the newcomers, the graduates, some of them have not trained within (name of hospital). They come without knowing how they will find (name of hospital) to be. So, wherever they come from, maybe there were less facilities, they find a complicated place. ....Some come from nurseries where there were five babies or two. Those where they trained, maybe they were not nurseries. You are posted here, so we take them as they don't know and we just train them.
	(Nurse) Now that one, that one I would attribute to maybe where someone did the training... And how active that person was, they may not be the same but one of the things that I've observed in the time that I've been here … as I was telling you in X mostly in the new born unit it has machines that so many hospitals doesn't have...: So, if probably someone did not rotate here sometimes it's not about knowledge, they have never seen some of these…. yeah equipment, so they tend to be somehow maybe confused a little bit. Now, and also the numbers that we have. You realise that so many hospitals across the country their new born unit is usually very small……with less than 10 babies then you get into a unit where you are getting a hundred plus babies and there are procedures that probably you have never done …maybe you just see it in books, so you… there is that maybe little confusion.
	**EQ43: When staff employ workarounds on the unit (C) students will learn these as best practice (O) as they cannot implement classroom teaching due to lack of resources on unit (M).**
	(Student Nurse) No, because they can't change because of the situation here. Maybe the ratio of nurses to, to the babies is small, so they're not like able to do the ideal so I should say the ideal. Yeah. Because we're being taught on the ideal, but on the ground things are different.

	(Student Nurse) Yeah. Somehow because back to the drug, [interruptions] like back to the drug, drug part………. So that even when you flush, whatever remains in the line, just remains. Because, another thing, we were being told that, even if you flush, they can't repeat with the sterile water, water for injection, because it'll take a lot of time. Babies are many, that's why they would add up. So, like that one, that one the knowledge that I had in class, so I had to shed it.
	(Student Nurse) when I come to the ground, I don't have the ideal equipment, to do my ideal procedure, how will I do it?... you see now si I'll start doing short cuts, I'll start doing what I've been, what I've found them doing.... but at the back of my mind, I know this is not the right thing. This is not what I was taught... I think it's just availability of resources, it's not..and then time, you know, just, maybe you're overwhelmed, you have so many people, you can't start doing like, these ideal procedures are very long, you know,.. so, you can't start doing for every other person.
	**EQ44: When staff/ students rotate to different units or are not exposed to relevant clinical scenarios (C) they cannot implement or consolidate training (M) leading to wasted efforts from courses/ learning/ education (O).**
	(Nurse) For the newcomers, the graduates, some of them have not trained within (hospital). They come without knowing how they will find (hospital) to be. So, wherever they come from, maybe there were less facilities, they find a complicated place. We just take them the way they come and keep. Actually, we mentor them. We don't just allocate them and leave them. I'm new, you are attached to a qualified staff, you stay with them for one month to mentor you and orient you in the unit. You come to understand what happens in the unit and how you are expected to work in the unit. Some come from nurseries where there were five babies or two. Those where they trained, maybe they were not nurseries. You are posted here, so we take them as they don't know and we just train them.
	(Nurse) So, if probably someone did not rotate here sometimes it's not about knowledge, they have never seen some of these… yeah equipment, so they tend to be somehow maybe confused a little bit. Now, and also the numbers that we have. You realise that so many hospitals across the country their new born unit is usually very small …with less than 10 babies then you get into a unit where you are getting a hundred plus babies and there are procedures that probably you have never done …maybe you just see it in books, so you… there is that maybe little confusion.

E: RAMESES II Reporting Checklist for Realist Evaluations

Taken from: Wong, G., Westhorp, G., Manzano, A. *et al.* RAMESES II reporting standards for realist evaluations. *BMC Med* **14**, 96 (2016). https://doi.org/10.1186/s12916-016-0643-1

**TITLE**			**Reported in document Y/N/Unclear**	**Page(s) in document**
1		In the title, identify the document as a realist evaluation	Y	1
SUMMARY OR ABSTRACT				
2		Journal articles will usually require an abstract, while reports and other forms of publication will usually benefit from a short summary. The abstract or summary should include brief details on: the policy, programme or initiative under evaluation; programme setting; purpose of the evaluation; evaluation question(s) and/or objective(s); evaluation strategy; data collection, documentation and analysis methods; key findings and conclusions<br>Where journals require it and the nature of the study is appropriate, brief details of respondents to the evaluation and recruitment and sampling processes may also be included<br>Sufficient detail should be provided to identify that a realist approach was used and that realist programme theory was developed and/or refined	Y	2
INTRODUCTION				
3	Rationale for evaluation	Explain the purpose of the evaluation and the implications for its focus and design	Y	4,5
4	Programme theory	Describe the initial programme theory (or theories) that underpin the programme, policy or initiative	Y	6

TITLE			Reported in document Y/N/Unclear	Page(s) in document
5	Evaluation questions, objectives and focus	State the evaluation question(s) and specify the objectives for the evaluation. Describe whether and how the programme theory was used to define the scope and focus of the evaluation	Y	6
6	Ethical approval	State whether the realist evaluation required and has gained ethical approval from the relevant authorities, providing details as appropriate. If ethical approval was deemed unnecessary, explain why	Y	32
METHODS				
7	Rationale for using realist evaluation	Explain why a realist evaluation approach was chosen and (if relevant) adapted	Y	5,7
8	Environment surrounding the evaluation	Describe the environment in which the evaluation took place	Y	4,5
9	Describe the programme policy, initiative or product evaluated	Provide relevant details on the programme, policy or initiative evaluated	N/A	
10	Describe and justify the evaluation design	A description and justification of the evaluation design (i.e. the account of what was planned, done and why) should be included, at least in summary form or as an appendix, in the document which presents the main findings. If this is not done, the omission should be justified and a reference or link to the evaluation design given. It may also be useful to publish or make freely available (e.g. online on a website) any original evaluation design document or protocol, where they exist	Y	7-11 (+ published protocol cited)

TITLE			Reported in document Y/N/Unclear	Page(s) in document
11	Data collection methods	Describe and justify the data collection methods – which ones were used, why and how they fed into developing, supporting, refuting or refining programme theory<br>Provide details of the steps taken to enhance the trustworthiness of data collection and documentation	Y	7-11
12	Recruitment process and sampling strategy	Describe how respondents to the evaluation were recruited or engaged and how the sample contributed to the development, support, refutation or refinement of programme theory	Y	8,9
13	Data analysis	Describe in detail how data were analysed. This section should include information on the constructs that were identified, the process of analysis, how the programme theory was further developed, supported, refuted and refined, and (where relevant) how analysis changed as the evaluation unfolded	Y	10,11
RESULTS				
14	Details of participants	Report (if applicable) who took part in the evaluation, the details of the data they provided and how the data was used to develop, support, refute or refine programme theory	Y	12
15	Main findings	Present the key findings, linking them to contexts, mechanisms and outcome configurations. Show how they were used to further develop, test or refine the programme theory	Y	12-28
DISCUSSION				
16	Summary of findings	Summarise the main findings with attention to the evaluation questions, purpose of the evaluation, programme theory and intended audience	Y	28

TITLE			Reported in document Y/N/Unclear	Page(s) in document
17	Strengths, limitations and future directions	Discuss both the strengths of the evaluation and its limitations. These should include (but need not be limited to): (1) consideration of all the steps in the evaluation processes; and (2) comment on the adequacy, trustworthiness and value of the explanatory insights which emerged<br>In many evaluations, there will be an expectation to provide guidance on future directions for the programme, policy or initiative, its implementation and/or design. The particular implications arising from the realist nature of the findings should be reflected in these discussions	Y	29-31
18	Comparison with existing literature	Where appropriate, compare and contrast the evaluation's findings with the existing literature on similar programmes, policies or initiatives	Y	29,30
19	Conclusion and recommendations	List the main conclusions that are justified by the analyses of the data. If appropriate, offer recommendations consistent with a realist approach	Y	31
20	Funding and conflict of interest	State the funding source (if any) for the evaluation, the role played by the funder (if any) and any conflicts of interests of the evaluators	Y	32,33
